# Clinical Effectiveness of Intravitreal Therapy With Ranibizumab vs Aflibercept vs Bevacizumab for Macular Edema Secondary to Central Retinal Vein Occlusion: A Randomized Clinical Trial

**DOI:** 10.1001/jamaophthalmol.2019.3305

**Published:** 2019-08-29

**Authors:** Philip Hykin, A. Toby Prevost, Joana C. Vasconcelos, Caroline Murphy, Joanna Kelly, Jayashree Ramu, Barry Hounsome, Yit Yang, Simon P Harding, Andrew Lotery, Usha Chakravarthy, Sobha Sivaprasad

**Affiliations:** 1 National Institute for Health Research, Moorfields Biomedical Research Centre, London, United Kingdom; 2 Imperial Clinical Trials Unit, School of Public Health, Imperial College London, London, United Kingdom; 3 King's Clinical Trials Unit, King’s Health Partners, King’s College London, London, United Kingdom; 4 Wolverhampton Eye Infirmary, Wolverhampton, United Kingdom; 5 Eye and Vision Science, University of Liverpool, St Paul’s Eye Unit, Royal Liverpool University Hospitals, Members of Liverpool Health Partners, Liverpool, United Kingdom; 6 Department of Medicine, University of Southampton, Southampton, United Kingdom; 7 Biomedical Sciences, Queen’s University of Belfast, Belfast, United Kingdom

## Abstract

**Importance:**

The comparative clinical effectiveness of ranibizumab, aflibercept, and bevacizumab for the management of macular edema due to central retinal vein occlusion (CRVO) is unclear.

**Objective:**

To determine whether intravitreal aflibercept or bevacizumab compared with ranibizumab results in a noninferior mean change in vision at 100 weeks for eyes with CRVO-related macular edema.

**Design, Setting, and Participants:**

This prospective, 3-arm, double-masked, randomized noninferiority trial (Lucentis, Eylea, Avastin in Vein Occlusion [LEAVO] Study) took place from December 12, 2014, through December 16, 2016, at 44 UK National Health Service ophthalmology departments. Inclusion criteria included age 18 years or older, visual impairment due to CRVO-related macular edema of less than 12 months with best-corrected visual acuity (BCVA) Early Treatment Diabetic Retinopathy Study letter score (approximate Snellen equivalent) in the study eye between 19 (20/400) and 78 (20/32), and spectral domain optical coherence tomography imaging central subfield thickness of 320 μm or greater. Data were analyzed from March 4, 2019, to April 26, 2019.

**Interventions:**

Participants were randomized (1:1:1) to receive repeated intravitreal injections of ranibizumab (0.5 mg/0.05 mL) (n = 155), aflibercept (2.0 mg/0.05 mL) (n = 154), or bevacizumab (1.25 mg/0.05 mL) (n = 154) for 100 weeks.

**Main Outcomes and Measures:**

Adjusted mean change in BCVA in the study eye at 100 weeks wherein noninferiority was concluded if the lower bounds of the 95% CI of both the intention-to-treat and the per protocol analyses were above –5 letters.

**Results:**

Of 463 participants, 265 (57.2%) were male, with a mean (SD) age of 69.1 (13.0) years. The mean (SD) gain in BCVA letter score was 12.5 (21.1) for ranibizumab, 15.1 (18.7) for aflibercept, and 9.8 (21.4) for bevacizumab at 100 weeks. Aflibercept was noninferior to ranibizumab (intention-to-treat–adjusted mean BCVA difference, 2.23 letters; 95% CI, –2.17 to 6.63 letters; *P* < .001). Bevacizumab was not noninferior to ranibizumab (intention-to-treat–adjusted mean BCVA difference, –1.73 letters; 95% CI, –6.12 to 2.67 letters; *P* = .07). The per protocol analysis conclusions were similar. Fewer mean injections were given in the aflibercept group (10.0) than in the ranibizumab (11.8) group (mean difference at 100 weeks, –1.9; 95% CI, –2.9 to –0.8).

**Conclusions and Relevance:**

Mean changes in vision after treatment of macular edema due to CRVO were no worse using aflibercept compared with ranibizumab. Mean changes in vision using bevacizumab compared with ranibizumab were inconclusive regarding vision outcomes (ie, the change in visual acuity from baseline, on average, may be worse or may not be worse when using bevacizumab compared with ranibizumab).

**Trial Registration:**

ISRCTN13623634

## Key Points

**Question:**

Does intravitreal aflibercept or bevacizumab result in a noninferior mean change in vision at 100 weeks compared with ranibizumab for eyes with central retinal vein occlusion–related macular edema?

**Findings:**

In this randomized clinical trial of 463 individuals with central retinal vein occlusion–related macular edema from 44 UK National Health Service ophthalmology departments, aflibercept treatment was noninferior (no worse than) ranibizumab treatment at 100 weeks and the results for bevacizumab vs ranibizumab were not noninferior (ie, inconclusive compared with the ranibizumab group).

**Meaning:**

This is important information for ophthalmologists to consider before treating such cases.

## Teaser

This randomized clinical trial compares the clinical effectiveness of intravitreal therapy with ranibizumab vs aflibercept vs bevacizumab for the management of macular edema secondary to central retinal vein occlusion.

## Introduction

Central retinal vein occlusion (CRVO) has a prevalence of 0.08%,^1,2^ and visual impairment due to macular edema is unlikely to improve spontaneously.^3,4,5^ Macular edema is treated with repeated intravitreal injections of the licensed anti–vascular endothelial growth factor (anti-VEGF) agents ranibizumab and aflibercept,^6,7,8,9,10^ with the unlicensed low-cost alternative bevacizumab also being used widely.^11,12,13^ The findings from the Study of Comparative Treatments for Retinal Vein Occlusion 2 (SCORE2)^14^ reported bevacizumab to be noninferior to aflibercept with respect to visual acuity at 6 months. In noncomparative CRVO studies, first-year anti-VEGF therapy vision gains were maintained in the second year with bimonthly but not trimonthly monitoring and treatment.^7,15,16^ The Lucentis, Eylea, Avastin in Vein Occlusion (LEAVO) trial^17^ (trial protocol in Supplement 1) evaluated the comparative clinical effectiveness of anti-VEGF monotherapies in CRVO-related macular edema during 100 weeks.

## Methods

### Study Design

The LEAVO trial^17^ was a multicenter, prospective, 3-arm, double-masked, randomized, noninferiority trial that recruited patients from 44 UK National Health Service hospitals from December 12, 2014, through December 16, 2016. The trial steering and data monitoring committees provided independent oversight. The study was approved by the UK National Research Ethics Committee Service. Patients provided written informed consent. The standard National Health Service treatment was ranibizumab at the time of the study design, which aimed to answer whether aflibercept and bevacizumab were each noninferior to ranibizumab, with the noninferiority limit defined as –5 Early Treatment Diabetic Retinopathy Study (ETDRS) letters. Because aflibercept was licensed after the protocol was finalized, the data monitoring committee permitted a post hoc analysis to compare aflibercept and bevacizumab.

### Participants

Adults with visual impairment due to CRVO-related macular edema of less than 12 months’ duration with best corrected visual acuity (BCVA) ETDRS letter score (approximate Snellen equivalent) in the study eye between 19 (20/400) and 78 (20/32) and spectral-domain optical coherence tomography (OCT) imaging central subfield thickness (CST) of 320 μm or greater were included (Supplement 1 and eTable 1 in Supplement 2).

### Randomization and Masking

Eligible patients were randomly allocated (1:1:1) to ranibizumab, aflibercept, or bevacizumab by the method of minimization using a web-based randomization service and the following factors: BCVA letter score (19-38, 39-58, or 59-78), disease duration (<3 months, 3-6 months, or >6 months), and treatment naive or not. Patients, optometrists (the primary outcome assessors), clinical investigators, and imaging technicians were masked to treatment allocation. Optometrists and patients completed a treatment allocation guess form at study exit to determine masking effectiveness.

### Interventions

Study eyes were randomized 1:1:1 to intravitreal ranibizumab, 0.5 mg/0.05 mL (Novartis), aflibercept, 2 mg/0.05 mL (Bayer Pharma AG; both supplied from routine National Health Service hospital stock), and bevacizumab, 1.25 mg/0.05 mL (Roche; supplied by the Royal Liverpool & Broadgreen Pharmacy Aseptic Unit).

### Procedures

Participants in all study groups had mandated injection at baseline and 4, 8, and 12 weeks. From week 16 through 96, treatment was given if 1 or more of the following retreatment criteria were met: a decrease in the BCVA letter score of more than 5 between the current and most recent visit that was attributed to an increase in OCT CST, an increase in BCVA letter score of more than 5 between the current and most recent visit, OCT CST of ≥320 μm of greater (Heidelberg, Spectralis, or >300 μm for alternatives) because of intraretinal or subretinal fluid, and OCT CST increase of more than 50 μm from the lowest previous measurement. Visits at weeks 16 and 20 were mandated; from week 24, the visit interval could be increased from 4 weeks to 8 weeks if retreatment criteria were not met at 3 consecutive visits. Retreatment was withheld if the BCVA letter score was more than 83 letters. The BCVA was measured at 4 m using BCVA charts from the ETDRS with refraction at baseline; 12, 24, 52, 76, and 100 weeks; and study withdrawal.

### Outcomes

The primary outcome was the change in BCVA letter score from baseline to 100 weeks in the study eye for each intervention compared with ranibizumab. Secondary outcomes in the study eye included a gain of at least 10 BCVA letters at 52 weeks and at least 15 BCVA letters at 100 weeks, losses of 15 or fewer at 52 weeks or at least 30 BCVA letters at 100 weeks, change in OCT CST from baseline to 52 and to 100 weeks, OCT CST less than 320 μm at 52 and 100 weeks, and the number of injections by 100 weeks. Adverse events were recorded throughout 100 weeks.

### Sample Size and Statistical Analysis

The SD of the change in visual acuity after adjustment for baseline was anticipated to be 14.3 based on available data at 52 weeks in a relevant treated population.^3^ The LEAVO trial had at least 80% power to detect noninferiority of –5 letters for each investigative treatment compared with the standard of ranibizumab using a 2-sided 95% CI from an analysis of covariance test with adjustment for baseline visual acuity. Demonstrated noninferiority allowed a subsequent test of superiority without needing type I error correction. The sample size was set to be 459 participants. The intention-to-treat (ITT) population was defined to comprise all patients as randomized. The primary outcome of refracted BCVA was compared between aflibercept and ranibizumab and between bevacizumab and ranibizumab. The groups were assessed primarily at the 100-week point adjusting for baseline using a linear mixed-effects model allowing for within-patient correlation of repeated measures over time using an unstructured covariance matrix. All participants with at least 1 milestone visit were included in the model. Fixed effects included the main effects and interactions with time (defined as milestone visits at 12, 24, 52, 76, and 100 weeks) in the treatment group, disease duration (<3 and ≥3 months), the baseline of the outcome, and its missing indicator required for the missing indicator method.^18,19^ The test for noninferiority was 1-sided at the 2.5% significance level and presented as an estimated effect with 2-sided 95% CIs compared with the noninferiority margin of –5 letters. The per protocol (PP) population was defined as a subset of the ITT population who were eligible and received minimal sufficient treatment exposure, defined as 4 treatments correctly assessed and received during the first 6 visits. For the analysis of the primary outcome, the mixed-effects model was refitted within the PP population. Noninferiority was declared if the estimated 95% CI for the difference in means was above the margin of –5 letters in both the ITT and PP analysis models primarily at 100 weeks and secondarily at 52 weeks (and implicitly 1-sided *P* < .02 for both). Analyses were completed according to the ITT strategy under a missing-at-random assumption together with principled sensitivity analysis in the full ITT and PP populations.^20,21^ This assessed sensitivity to the handling of missing 100-week data using 3 recommended scenarios affecting any or all groups. Secondary continuous outcomes were analyzed only on the ITT basis, for superiority, and with the same model specification as for the primary outcome except with baseline BCVA represented by its minimization categories and reported as adjusted differences in means. Safety and Anti-Platelet Trialists’ Collaboration events were reported as proportions and compared between groups with Wilson 95% CIs for rare events. All superiority tests were 2-sided at the 5% significance level and effect sizes interpreted cautiously with 95% CIs.

Sensitivity analysis was used to assess the robustness of the primary outcome conclusions to the effects of missing data (eFigure 1 in Supplement 2) and to the use of concomitant treatments or outliers (affecting only 1 patient each). Subgroup analyses for primary outcomes involved extending the models to include interaction terms with group for the subgroup variable at all time points. Originally, subgroup variables were to be the categories of factors in the minimization baseline BCVA letter score (19-38, 39-58, and 59-78), disease duration at screening (<3, 3-6, and >6 months), and treatment naive vs previously treated. The statistical analysis plan was reapproved after recategorizing disease duration (<3 and ≥3 months) and removing the treatment naive vs previously treated variable because of very low numbers.

## Results

**Figure 1.**
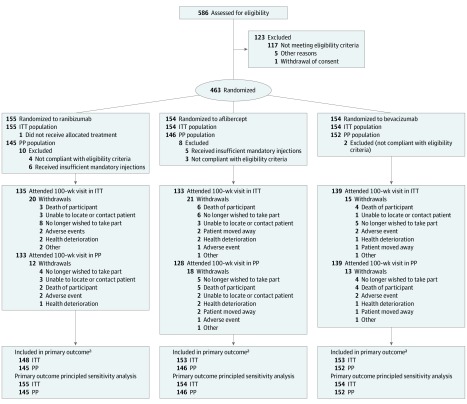
CONSORT Diagram for the LEAVO Trial ITT indicates intention-to-treat; LEAVO trial, Lucentis, Eylea, Avastin in Vein Occlusion trial; PP, per protocol. ^a^Models include all participants who have had at least 1 follow-up milestone visit.

Between December 12, 2014, and December 16, 2016, 587 patients were assessed for eligibility and 463 were randomly assigned and allocated to receive ranibizumab (n = 155), aflibercept (n = 154), or bevacizumab (n = 154). Of 463 total participants, 198 (42.8%) were female, with a mean (SD) age of 69.1 (13.0) years. Baseline characteristics were well balanced between treatment groups (Table). A total of 454 participants were included in the prespecified ITT linear mixed effect models and 443 participants in the prespecified PP linear mixed effect models (Figure 1); the 100-week visit was completed by 135 patients (87.1%) in the ranibizumab group, 133 (86.4%) in the aflibercept group, and 139 (90.3%) in the bevacizumab group.

**Table.** Baseline Ocular and Systemic Characteristics^a^

Characteristic	Total (N = 463)	Ranibizumab Group (n = 155)	Aflibercept Group (n = 154)	Bevacizumab Group (n = 154)
Age, mean (SD), y	69.1 (13.0)	69.2 (13.0)	68.7 (13.2)	69.3 (12.8)
Male	265 (57.2)	85 (54.8)	94 (61.0)	86 (55.8)
Study eye, right eye	226 (48.8)	81 (52.3)	67 (43.5)	78 (50.6)
BCVA letter score in the study eye, mean (SD)^b^^,^^c^	54.1 (14.8)	53.6 (15.1)	54.1 (15.3)	54.4 (14.2)
BCVA letter score in the study eye				
19-38	85 (18.4)	31 (20.0)	27 (17.5)	27 (17.5)
39-58	166 (35.9)	56 (36.1)	55 (35.7)	55 (35.7)
59-78	212 (45.8)	68 (43.9)	72 (46.8)	72 (46.8)
Duration of CRVO, median (IQR), mo^b^	0.9 (0.4-1.7)	0.9 (0.5-1.8)	0.9 (0.4-1.7)	0.9 (0.4-1.7)
Duration of CRVO in the study eye, mo				
<3	401 (86.6)	134 (86.5)	129 (83.8)	138 (89.6)
3-6	38 (8.2)	11 (7.1)	19 (12.3)	8 (5.2)
>6	24 (5.2)	10 (6.5)	6 (3.9)	8 (5.2)
Previous treatment of study eye^a^				
None	446 (96.3)	148 (95.5)	149 (96.8)	149 (96.8)
Anti-VEGF therapy	16 (3.5)	6 (3.9)	5 (3.2)	5 (3.2)
CRVO ischemic status at baseline in study eye^b^				
Nonischemic	406 (87.7)	137 (88.4)	135 (87.7)	134 (87.0)
Ischemic	56 (12.1)	17 (11.0)	19 (12.3)	20 (13.0)
OCT in study eye, mean (SD)^b^^,^^d^				
Central subfield thickness, μm	693.6 (209.8)	731.3 (227.6)	673.2 (189.4)	676.1 (207.0)
Total volume, mm^3^	12.7 (2.8)	13 (2.9)	12.3 (2.6)	12.8 (2.9)
Lens status of study eye				
Cataract	131 (28.4)	41 (26.6)	44 (28.6)	46 (29.9)
Pseudophakia	68 (14.7)	29 (18.8)	20 (13.0)	19 (12.3)
Blood pressure, mean (SD), mm Hg^b^				
Systolic	143.0 (16.8)	143.1 (17.6)	142.6 (17.0)	143.1 (15.7)
Diastolic	79.7 (10.4)	80.1 (10.2)	79.1 (10.6)	79.9 (10.6)

Abbreviations: BCVA, best-corrected visual acuity; CRVO, central retinal vein occlusion; IQR, interquartile range; OCT, optical coherence tomography; anti-VEGF, anti–vascular endothelial growth factor.

^a^ Data are presented as number (percentage) of patients unless otherwise indicated.

^b^ Not recorded for 1 patient receiving ranibizumab who was randomized in error.

^c^ For 1 participant in each arm, the baseline best-refracted visual acuity test was incomplete. Test was not performed.

^d^ For total volume, data were missing for 2 patients receiving ranibizumab and 1 patient receiving bevacizumab.

### Visual Acuity Outcomes

**Figure 2.**
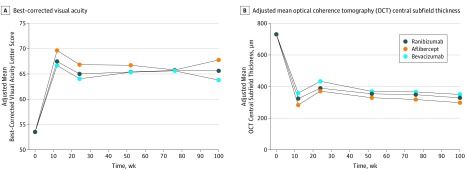
Adjusted Mean Best-Corrected Visual Acuity Letter Score and Adjusted Mean Optical Coherence Tomography (OCT) Central Subfield Thickness Across Groups to 100 Weeks A, Adjusted mean difference between groups at 100 weeks: aflibercept vs ranibizumab, –29.3 (95% CI, –60.9 to 2.3); bevacizumab vs ranibizumab, –21.9 (95% CI, –9.7 to 53.4). B, Adjusted mean difference between groups at 100 weeks: aflibercept vs ranibizumab, –29.3 (95% CI, –60.9 to 2.3); bevacizumab vs ranibizumab, 21.9 (95% CI, –9.7 to 53.4).

**Figure 3.**
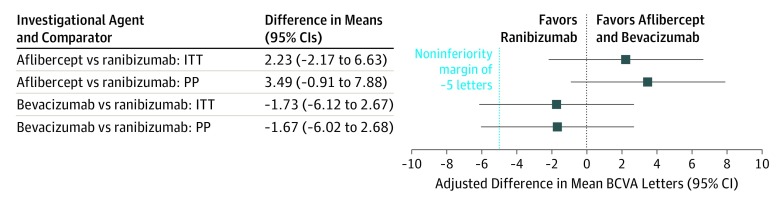
Forest Plot of the Primary Outcome Intention-to-Treat (ITT) and Per Protocol (PP) Analyses BCVA indicates best-corrected visual acuity.

The mean (SD) gain in the BCVA letter score was 12.5 (21.1) for ranibizumab, 15.1 (18.7) for aflibercept, and 9.8 (21.4) for bevacizumab at 100 weeks (Figure 2A). The ITT primary outcome at 100 weeks showed that bevacizumab was not noninferior compared with ranibizumab. However, aflibercept was noninferior but not superior to ranibizumab (Figure 3). The conclusions from the PP analyses were the same (eTable 2 in Supplement 2). The 2 primary noninferiority conclusions remained unchanged if a conservative Bonferroni correction with a 1-sided significance level of 1.25% was used. At 52 weeks, both aflibercept and bevacizumab were noninferior to ranibizumab.

The mean (SD) visual gains by 24 weeks from baseline were 11.4 (19.3) in the ranibizumab group, 13.4 (16.4) in the aflibercept group, and 10.4 (16.6) in the bevacizumab group. The mean BCVA letter score at week 24 decreased by approximately 3 letters across groups after pro re nata (PRN) injections at weeks 16 and 20. Fewer injections were given at those times (total for ranibizumab injections, 123; aflibercept, 76; and bevacizumab, 121), but the number of injections increased gradually thereafter across groups to week 100, during which period patients were seen every 4 to 8 weeks and injected promptly if retreatment criteria were met (Figure 2A).

The proportion of patients across groups with at least 15 BCVA letter gain (ranibizumab, 47%; aflibercept, 52%; and bevacizumab, 45%) was similar (eFigure 2 in Supplement 2), and no group had more than 6% of patients with a loss of at least 30 BCVA letters at week 100. There were no statistically significant differences across groups in the proportion of patients with at least 10 BCVA letter gain or less than 15 BCVA letters loss (eTable 3 in Supplement 2).

In the prespecified subgroup analyses, there were no statistically significant differences across subgroups in baseline BCVA letter score (19-38, 39-58, and 59-78) and disease duration (<3 and ≥3 months) for primary outcomes.

### OCT Outcomes

The mean change in OCT CST from baseline to 100 weeks was –405 μm (95% CI, –450 to –360 μm) in the ranibizumab group, –378 μm (95% CI –412 to –343 μm) in the aflibercept group, and –334 μm (95% CI, –374 to –293 μm) in the bevacizumab group. There were no statistically significant differences across treatment groups for the adjusted difference in CST at 100 weeks for aflibercept vs ranibizumab (–29.3; 95% CI, –60.9 to 2.3) and bevacizumab vs ranibizumab (21.9; 95% CI, –9.7 to 53.4). The mean (SD) OCT reductions by 24 weeks from baseline were 344 (273) μm in the ranibizumab group, 319 (248) μm in the aflibercept group, and 263 (221) μm in the bevacizumab group. The adjusted mean OCT CST across groups increased by approximately 50 μm after PRN visits at weeks 16 and 20, closely mirroring the visual acuity data, and decreased gradually thereafter to week 100 (Figure 2B). There was a significantly greater proportion of patients with an OCT CST of less than 320 μm in the aflibercept group than in the ranibizumab group at 100 weeks (81% vs 66%; mean difference, 15.3% [95% CI, 4.9%-25.7%) and at 52 weeks (76% vs 63%; mean difference, 12.4%; 95% CI, 1.7%-23.1%). The mean difference between the bevacizumab and ranibizumab groups in the proportion of patients with an OCT CST<320 μm was significant at week 24 only (–18.7%; 95% CI, –30.1% to –7.4%) (eFigure 3 in Supplement 2).

### Injections

**Figure 4.**
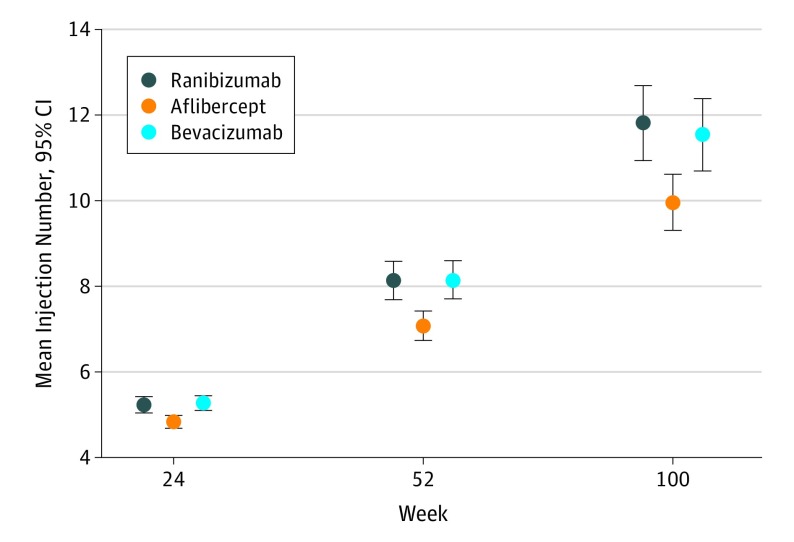
Mean Number of Injections Across Treatment Groups by Week Markers indicate means and whiskers, 95% CIs.

By 100 weeks, patients in the ranibizumab group had received a mean of 11.8 injections (95% CI, 10.9-12.7) compared with 10.0 injections (95% CI, 9.3-10.6) in the aflibercept and 11.5 injections (95% CI, 10.7-12.4) in the bevacizumab groups. The difference between the aflibercept and ranibizumab groups was statistically significant at week 24 (mean difference, –0.4; 95% CI, –0.6 to –0.2), week 52 (–1.1; 95% CI, –1.6 to –0.5), and week 100 (–1.9; 95% CI, –2.9 to –0.8) (Figure 4).

The optometrists assessing primary outcomes provided a response to the treatment allocation guess form for 409 of 463 patients. Among 356 of 409 patients, they responded that they did not know; among 18 of the remaining 53 patients (consistent with chance), they correctly stated the group to which they believed the patient was assigned. For 409 patients, 406 provided a response, 386 did not know, and 8 of 20 correctly stated the group to which they were assigned.

### Bevacizumab vs Aflibercept Exploratory Post Hoc Analysis

A post hoc analysis showed that bevacizumab was not noninferior compared with aflibercept in the ITT analysis at 100 weeks (adjusted mean BCVA difference, –3.96 letters; 95% CI, –8.34 to 0.42; *P* = .32) and at 52 weeks (adjusted mean difference, –1.35 letters; 95% CI, –5.29 to 2.59). The PP analysis conclusion was the same. At 100 weeks, there was a significant difference in the mean number of injections received for bevacizumab compared with aflibercept (1.6; 95% CI, 0.5- 2.7).

### Adverse Events

During the study, based on clinical examination, 25 (5%) eyes developed an ischemic CRVO, 13 eyes (3%) developed anterior segment neovascularization, and 6 eyes (1%) developed retinal neovascularization, with no statistically significant difference across groups. Of 463 eyes in the study: 8 (5.2%) in the ranibizumab group, 7 (4.5%) in the aflibercept group, and 8 (5.2%) in the bevacizumab group were treated with panretinal photocoagulation. There was 1 case of infectious endophthalmitis. The frequency of all ocular adverse events and Anti-Platelet Trialists’ Collaboration–defined events was similar among the groups (eTable 4 in Supplement 2).

## Discussion

This study showed that repeated intravitreal injection of 3 anti-VEGF agents markedly improved and maintained BCVA among patients with macular edema secondary to CRVO during follow-up of 100 weeks. Furthermore, it demonstrated that bevacizumab was not noninferior to ranibizumab and that aflibercept was noninferior to ranibizumab but also not superior. The BCVA data between milestone visits at weeks 12 to 24 identified a decrease in visual acuity across groups of approximately 3 ETDRS letters and likely reflects PRN treatments at weeks 16 and 20 in which fewer injections were given in all 3 groups, particularly the aflibercept arm. These mandated visits and PRN treatments were designed to reduce injection burden based on the CRUISE study,^3^ which suggested a stabilization of BCVA after 4 mandated injections. The visual gains by 24 weeks (eg, mean [SD] in the aflibercept group, 13.4 [16.4]) were, however, less than those reported in COPERNICUS^7^ (mean [SD], 17.3 [12.8]) and SCORE2^14^ (mean, 18.21; 95% CI, 15.71-20.72), in which 6 mandated injections were given. Therefore, we speculated that 6 injections in the loading phase may have led to a greater initial BCVA improvement, although it is possible that recruitment of patients with ischemic CRVO with limited potential for improvement and a ceiling effect because of the upper baseline BCVA letter score of 78 in LEAVO may have contributed to these differences. However, visual acuity gains increased from week 24 and were maintained to 100 weeks for the first time in a comparative study, to our knowledge. This finding suggests that the second-year follow-up regimen of 4 to 8 weekly visits and retreatment criteria using both increases and decreases in visual acuity, coupled with OCT findings, might be more appropriate compared with follow-up every 3 months^7,16^ and may minimize second-year injections by identifying and promptly treating at-risk patients.

All 3 anti-VEGF agents caused a rapid decrease in OCT CST owing to macular edema–related CRVO during the mandated injection phase of this study, consistent with previous trials.^3,4,5,9,14^ There was an adjusted mean OCT CST increase at weeks 16 and 20 that was associated with fewer injections being administered, and thus the mean CST reductions were less than in SCORE 2 at 6 months (eg, aflibercept: SCORE2, –425 μm; LEAVO, –319 μm). The mean CST gradually decreased thereafter in all groups and was consistent with visual acuity data in our study, in contrast to SCORE2, in which OCT data did not closely reflect visual acuity changes. The significantly greater proportion of patients with a CST OCT of less than 320 μm at weeks 52 and 100 with aflibercept compared with ranibizumab is a novel finding in CRVO. Bevacizumab was no less effective than ranibizumab in reducing OCT CST due to CRVO–related macular edema, unlike for other retinal disorders.^20,21,22^

Fewer injections were required for aflibercept compared with ranibizumab, an observation that was evident as early as 24 weeks, and the number of injections increased by 100 weeks. Such a difference in anti-VEGF agent injection has not been previously reported in macular edema due to CRVO and would be a potential advantage to aflibercept use in similar populations.

The post hoc visual acuity analysis showed that bevacizumab was not noninferior compared with aflibercept at both 52 and 100 weeks, consistent with the preplanned primary outcome analyses. The incidence of adverse events was low overall, similar in the 3 treatment groups, and consistent with previous studies of CRVO,^3,4,5,7^ although the study was too small to identify uncommon local and systemic drug-related adverse effects.

### Strengths and Limitations

The robust randomized clinical trial design, broad eligibility criteria, structured 100-week follow-up with a pragmatic retreatment algorithm, and high statistical power were study strengths. The interpretation of the results must be made in the context of the eligibility criteria and treatment protocol. The trial may have included patients with limited potential for visual acuity improvement, including those with high baseline BCVA letter scores (approximate Snellen equivalent) of 74 (20/40) to 78 (20/32), resulting in a ceiling effect, and those with low baseline BCVA due to a severe, ischemic CRVO. Two primary outcome noninferiority analyses were performed: aflibercept vs ranibizumab and bevacizumab vs ranibizumab. It may be a limitation that 97.5% CIs (Bonferroni corrected) were not planned for the 2 primary outcomes used. However, their use would not change the noninferiority conclusions. Conclusions from secondary analyses are supportive and should be made with caution because there was no adjustment for multiple testing. Because aflibercept was unlicensed during the study design period, it was considered to be an investigative agent and comparisons with bevacizumab were post hoc.

## Conclusions

In this randomized clinical trial evaluating 3 different anti-VEGF agents, ranibizumab, bevacizumab and aflibercept, treatment for macular edema due to CRVO resulted in improved and sustained visual gains when patients were followed up regularly and treated promptly. Aflibercept treatment was noninferior to (no worse than) ranibizumab treatment at 100 weeks. In contrast, at 100 weeks, bevacizumab treatment was not noninferior to ranibizumab treatment. These results suggest that mean changes in vision are no worse using aflibercept compared with ranibizumab. However, the results also suggest that mean changes in vision using bevacizumab compared with ranibizumab (ie, the change in visual acuity from baseline), may or may not be worse when using bevacizumab compared with ranibizumab. Results of post hoc exploratory analysis also suggest that bevacizumab treatment was not noninferior to aflibercept treatment, but the exploratory nature of this evaluation should be viewed with greater caution compared with the preplanned analyses when considering these results in the management of macular edema from CRVO.

## Notes

Supplement 1. **Trial Protocol**

Supplement 2. **eTable 1.** The LEAVO Study: Eligibility Criteria **eTable 2.** ITT and PP Analyses for the Primary Outcome at 100 Weeks **eTable 3.** Categorical Visual Acuity Outcomes by Group **eTable 4.** Ocular Adverse Events and APTC Events **eFigure 1.** Sensitivity Analyses for the Missing at Random Assumption in the Primary Outcome Analysis Assessing Noninferiority of Aflibercept and for the Missing at Random Assumption in the Primary Outcome Analysis Assessing Noninferiority of Bevacizumab **eFigure 2.** Percentage of Patients With ≥15 ETDRS Letter Improvement at 52 and 100 Weeks **eFigure 3.** Percentage of Patients With OCT<320 μm at 24, 52, and 100 Weeks

Supplement 3. **Data Sharing Statement**

## Notes

Journal Club Slides
