# Supplementary material for: Clinical Effectiveness of Intravitreal Therapy With Ranibizumab vs Aflibercept vs Bevacizumab for Macular Edema Secondary to Central Retinal Vein Occlusion: A Randomized Clinical Trial
Source: JAMA Ophthalmol. 2019 Aug 29;137(11):1256–64. doi: 10.1001/jamaophthalmol.2019.3305 (PMC6865295; doi:10.1001/jamaophthalmol.2019.3305)
Supplement: Supplement 1. — Trial Protocol [file jamaophthalmol-137-1256-s001.pdf]

1

**hgFull title of trial**	A Multicentre Phase III Double-masked Randomised Controlled Non-Inferiority Trial comparing the clinical and cost effectiveness of intravitreal therapy with ranibizumab (Lucentis) vs aflibercept (Eylea) vs bevacizumab (Avastin) for Macular Oedema due to Central Retinal Vein Occlusion (CRVO).
**Short title**	LEAVO
**Version and date of protocol**	Version 4.0, 8th January 2016
**Sponsor:**	Moorfields Eye Hospital NHS Foundation Trust
**Sponsor protocol number**	HYKP1021
**Funder (s) :**	NIHR HTA CET – National Institute for Health Research, Health Technology Assessment Programme, Clinical Trials and Evaluation Stream.
**EudraCT no**	2014-000272-26
**ISRCTN**	13623634
**ACTIVE Interventions:**	Intravitreal Aflibercept and Bevacizumab
**COMPARATOR Intervention:**	Intravitreal Ranibizumab
**Phase of trial**	Phase III
**Sites(s)**	Multi-Site

Chief investigator:
Mr Philip Hykin,
Moorfields Eye Hospital,
City Road, London, EC1V 2PD.
Tel: +44 (0) 207566 2262
Fax: +44 (0) 207 566 2972
e-mail: philip.hykin@moorfields.nhs.uk

Sponsor Representative:
Mrs Maria Hassard, Deputy Director of R&D,
Moorfields Eye Hospital,
City Road, London, EC1V 2PD.
Tel: +44 (0) 207 5662819
Fax: +44 (0) 207 566 2315
e-mail: maria.hassard@moorfields.nhs.uk

Signatures

The Chief Investigator and R&D have discussed this protocol. The investigators agree to perform the investigations and to abide by this protocol.

The investigator agrees to conduct the trial in compliance with the approved protocol, EU GCP and UK Regulations for CTIMPs (SI 2004/1031; as amended), the UK Data Protection Act (1998), the Trust Information Governance Policy (or other local equivalent), the Research Governance Framework (2005' 2nd Edition; as amended), the agreed SOPs, and other regulatory requirements as amended.

Chief investigator
Mr Philip Hykin

Moorfields Eye Hospital	Signature	Date

Sponsor Representative
Mrs Maria Hassard

Moorfields Eye Hospital	Signature	Date

Contents

Signatures ... 2
Contents ... 3
1 Trial personnel ... 6
2 Protocol Synopsis ... 9
3 Introduction ... 12
4 Objectives ... 17
5 Trial design ... 18
6 Selection of Participants ... 20
7 Recruitment ... 22
8 Study procedures and schedule of assessments ... 22
8.1 Informed consent procedure ... 22
8.2 Randomisation procedures ... 22
8.3 Masking ... 23
8.4 Unmasking and emergency unmasking ... 24
8.5 Screening assessment ... 24
8.6 Baseline assessment ... 25
8.7 Subsequent assessments ... 25
8.8 Visit window for study appointments ... 25
8.9 Study assessments and methods ... 25
8.10 Definition of end of trial ... 28
8.11 Discontinuation criteria ... 28
8.12 Stopping rules ... 28
8.13 Flowchart of study assessments ... 29
8.14 Treatment procedures ... 31
8.15 Withdrawal of Subjects ... 35
8.16 Laboratory procedures ... 35
9 Investigational Medicinal product ... 35
9.1 Name and description of all IMPs used in the trial ... 35
9.2 Name and description of the Comparator used in the trial ... 36
9.3 Summary of findings from non-clinical studies ... 36
9.4 Summary of findings from clinical studies ... 36
9.5 Summary of known and potential risks and benefits ... 36

9.6 Source of active intervention and comparator 36

9.7 Accountability procedures for the IMPs and the Comparator 37

9.8 Drug accountability 37

9.9 Description and justification of route of administration and dosage 38

9.10 Dose modifications 38

9.11 Assessment of compliance 39

9.12 Post-trial IMP arrangements 39

9.13 Name and description of each Non-IMP (NIMP) 39

9.14 Concomitant procedures 40

10 Recording and reporting of adverse events and reactions 41

11 Data management and quality assurance 47

12 Record keeping and archiving 49

13 Statistical Considerations 49

13.1 Outcomes 49

13.2 Sample size recruitment 50

13.3 Statistical analysis plan 51

13.4 Randomisation methods 53

13.5 Interim analysis 54

13.6 Other statistical considerations 54

14 Name of Committees involved in trial 54

15 Direct Access to Source Data/Documents 55

16 Ethics and regulatory requirements 55

17 Finance 56

18 Insurance 56

19 Publication policy 57

20 Statement of compliance 57

21 References 58

22 Appendices 59

76 List of abbreviations

Abbreviation	Definition	Abbreviation	Definition
AE	Adverse Event	MO	Macular Oedema
AR	Adverse Reaction	MRC	Medical Research Council
BCVA	Best Corrected Visual Acuity	NetwORC UK	Network of Ophthalmic Reading Centres UK
BP	Blood Pressure	NHS	National Health Service
BSE	Better seeing eye	NICE	National Institute for Health and Care Excellence
CFP	Colour Fundus Photograph	NIHR	National Institute of Health Research
CRVO	Central Retinal Vein Occlusion	NIMPS	Non-investigational medicinal products
CFP	Colour Fundus Photograph	NV	Neovascularisation
CRF	Case Report Form	nvAMD	Neovascular Age Related Macular Degeneration
CSRI	Clinical Service Receipt Inventory	NVA	Neovascularisation of the angle
CST	Central Sub-field Thickness	NVD	Neovascularisation Disc
CTA	Clinical Trial Authorisation	NVE	Neovascularisation elsewhere
CTU	Clinical Trials Unit	NVG	Neovascular glaucoma
CTIMP	Clinical Trial of Investigational Medicinal Product	NVI	Neovascularisation of Iris
DA	Disc areas	OCT	Optical Coherence Tomography
DIBD	Developmental International Birth Date	PDR	Proliferative Diabetic Retinopathy
DMEC	Data Monitoring and Ethics Committee	PI	Principal Investigator
DSU	Decision Support Unit	PIS	Participant Information Sheet
DSUR	Development Safety Update Report	PP	Per protocol
EC	European Commission	PRN	Pro Re Nata
EDC	Electronic Data Capture	PRP	Panretinal photocoagulation
eCRF	Electronic Case Report Form	QA	Quality Assurance
eMC	Electronic Medicines Compendium	QALY	Quality-Adjusted Life Years
EMA	European Medicines Agency	QC	Quality Control
EQ5D	Euro Quality of life questionnaire	QP	Qualified Person for release of trial drug
ETDRS	Early treatment diabetic retinopathy study	RCT	Randomised Control Trial
EU	European Union	RCOphth	Royal College of Ophthalmologists
EUCTD	European Clinical Trials Directive	R&D	Research and Development
EudraCT	European Clinical Trials Database	REC	Research Ethics Committee
FDA	Food and Drug Administration	SAR	Serious Adverse Reaction
FFA	Fundus Fluorescein Angiography	SAE	Serious Adverse Event
GCP	Good Clinical Practice	ScHARR	School of Health and Related Research
GMP	Good Manufacturing Practice	SD-OCT	Spectral-domain optical coherence tomography
GP	General Practitioner	SDV	Source Document Verification
HbA1C	Glycosylated Haemoglobin	SDW	Source Data Worksheets
HQoL	Health related quality of life	SE	Study Eye
ICF	Informed Consent Form	SOP	Standard Operating Procedure
iCRVO	Ischaemic Central Retinal Vein Occlusion	SPC	Summary of Product Characteristics
IMP	Investigational Medicinal Product	SSA	Site Specific Assessment
IOP	Intraocular Pressure	STA	Single Technology Appraisal
ISRCTN	International Standard Randomised Clinical Trials Number	SUSAR	Suspected Unexpected Serious Adverse Reaction
ITT	Intention to treat	TA	Technology Appraisal
KCTU	King's Clinical Trials Unit	TMG	Trial Management Group
logMAR	Logarithm of the Minimum Angle of Resolution	TSC	Trial Steering Committee
MA	Marketing Authorisation	VA	Visual Acuity
MEH	Moorfields Eye Hospital	VFQ 25	Visual Function Questionnaire
MHRA	Medicines and Healthcare products Regulatory Agency		

1 Trial personnel

Position	Name	Address	Email, telephone and fax details
Chief Investigator (CI)	Mr Philip Hykin	NIHR Moorfields Biomedical Research Centre<br>Moorfields Eye Hospital & UCL Institute of Ophthalmology<br>162, City Road, London, EC1V 2PD	e: philip.hykin@moorfields.nhs.uk<br>t: +44 (0)207 566 2262<br>f: +44 (0)207 566 2972
Sponsor's representative	Mrs Maria Hassard	NIHR Moorfields Biomedical Research Centre<br>Moorfields Eye Hospital & UCL Institute of Ophthalmology<br>162, City Road, London, EC1V 2PD	e: maria.hassard@moorfields.nhs.uk<br>t: +44 (0)207 566 2819<br>f: +44 (0)207 566 2315
Co-Investigator	Miss Sobha Sivaprasad	NIHR Moorfields Biomedical Research Centre<br>Moorfields Eye Hospital & UCL Institute of Ophthalmology<br>162, City Road, London, EC1V 2PD	e: sobha.sivaprasad@nhs.net<br>t: +44 (0)7817 886 759<br>f: +44 (0)20 7566 2972
Lead Statistician	Professor Toby Prevost	Department of Primary Care and Public Health Sciences King's College London<br>5th Floor, Capital House<br>42 Weston Street, London SE1 3QD	e: toby.prevost@kcl.ac.uk<br>t: +44 (0)207 848 6799<br>f: +44 (0)207 848 6620
Statistician	Ms Joana Vasconcelos	Department of Primary Care and Public Health Sciences, King's College London<br>5th Floor, Capital House<br>42 Weston Street, London SE1 3QD	e: joana.vasconcelos@kcl.ac.uk<br>t: +44 (0)20 7848 6799<br>f: +44 (0)20 7848 6620
Co-applicant & Operational Director, KCTU	Ms Caroline Murphy	King's College London PO64, M2.06<br>Institute of Psychiatry, King's College London<br>De Crespigny Park, London SE5 8AF.	e: caroline.murphy@kcl.ac.uk<br>t: +44 (0)207 848 5273<br>f: +44 (0)207 848 5229
Co-applicant & Data Management Strategic Lead, KCTU	Miss Joanna Kelly	King's College London PO64, M2.06<br>Institute of Psychiatry, King's College London<br>De Crespigny Park, London SE5 8AF.	e: joanna.kelly@kcl.ac.uk<br>t: +44 (0)207 848 0532<br>f: +44 (0)207 848 5229
Health Economist	Dr Hasan Basarir	ScHARR, University of Sheffield, Regents Court, 30 Regent's Street, Sheffield S14DA, Sheffield	e: h.basarir@sheffield.ac.uk<br>t: +44 (0)114 222 6397<br>f: +44 (0)114 272 4095
Professor of Health Economics	Professor J.E Brazier	Professor of Health Economics and Head of ScHARR, University. of Sheffield, Regents Court, 30 Regent's Street, Sheffield S14DA, Sheffield	e: j.e.brazier@sheffield.ac.uk<br>t: +44 (0)114 222 0726<br>f: +44 (0)114 272 4095
Trial Manager	Trial Manager	KCTU, King's College London PO64, M2.06,<br>Institute of Psychiatry King's College London,<br>De Crespigny Park<br>London SE5 8AF.	e: firstname.lastname@kcl.ac.uk<br>t: tbc<br>f: tbc
Co-applicant	Professor Simon Harding	Faculty of Health and Life Sciences, University of LIverpool, 3rd Floor, UCD Building, Daulby Street, Liverpool L69 3GA.	e: s.p.harding@liv.ac.uk<br>t:0151 706 4532
Co-applicant	Professor Yit Yang	New Cross Hospital, Wolverhampton Road, Heath Town, Wolverhampton, West Midlands WU10.	e: yit.yang@nhs.net<br>t: 01902 307999

Co-applicant	Professor Usha Chakravarthy	Professor of Ophthalmology, Department of. Dentistry and Biomedical Sciences, University of Belfast	e: u.chakravarthy@qub.ac.uk<br>t: 028 906 32636
Co-applicant	Professor Andrew Lotery	Professor of Ophthalmology, Sir Henry Wellcome Laboratories, South Block, Mailbox 806, Level D, Southampton University Hospital, Southampton SO16 6YD.	e: a.j.lotery@soton.ac.uk<br>t: 023 8079 5049<br>f: 07092 25081

78

IMP Production Liverpool and Broadgreen Hospitals Pharmacy Manufacturing Unit	Shakeel Herwitker	Assistant Director of Pharmacy (Manu), Royal Liverpool University Hospital, Prescot Street, Liverpool, Merseyside L78XP	e: shakeel.herwitker@rlbuht.nhs.uk<br>t: 0151 706 3235<br>f: 0151 282 6218
Independent Reading Centre	Ms Clare Newell	Central Angiographic Research Facility, NetwORC UK, Queens University of Belfast, Grovesnor Road, Belfast BT12 6BA, NI	e: carf@qub.ac.uk<br>t: +44 (0) 28 9063 2516<br>f: +44 (0) 28 9063 2666
Emergency unmasking service	ESMS Global<br>Medical Toxicology and Information Services Ltd<br>Mary Sheridan House<br>Guy's Hospital<br>13 St. Thomas Street<br>London SE1 9RY		t: 020 7188 0300
Randomisation system	King's College London<br>PO64, M2.06<br>Institute of Psychiatry<br>King's College London<br>De Crespigny Park<br>London SE5 8AF		t: 020 7848 0532<br><br>e: CTU@kcl.ac.uk

DMEC members

Role in DMEC	Name and position (address and contact details for Chair)
DMEC Chair	Professor A. Sarah Walker, Professor of Statistics<br>Medical Research Council Clinical Trials Unit (MRC CTU) Aviation House, 125 Kingsway, London, WC2B 6NH<br>e:asw@ctu.mrc.ac.uk, t: 020 7670 4726, f: 020 7670 4969
DMEC Member	Professor Bal Dhillon, Professor of Ophthalmology
DMEC Member	Miss Gilli Vafidis, Consultant Ophthalmologist

TMG members

Role	Name
Chief Investigator	Mr Philip Hykin
Co-Lead	Miss Sobha Sivaprasad
Trial Manager, KCTU	Trial Manager
Lead Statistician	Professor Toby Prevost
Trial Statistician	Ms Joana Vasconcelos
Data Management Strategic Lead, KCTU	Miss Joanna Kelly
Operational Director, KCTU	Ms Caroline Murphy

81

TSC members	
**Role in TSC**	**Name and position (address and contact details for Chair)**
TSC Chair	Miss Susan Downes, Consultant Ophthalmologist,<br>Oxford Eye Hospital,<br>Oxford University Hospitals<br>West Wing, John Radcliffe Hospital,<br>Headley Way, Oxford OX3 9DU<br>e: susan.downes@ouh.nhs.uk<br>t: 01865 234735
Chief Investigator	Mr Philip Hykin, Consultant Ophthalmologist,
Co-lead	Miss S. Sivaprasad, Consultant Ophthalmologist,
Independent Member	Mr Riaz Asaria, Consultant Ophthalmologist
Independent Member	Jonathan Byrne, Consultant Cardiologist,
Independent Member	Vanessa Burgess, Assistant Director, Medicines Optimisation, Lambeth CCG
Independent Member	Mr Greg Fell, Consultant in Public Health
Independent Member	Professor Irene Stratton, Senior Statistician
Patient Representative	Maria Dawson, Policy and Campaigns Manager, RNIB
Patient Representative	Mrs Melba Ryde, Patient Representative
Observers	Trial Statistician
	Trial Manager
	Data Manager
	Senior Member(s) of KCTU
	Sponsor Representative
	Professor Andrew Lotery
	Funder Representative

2 Protocol Synopsis

Title: **A Multicentre Phase III Double-masked Randomised Controlled Non-Inferiority Trial comparing the clinical and cost effectiveness of intravitreal therapy with ranibizumab (Lucentis) vs aflibercept (Eylea) vs bevacizumab (Avastin) for Macular Oedema (MO) due to Central Retinal Vein Occlusion (CRVO).**

Short title: LEAVO

Trial medication: Ranibizumab, Aflibercept and Bevacizumab

Phase of trial: III

Objectives: Primary Objectives

1. To determine whether bevacizumab is non-inferior to ranibizumab in treating visual loss due to MO secondary to central retinal vein occlusion
2. To determine whether aflibercept is non-inferior to ranibizumab in treating visual loss due to MO secondary to central retinal vein occlusion

Secondary Objectives (see Section 4.3)

Type of trial: A multicentre Phase III, double-masked, randomised, active-controlled, clinical trial

Trial design and methods: This is a phase III randomised controlled double-masked non-inferiority clinical trial to evaluate the relative clinical and cost-effectiveness of intravitreal bevacizumab and aflibercept compared to ranibizumab in MO due to CRVO at 100 weeks.

One eye only of 459 adult participants (18 years and above) with MO due to CRVO of ≤ 12 months duration will be randomized to bevacizumab [1.25mg in 50ul] vs aflibercept [2.0mg/50ul] vs ranibizumab [0.5mg/50ul] (1:1:1). After mandated administration of treatment in all arms at baseline, 4, 8, and 12 weeks, further intervention will be based on pre-defined MO retreatment criteria.

The primary outcome will be the change in BCVA ETDRS letter score from baseline to 100 weeks . Secondary outcomes will include additional BCVA outcomes, OCT central macular thickness measurements, change from baseline in visual function questionnaire, VFQ-25, use of resources and adverse events. The primary outcome of cost effectiveness analysis will be mean incremental cost per QALY.

Trial duration per participant: 100 weeks

Estimated total trial duration: 178 weeks (recruitment period + follow up period)

Planned trial sites: Multi-centre study of approximately 40 sites.

Total number of Participants: 459 adults

Disease area

Macular oedema (MO) due to central retinal vein occlusion

Main inclusion / exclusion criteria:

Inclusion Criteria:

1. Subjects of either sex aged ≥ 18 years.
2. Clinical diagnosis of centre-involving macular oedema (MO) due to CRVO
3. CRVO of ≤ 12 months duration.
4. Best corrected visual acuity in the study eye ≥ 19 and ≤ 78 ETDRS letters (approximate Snellen VA 3/60 to VA 6/9).
5. Best corrected visual acuity in the non-study eye ≥ 14 ETDRS letters (approximate Snellen VA ≥ 2/60).
6. SD-OCT central subfield thickness (CST) > 320µm (Spectralis) predominantly due to MO secondary to CRVO in the study eye. See appendix 1 for equivalent CST value for alternative SD-OCT machines.
7. Media clarity, pupillary dilatation and subject cooperation sufficient for adequate fundus imaging of the study eye.
8. In cases of bilateral CRVO, if both eyes are potentially eligible, unless the patient prefers otherwise the worst seeing eye will be recruited.

Exclusion Criteria:

The following apply to the study eye only and to the non-study eye only where specifically stated:

1. Macular oedema considered to be due to a cause other than CRVO (e.g. diabetic macular oedema, Irvine-Gass syndrome).

2. An ocular condition is present that, in the opinion of the investigator, might affect macular oedema or alter visual acuity during the course of the study (e.g. vitreomacular traction)

3. Any diabetic retinopathy or diabetic macular oedema at baseline clinical examination of the study eye.

4. Moderate or severe non proliferative diabetic retinopathy (NPDR) or quiescent, treated or active proliferative diabetic retinopathy (PDR) or macular oedema in the non-study eye. Note: Mild NPDR only is permissible in the non-study eye.

5. History of treatment for MO due to CRVO in the past 90 days with intravitreal or peribulbar corticosteroids or in the last 60 days with anti-VEGF drugs or >6 prior anti-VEGF treatments in the previous 12 months.

6. Active iris or angle neovascularisation, neovascular glaucoma, untreated NVD, NVE and vitreous haemorrhage or treatment for these conditions in the last 1 month. .

7. Uncontrolled glaucoma [>30mmHg], either untreated or on anti-glaucoma medication at screening.

8. Any active periocular or intraocular infection or inflammation (e.g. conjunctivitis, keratitis, scleritis, uveitis, endophthalmitis).

Systemic exclusion criteria are:

9. Uncontrolled blood pressure defined as a systolic value > 170mmHg and diastolic value > 110mmHg

10. Myocardial infarction, stroke, transient ischaemic attack, acute congestive cardiac failure or any acute coronary event < 3 months before randomisation.

11. Women of child bearing potential unless using effective methods of contraception throughout the study and for 6 months after their last injection for the trial.

12. Pregnant or lactating women.

13. Males who do not agree to an effective form of contraception for the duration of the study and for 6 months after their last injection for the trial (see section 6.2 for effective methods of contraception).

14. Hypersensitivity to the active ingredients aflibercept, bevacizumab or ranibizumab or any of the excipients of these drugs.

15. Hypersensitivity to Chinese Hamster Ovary (CHO) cell products or other recombinant human or humanised antibodies

16. A condition that, in the opinion of the investigator, would preclude participation in the study.

17. Participation in an investigational trial involving an investigational medicinal product within 90 days of randomisation.

Statistical methodology and analysis: A detailed statistical analysis plan is in place for the trial. This has been approved by the DMEC and TSC. Further details of statistical considerations can be found in Section 13.

3 Introduction

3.1 Background and clinical data

Retinal vein occlusion (RVO) is the second most common retinal vascular disease after diabetic retinopathy (1). Central retinal vein occlusion (CRVO) is characterised by retinal haemorrhages, venous dilatation and tortuosity in all four quadrants of the retina. These clinical appearances are due to venous obstruction and the resultant increase in hydrostatic pressure causes macula oedema (MO) and ischaemia with reduced visual acuity, which is typically more severe in CRVO. The usual presentation of CRVO is sudden painless unilateral decrease in vision. The effect of CRVO on final visual acuity varies with the degree of MO, and presence of ischaemia. Presenting visual acuity is typically a good predictor of final visual outcome. Patients who present with visual acuity ≥ 6/12 typically retain good vision whilst 80% of those who present with visual acuity ≤ 6/60 do not improve to better than 6/60. CRVO is a predominantly unilateral disease but presents bilaterally in 5% of cases. The risk of developing RVO in the contralateral eye is about 5% in 12 months.

There are two main subtypes of CRVO- ischaemic and non-ischaemic. Ischaemic CRVO is diagnosed if the total area of angiographic non-perfusion is at least 10 disc areas and it has a poorer prognosis than the non-ischaemic sub-type. Complications of ischaemia include neovascularisation of the retina, optic disc, iris and angle and neovascular glaucoma. Eyes with more than 30 disc areas of ischaemia are more prone to these complications. Approximately 20% present with ischaemic CRVO and 30% of non-ischaemic CRVO can convert to the ischaemic CRVO in 3 years.

CRVO related MO is presumed to occur secondary to retinal hypoxia leading to local vascular endothelial growth factor (VEGF) upregulation, with resultant increased vascular permeability, macula oedema and haemorrhage. Approximately 6,860 people develop CRVO every year in England and Wales of whom 5,150 develop visual impairment and are potentially eligible for treatment (www.NICE.org) (2). Once established, the visual impairment due to CRVO is typically profound with no tendency to improve spontaneously as evidenced in the sham arm of the CRUISE study (3) that showed a mean +0.8 ETDRS letter gain at 6 months and the natural history arm of the Central Retinal Vein Occlusion study (CVOS) (4) that showed no change in mean baseline visual acuity over 3 years. Without intervention permanently impaired visual loss is likely to occur.

In a minority of cases, spontaneous resolution of MO occurs without treatment but it does not typically show a corresponding improvement in visual acuity. Therefore, prompt treatment is advocated. Ranibizumab is a humanized, affinity-matured VEGF antibody fragment that binds to and neutralizes all isoforms of VEGF-A and their biologically active degradation products. Ranibizumab was the first anti-VEGF therapy to demonstrate improved visual outcomes in patients with neovascular age related macular degeneration (nvAMD) and is now approved by the FDA and EMA for MO due to CRVO. This is based on the CRUISE study data (3) that showed monthly intraocular ranibizumab therapy improved mean BCVA by +15 ETDRS letters at 6 months and PRN regimen with monthly monitoring improved mean BCVA by +14 letters at 12 months. In an open label extension (HORIZON) (5) from months 12 to 24, the mean visual acuity (VA) in CRVO patients reduced by 4.1 letters with an average of 3.5 injections in 12 months. Ranibizumab was well

tolerated with 6.5% of patients having some degree of cataract after 2 years and < 1% having any rise in intraocular pressure.

Aflibercept is a fusion protein of the key domains of VEGF receptors 1 and 2 and human IgG Fc that blocks all VEGF-A isoforms and placental growth factor. It is FDA approved for CRVO based on the GALILEO (6) and COPERNICUS (7) studies that showed a mean gain of +16.2 letters BVCA at 12 months with 60% gaining ≥ 15 letters at 12 months. Cataract occurred in < 2% and glaucoma in < 0.58% of patients at 12 months. Cumulative safety data to date does not show an increased risk of any ocular or systemic adverse events with this anti-VEGF agent compared to other similar drugs used for these indications. The known adverse events are low risk of arterial thromboembolic events as defined by the Antiplatelet Trialists' Collaboration (APTC). However, the participants who received aflibercept showed no increased risk of either cardiovascular or cerebrovascular events compared to the control arm in the COPERNICUS and GALILEO studies of MO due to CRVO, and the VIEW studies in neovascular age related macular degeneration (nvAMD). It is contraindicated in pregnancy (Section 10.11)

When given at 8 weekly intervals after a loading phase aflibercept has been demonstrated to be non-inferior to ranibizumab in wet age related macular degeneration. This longer acting property, likely reduced frequency therefore of repeat treatment and potential for improved cost effectiveness has not been explored in a pragmatic trial comparing it to other anti-VEGF blockers, i.e. ranibizumab and bevacizumab. NICE has recommended this drug for MO due to CRVO (TA305).

Bevacizumab is a monoclonal antibody that inhibits vascular endothelial growth factor (VEGF), a mediator in the pathogenesis of common and disabling eye disorders including neovascular age related macular degeneration (nvAMD), diabetic macular oedema (DMO) and retinal vein occlusion (RVO). EMA licensed VEGF inhibitors include ranibizumab and aflibercept for nvAMD, DMO and RVO. Bevacizumab is EMA licensed for the treatment of cancer but not for use in the eye. However, it is of crucial importance to fully assess its suitability for intraocular use because: (i). it is substantially cheaper when divided by a compounding pharmacy into multiple doses from a single 4ml vial , than ranibizumab or aflibercept, (ii) it was found by the Decision Support Unit (DSU) (August 2012) to be commonly used in NHS trusts across the UK for nvARMD, DMO and RVO and other less common indications such as choroidal neovascularisation due to myopia and retinal dystrophies (8), (iii) it is very widely used in UK private practice, (iv) there have been concerns about the possible systemic side effects following intraocular injection of bevacizumab. Two large publicly funded RCT's, the IVAN (9) and CATT (10) studies found an increased risk of hospitalisation related serious adverse events with bevacizumab. In addition, two large retrospective studies have suggested a better safety profile for ranibizumab compared to bevacizumab (11, 12). (v) a small number of indirect comparisons of bevacizumab with other anti-VEGF agents in MO secondary to CRVO are limited by inadequate study size.

To date, bevacizumab has been found to be non-inferior to ranibizumab for all visual acuity primary and secondary endpoints in nvAMD in the IVAN and CATT studies.. In the elderly population, there was no increased risk of arteriothromobotic events (ATCs) including myocardial infarction and cerebrovascular accident as defined by the APTC, attributable to bevacizumab compared to ranibizumab. Although at one year there were more hospitalisations for subjects receiving bevacizumab in both clinical trials, these were for

illnesses unrelated to the known side effects of bevacizumab, were not seen in patients in other studies who received intravenous bevacizumab at much higher doses than would reach the systemic circulation from intravitreal injection and were not seen at two years. No large studies have reported the risks of bevacizumab in younger populations such as those who suffer from DMO and RVO. A small randomised trial (n=80) reported no additional adverse effects of bevacizumab therapy in DMO compared to patients receiving laser therapy (13). The known adverse events of bevacizumab likely include a low risk of arterial thromboembolic events as defined by the APTC were similar to ranibizumab and aflibercept.

From a mechanistic standpoint however, nvAMD is an exudative maculopathy in which there are clinical characteristics including intraretinal accumulation of fluid which are similar to the morphological manifestations of macular oedema secondary to RVO. This and the study findings therefore strongly support the use of bevacizumab in other exudative maculopathies including RVO.

However there is limited evidence regarding its use in central retinal vein occlusion (CRVO) with macular oedema (MO). Sixteen patients with CRVO randomised to bevacizumab versus intravitreal triamcinolone gained a mean of 0.32 logMAR over 9 months with an average of 2.38 injections (14). 18 of 30 (60%) patients treated continuously for 12 months with bevacizumab gained ≥ 15 letters BCVA compared to 10 of 30 (33%) in the sham / bevacizumab group (15). Nevertheless the NICE STA for ranibizumab in RVO requested that the Decision Support Unit undertake additional work so as to consider bevacizumab as a comparator. In addition, the NICE TAG 283: Lucentis (ranibizumab) in RVO and the Eylea (aflibercept) TAG 305 both recommended that further head to head trials including bevacizumab were needed for RVO (2, 16). It was therefore proposed to conduct the LEAVO trial in CRVO to (i) Compare the clinical effectiveness of bevacizumab, aflibercept and ranibizumab in a pragmatic trial over 24 months where patients are followed up over the natural history of the disease (ii). Compare the cost-effectiveness of the agents in a pragmatic trial that closely resembles clinical practice (iii). Describe the safety profile of each agent for ocular and systemic adverse events over 24 months.

3.2 Implications

Since 2012, novel therapies have been approved for NHS use in CRVO with MO, for the first time affording physicians the opportunity to prevent progressive visual loss in patients with this disabling condition. Whilst these therapies are welcome, the ultimate aim must be to determine the most clinically effective and safe agent that can be delivered and is affordable for long term NHS use.

Clinical trials in nvAMD have demonstrated that bevacizumab is as effective and a safe alternative to ranibizumab, delivered at a fraction of the cost (9, 10). It is estimated that the NHS cost saving of switching from ranibizumab to bevacizumab would be approximately £85m per year (17). Sales of ranibizumab in the US in 2011 exceeded £1billion, making it the single most expensive item covered under the Medicare Programme Part B (17) accounting for over 10% of the budget. In the CATT study the bevacizumab drug cost for 2 years of discontinuous therapy was $705 compared to $44,800 for continuous ranibizumab therapy.

Clearly, even the current discounted cost of ranibizumab is not affordable for long term NHS use, especially since the indications for its use now include nvAMD, DMO and RVO.

With the publication of the IVAN two year results confirming the one year findings for efficacy and no new safety concerns pertaining to bevacizumab per se, and assuming compounding pharmacies are able to endorse a quality assured, regular and safe supply of bevacizumab, the opportunity now exists for bevacizumab to be considered as an alternative to ranibizumab for first line treatment of nvAMD.

For CRVO with MO, there is robust clinical trial data for the clinical effectiveness of ranibizumab (3, 5) (CRUISE) and aflibercept (6, 7) (COPERNICUS & GALILEO) and anecdotal reports of the efficacy of bevacizumab. Despite this, no direct comparison between these three agents has been undertaken, nor is one planned, to determine their relative clinical effectiveness, required frequency of administration, side effect profile and cost effectiveness. Furthermore, no alternative anti-VEGF or other novel therapies are expected in to be licensed or NICE approved for CRVO with MO for at least 5 years, meaning a study that aims to answer these questions, will guide NHS therapy for the next 5 to 10 years. Such a trial to compare bevacizumab and aflibercept with ranibizumab is therefore urgently needed and the LEAVO study aims to fill this current knowledge gap.

3.3 Preclinical data and Clinical data

Information on preclinical and clinical studies for Ranibizumab, Aflibercept and Bevacizumab can be found in the current version of the SPC on the eMC website: **http://www.medicines.org.uk/emc/**

3.4 Rationale and risks/benefits

3.4.1 Rationale

MO secondary to CRVO typically causes significant visual loss with no propensity to improve without treatment. Until recently there was no proven effective treatment for this condition. The CRUISE study showed that visual acuity improved by at least 3 lines (15 letters) in 50% of patients at one year with regular intravitreal anti-VEGF ranibizumab therapy. However the treatment is not effective in all patients, needs to be given regularly over at least one year and is expensive. The COPERNICUS and GALILEO studies showed similar visual gains to regular intravitreal aflibercept therapy with a potential need for fewer injections. Small investigator led studies have shown similar benefits in MO due to CRVO with intravitreal anti-VEGF bevacizumab therapy. Although it is unlicensed for intraocular use, it has been employed worldwide, with no greater prevalence of side effects and at a fraction of the cost of ranibizumab and aflibercept. In addition, the longer term outcome of therapy with ranibizumab, aflibercept and bevacizumab is unclear, as is the outcome of treatment in severe cases because patients in this category were typically excluded from the licensing clinical trials. This study will therefore compare the clinical and cost effectiveness of these three anti-VEGF therapies in the treatment of MO secondary to CRVO over the 2 year natural history of the disorder to allow an informed decision regarding the appropriate drug in terms of clinical and cost effectiveness for clinical practice.

3.4.2 Benefits

1. The trial will determine if there are any differences in clinical effectiveness, safety profile and dosing frequency between ranibizumab, aflibercept and bevacizumab in MO due to CRVO in a randomised clinical trial setting.
2. Clearly define the requirement for anti-VEGF therapy in the second year of MO due to CRVO.
3. Provide a detailed cost effectiveness comparison between the investigational agents and the comparator to inform the appropriate agent for use in clinical practice.
4. To follow a treatment schedule in the study which will provide optimal therapy but at the same time be a pragmatic way to manage the patients with MO due to CRVO and which can form the basis of subsequent clinical therapy schedules.

3.5 Risks and Assessment / Management of Risk

1. Risk of bevacizumab

   ii. Manufacture: There is a risk to the supply of bevacizumab as it is outside the normal NHS supply chain. The DSU Report for bevacizumab (8) reported that Moorfields and Liverpool and Broadgreen Pharmacy Manufacturing Units supplied the majority of bevacizumab for clinical use in the UK and that both had the appropriate MHRA 'Specials' license. Since the latter successfully supplied bevacizumab to the IVAN study, for which it conducted additional drug stability work, it has been commissioned to do so for the LEAVO study under its MA (IMP) Licence. Particular attention will be paid to the manufacturing process, supply and storage at study sites. See IMP Section: 9.

   iii. There is no comprehensive data regarding the effect of bevacizumab in pregnancy and it is therefore contraindicated. Females of child bearing age will require a negative urine pregnancy test before enrolment in the study and will be advised to use an effective form of contraception throughout the trial and for 6 months after their last trial injection. Participants will also be reminded to notify their local study team if they fall pregnant during this time. The drug will be stopped immediately if a subject does become pregnant. The pregnancy will be reported using a pregnancy form and followed up until outcome. The collection of study data will continue until the end of the study provided the participant does not withdraw ongoing consent. See Pregnancy Section: 10.11.
2. Risk of aflibercept: intravitreal aflibercept has been widely used globally for retinal vascular diseases and nvAMD, is EMA licensed and NICE recommended for nvAMD and CRVO. Cumulative safety data to date does not show an increased risk of any ocular or systemic adverse events with this anti-VEGF agent compared to other similar drugs used for these indications. There is therefore no risk to the use of this drug over and above standard care.
3. Risk of ranibizumab: intravitreal ranibizumab has been very widely used globally for retinal vascular diseases including DMO, and MO due to CRVO and nvAMD. It is EMA licensed and NICE recommended for nvAMD, DMO and MO due to CVO and the mainstay of therapy worldwide for these conditions. Cumulative safety data to date does not show an increased risk of any ocular or systemic adverse events compared to other similar drugs used for these indications. There is therefore no risk to the use of this

drug over and above standard care.

4. Risk of intravitreal injection: The procedure is the most common surgical procedure done in ophthalmology with minimal adverse events. The reported adverse events are allergy to anaesthetic drops or povidone iodine, subconjunctival haemorrhage, discomfort and pain that last up to 24 hours, transient elevation of intraocular pressure and an inflammatory reaction that typically resolves spontaneously or requires treatment with topical steroids. Serious adverse events are endophthalmitis (intraocular infection) that may occur in 1:2000 injections, retinal detachment (incidence is less than 1%) and vitreous haemorrhage (incidence is less than 1%). However, these risks are no different to standard clinical care and the participants would be treated with intravitreal injections clinically with one of the above agents, even if not enrolled in this study.
5. Risk of ancillary tests: Allergy to topical medications including anaesthetic drops and mydriatic drops. Complications of fundus fluorescein angiography (FFA) are transient nausea and vomiting, yellow discoloration of skin and urine and very rarely allergic reaction. However, these risks are no different to standard clinical care and the participants would undergo these tests clinically even if not enrolled in this study.

4 Objectives

4.1 Trial Objectives

The objective is to compare the relative clinical and cost effectiveness of the anti-VEGF agents bevacizumab (investigational treatment), aflibercept (investigational treatment) and ranibizumab (standard care) in MO due to CRVO over 100 weeks. We want to determine if bevacizumab or afilbercept are as effective as ranibizumab in reducing visual loss from MO due to CRVO, whether they have an equivalent side effect profile and whether either could be considered as a recommended NHS treatment based on non-inferior clinical effectiveness and superior cost-effectiveness.

4.2 Primary Objectives

1. To determine whether bevacizumab is non-inferior to ranibizumab in treating visual loss due to MO secondary to central retinal vein occlusion at 100 weeks
2. To determine whether aflibercept is non-inferior to ranibizumab in treating visual loss due to MO secondary to central retinal vein occlusion at 100 weeks.

4.3 Secondary Objectives

1. To determine the difference between arms in mean change in best corrected visual acuity at 52 weeks.

2. To determine the difference between arms in the proportion of participants with ≥ 15 ETDRS letter improvement (appreciable visual gain), ≥ 10 letter improvement, <15 letter loss and ≥ 30 ETDRS letter loss (severe visual loss) at 52 and 100 weeks.
3. To determine the difference between arms in the proportion of participants with ≥73 ETDRS letters or better than 6/12 Snellen equivalent (ie approximate driving visual acuity), ≤58 ETDRS letters (≤6/24) and ≤ 19 letters (≤3/60)(CVI partial and severe visual impairment) at 52 and 100 weeks.
4. To determine the difference between arms in the mean change in OCT CST and macular volume at 52 and 100 weeks.
5. To determine the difference between arms in the proportion of participants with OCT CST < 320µm (Spectralis or refer to appendix 1) at 52 and 100 weeks (key guide to subsequent NHS clinical practice).
6. To determine the differences between arms in the mean number of injections performed per study eye per participant at 100 weeks.
7. To determine any differences in the relative effectiveness of the investigational treatments and comparator on quality of life and resource utilization, reported as Incremental Cost Effectiveness Ratios (ICERs) at 52 weeks.
8. To determine any differences in the relative effectiveness of the investigational treatments and comparator on quality of life and resource utilisation, reported as Incremental Cost Effectiveness Ratios (ICERs) at 100 weeks.
9. To detect any differences in the prevalence of local and systemic side effects at 100 weeks
10. To determine differences between arms at 100 weeks in the proportion i. of persistent non-responders (see Section 8.14.7) ii. of participants that develop a change in retinal non-perfusion compared to screening iii. of participants that develop anterior and posterior segment neovascularisation.
11. To determine differences between arms in mean change in best corrected visual acuity at 100 weeks due to i) baseline visual acuity stratified as ≤38 letters, 39-58 letters, 59-78 letters, ii) duration of disease stratified as: <3 months, 3-6 months and > 6 months, iii) treatment stratified as naïve vs previous treatment iv) quantity of retinal ischaemia (<10, ≥10 and < 30, and ≥ 30 DA of non-perfusion).
12. To determine differences between arms in changes in area of non-perfusion at 100 weeks and OCT anatomical features over time and at 100 weeks

5 Trial design

5.1 Overall design

This is a phase III randomised controlled double-masked non-inferiority clinical trial to evaluate the relative clinical and cost-effectiveness of intravitreal bevacizumab and aflibercept compared to ranibizumab in MO due to CRVO. 459 patients with MO due to CRVO in at least one eye will be randomised 1:1:1 to bevacizumab [1.25mg in 50ul] (Royal Liverpool) and aflibercept [2.0mg/50ul] and ranibizumab [0.5mg/50ul] all administered by intravitreal injection and followed for 100 weeks. The study will be conducted across approximately 40 Ophthalmology centres in the UK with expertise in retinal disorders and a proven track record in effectiveness research. The primary outcome will be the difference in mean ETDRS BCVA (best corrected visual acuity) letter score at 100 weeks. Secondary outcomes will include additional BCVA outcomes, differences in OCT

central macular thickness, change from baseline in visual function questionnaire, VFQ-25, use of resources and adverse events. The primary outcome of cost effectiveness analysis will be mean incremental cost per QALY.

After participant study eligibility has been confirmed, the date of the milestone visits at weeks 0, 12, 24, 52, 76 and 100 weeks will be calculated and agreed. Visits at weeks 4 and 8 will also be fixed to ensure that the patient is able to attend these treatment visits. Following confirmation that the participant is able to attend these visits, randomisation can occur.

From this point forward, all intervening follow up visits after week 12 will be flexible and designed to fit around the milestone visits. Since intravitreal injection is not recommended less than 4 weeks after the previous injection, it is likely that there will be 'slippage' of the study visit schedule. This is acceptable and it may be necessary to omit a scheduled 4 or 8 weekly follow-up visit if the next scheduled clinical visit falls immediately before a milestone visit (see examples below):

If for instance, at the week 44 visit the participant has had 2 prior visits which were one week late but still within window, then the 44 week visit would effectively be at 46 weeks. The local site PI could then arrange for the 52 week visit to be six weeks later and still remain within the agreed window and the 48 week visit would not be scheduled. If for instance at the 32 week visit a participant had 'slipped' two weeks and actually attended at week 34, the week 36 visit could be cancelled and the patient could be scheduled at week 38 i.e. the 40 week visit is brought forward two weeks.

In the context of a non-inferiority study, the protocol is designed to be as flexible as possible to accommodate variations in normal clinical practice between individual investigators where possible, following mandated injections at weeks 0, 4, 8 and 12. The protocol thus provides guidance on recommended treatment frequency but deviation from this schedule by utilising the wide visit windows and omitting treatment visits where visit 'slippage' has occurred, is permissible and not considered a protocol deviation.

After mandated administration in all arms at baseline, 4, 8, and 12 weeks, further PRN intervention if retreatment criteria (Section 8.14.2) are met will be administered at weeks 16 and 20.

From week 24 to week 96, intervals will initially be 4 weekly (with a -14 to + 14 day visit window) with the potential to increase to 8 weekly (with a -14 to + 14 day visit window) if criteria for 'stability' are achieved. 'Stability' is defined as three successive visits from week 16 onwards at which retreatment criteria are not met (Section 8.14.3) and so the first time at which treatment could be deferred for 8 weeks is week 24.

Similarly 'Success' is defined as an ETDRS letter score > 83 letters and if present at any retreatment visit then treatment should not be given at that point and the patient reviewed at either 4 or 8 weeks depending on their pre-existing visit schedule. The > 83 letter ETDRS letter score criteria for further study participation is illustrated in Section 8.14.4.

At each visit between weeks 24 and 96 inclusively, temporary discontinuation criteria maybe met (See Section 8.14.5). If so, the PI or his designee at their discretion can withhold treatment to prevent therapy in a participant who has not responded to at least their last three injections.

If retreatment criteria are met at an 8 weekly or unscheduled visit, then 4 weekly visits will be resumed until retreatment criteria are not met again on three occasions and an 8 weekly visit is re-established. If a patient achieves the criteria for success or temporary discontinuation then treatment will be discontinued until retreatment criteria are met again.

Temporary deferral of treatment is allowable in certain circumstances eg. vitreous haemorrhage (see Section 0) but the participant would still be asked to attend the key study research visits.

For the Interventions and Comparators see Sections 9.1 and 9.2

For Primary and Secondary outcomes see Section 13.1.1 and 0.

6 Selection of Participants

6.1 Inclusion Criteria

1. Subjects of either sex aged ≥ 18 years.
2. Clinical diagnosis of centre-involving macular oedema (MO) due to CRVO
3. CRVO of ≤ 12 months duration.
4. Best corrected visual acuity in the study eye ≥ 19 and ≤ 78 ETDRS letters (approximate Snellen VA 3/60 to VA 6/9).
5. Best corrected visual acuity in the non-study eye ≥ 14 ETDRS letters (approximate Snellen VA ≥ 2/60).
6. SD-OCT central subfield thickness (CST) > 320µm (Spectralis) predominantly due to MO secondary to CRVO in the study eye. See appendix 1 for equivalent CST value for alternative SD-OCT machines.
7. Media clarity, pupillary dilatation and subject cooperation sufficient for adequate fundus imaging of the study eye.
8. In cases of bilateral CRVO, if both eyes are potentially eligible, unless the patient prefers otherwise the worst seeing eye will be recruited.

6.2 Exclusion Criteria

The following apply to the study eye only and to the non-study eye only where specifically stated:

1. Macular oedema considered to be due to a cause other than CRVO (e.g. diabetic macular oedema, Irvine-Gass syndrome).
2. An ocular condition is present that, in the opinion of the investigator, might affect macular oedema or alter visual acuity during the course of the study (e.g. vitreomacular traction)
3. Any diabetic retinopathy or diabetic macular oedema at baseline clinical examination of the study eye.
4. Moderate or severe non proliferative diabetic retinopathy (NPDR) or quiescent, treated or active proliferative diabetic retinopathy (PDR) or macular oedema in the non-study eye. Note: Mild NPDR only is permissible in the non-study eye.
5. History of treatment for MO due to CRVO in the past 90 days with intravitreal or peribulbar corticosteroids or in the last 60 days with anti-VEGF drugs or >6 prior anti-VEGF treatments in the previous 12 months.
6. Active iris or angle neovascularisation, neovascular glaucoma, untreated NVD, NVE and vitreous haemorrhage or treatment for these conditions in the last 1 month.
7. Uncontrolled glaucoma [>30mmHg], either untreated or on anti-glaucoma medication at screening.
8. Any active periocular or intraocular infection or inflammation (e.g. conjunctivitis, keratitis, scleritis, uveitis, endophthalmitis).

Systemic exclusion criteria:

9. Uncontrolled blood pressure defined as a systolic value > 170mmHg and diastolic value > 110mmHg.
10. Myocardial infarction, stroke, transient ischaemic attack, acute congestive cardiac failure or any acute coronary event < 3 months before randomisation
11. Women of child bearing potential unless using effective methods of contraception throughout the study and for 6 months after their last injection for the trial. Effective contraception is defined as one of the following:
    a. Barrier method: condoms or occlusive cap with spermicides.
    b. True abstinence: When it is in line with the preferred and usual lifestyle of the subject. Periodic abstinence (e.g. calendar, ovulation, symptothermal, post-ovulation methods) and withdrawal are not acceptable methods of contraception.
    c. Have had tubal ligation or bilateral oophorectomy (with or without hysterectomy).
    d. Male partner sterilisation. The vasectomised male partner should be the only partner for the female participant.
    e. Use of established oral, injected or implanted hormonal methods of contraception and intrauterine device
12. Pregnant or lactating women.
13. Males who do not agree to an effective form of contraception for the duration of the study and for 6 months after their last injection for the trial.
14. Hypersensitivity to the active ingredients aflibercept, bevacizumab or ranibizumab or any of the excipients of these drugs.
15. Hypersensitivity to Chinese Hamster Ovary (CHO) cell products or other recombinant human or humanised antibodies
16. A condition that, in the opinion of the investigator, would preclude participation in the study.
17. Participation in an investigational trial involving an investigational medicinal product within 90 days of randomisation

6.3 Re-screening of patients

1. Patients that do not meet the BCVA or OCT CST inclusion criteria may be rescreened a minimum of 4 weeks after their last screening visit if they are thought to meet the eligibility criteria.
2. Individuals that do not meet other modifiable inclusion criteria, e.g. blood pressure, may be re-screened a minimum of 2 weeks after the last screening visit.

All assessments performed at the initial screening visit should be repeated during the rescreening visit except fluorescein angiography, if the rescreening visit is within 10 weeks of the original screening visit, otherwise this too should be repeated. If a patient is found to be eligible on re-screening and is randomised,

their initial entry on the eCRF system should be updated rather than creating a ‘new’ patient on the system. This will avoid ‘double counting the patients in the CONSORT diagram.’

7 Recruitment

The study will recruit from approximately 40 centres over an 18 month recruitment period. Recruitment will be competitive; however each site will be allocated a minimum target number of patients to recruit. Sites will be set up strategically to ensure the recruitment period is fully utilised. Patient Identification Centres may be set up to maximise and boost recruitment. Eligible patients will be invited to participate via their local clinics, or via an invitation letter. Eligible participants can either respond directly to the recruiting centre (with notification to their doctor) or the local PIC site via phone or response slip

Within each site patients will be identified from subspecialty retina clinics, general clinics, and eye casualty clinics and at which clinical examination and discussion of a study will be undertaken and the PIS provided.

8 Study procedures and schedule of assessments

8.1 Informed consent procedure

The Principal Investigator or designated sub-investigator will be responsible for ensuring that a patient is fully consented following adequate explanation of the aims, methods, anticipated benefits and potential hazards of the study. Patients will be advised that any data collected will be held and used in accordance with the Data Protection Act 1998. Patients will be given at least 24 hours after receiving the patient information sheet (PIS) to consider taking part. The PI or designee will record in the medical notes date the patient information sheet was given to the patient. The PI or designee will explain that patients are under no obligation to enter the trial and that they can withdraw at any time, without giving a reason. No clinical trial procedures will be conducted prior to taking consent from the participant and consent will not denote enrolment into the trial. A copy of the signed informed consent form will be given to the patient. The original signed form will be retained at the study site and a copy placed in the medical notes. If new safety information results in significant changes in the risk/benefit assessment, the patient information sheet will be reviewed and updated if necessary and subjects will be re-consented as appropriate.

8.2 Randomisation procedures

A patient identification number (PIN) will be generated by registering the patient on the MACRO eCRF system (InferMed Macro), after consent has been signed. This unique PIN will be recorded on all source data worksheets and used to identify the patient throughout the study. Randomisation will be via a bespoke web based randomisation system hosted at the KCTU. Authorised site staff will be allocated a username and password for the randomisation system by the Trial Manager. An authorised staff member who will typically be the PI or designee will log into the randomisation system (www.ctu.co.uk and click ‘randomisation – advanced’ and select LEAVO) and enter the patients details, including unique PIN.

Once a patient is randomised, the system will automatically generate emails to key staff within the study. Unmasked e-mails sent to site pharmacies will alert them to a patient's treatment arm: ranibizumab, aflibercept or bevacizumab. The pharmacy department will use the email to cross check the trial prescription to ensure that the correct medication is being dispensed for the correct patient. Additional masked emails will be generated from the randomisation system to key trial site staff, and unmasked e-mails to the emergency unmasking service (eSMS Global) and unmasked trial management staff.

8.3 Masking

Masking of treatment allocation: the randomization process will inform only the pharmacy at the local trial site of the subjects' treatment allocation, with a copy to the emergency unmasking service (eSMS Global) and unmasked trial management staff.

The study drug the patient will receive will be transferred in a masking bag to the dedicated injection room. Prior to leaving the Pharmacy a unique seal will be attached to the bag. The non-transparent masking bag, designed to securely and safely transport medication, will have a safe zipped compartment containing a pre-printed form detailing the participants unique PIN, date of birth, date drug dispensed and injection batch number. Prior to the participant entering the injection room, the unmasked experienced injector will break the seal, take the drug out of the masking bag. In the case of bevacizumab, this will be in a prefilled syringe but ranibizumab and aflibercept are currently provided in a vial and will be drawn into a syringe, by the unmasked injector. The syringe will then be placed on the injection trolley, out of view of the patient, who will then be invited into the room, to lie on the injection bed and the injection administered to the patient. Ranibizumab and Aflibercept may be provided in a unique prefilled syringe by the manufacturer during the course of the trial and vials cease to be available. In this situation, the unmasked injector will take care not to allow the subject sight of the syringe either before or after the injection has been given. This will be done by performing the injection with the patient lying down and the injection given via the pars plana in any quadrant of the eye with the syringe being brought to and taken away from the injection site from the patients inferotemporal field of vision so that it is not passed across their line of sight. The unmasked injector will sign the source notes to the effect that the treatment in the masked bag has been administered to the patient, without specifying the treatment, and will also sign the pre-printed form within the masking bag to the effect that the drug detailed on the form has been given to specified participant. The empty drug syringe with needle and vial will be disposed of in the injection room. The masking bag and completed pre-printed will be returned to pharmacy, for drug accountability purposes (See Section 9.8). The drug outer packaging will be disposed of in the injection room.

The clinical assessment team including the site PI, optometrist i.e. assessor of the primary outcome, site trial co-ordinator, the clinical investigator, clinical assessment study nurse and ophthalmic technician will therefore remain masked throughout the study as there will be no record of the subjects' treatment arm in the source notes or case report form. Similarly, co-ordinators or administrators completing questionnaires in person with participants or in extreme circumstances only by telephone at specific time points will have details of subject study number only. If at any time, information regarding treatment allocation is shared with the outcome assessors, then this must be recorded in the Trial Master File, the person (s) involved will meet with the site PI

to ensure no repetition occurs and undertake not to convey this information either to the participant or others involved in the project. Certain secondary outcomes e.g. interpretation of fluorescein angiography will occur at the remote NetwORC UK Reading Centre where the assessors will be masked as to the treatment allocation. These masking procedures will avoid both performance and detection bias. We will describe the completeness of outcome data for each outcome, including any unmasking in error, reasons for attrition and exclusions from the analysis.

8.4 Unmasking and emergency unmasking

This is a double masked study and both the patient and the investigator team, with the exception of the unmasked injector as outlined in Section 8.3, will be masked to the treatment allocation. Cases that are considered SUSARs will be unmasked to the Chief Investigator prior to reporting to the Sponsor, MHRA and main REC. All investigators will be informed of all SUSARs occurring throughout the study on a case-by-case basis. This will not include information on trial arm in order to avoid the risk of inadvertently unmasking investigators. In cases of SUSARs and SARs requiring immediate management of the participant the unmasked Trial Manager will be informed.

All participants will be provided with an emergency code break card providing details of the 24 hour emergency code break service undertaken by Guy’s Medical Toxicology Unit (eSMS Global). If a request for code break is received from a physician (e.g. the patient’s general practitioner) outside the research team, eSMS will attempt to contact the research team to verify the request before the code is broken.

If the code is broken, because it is deemed necessary for the immediate management of the participant, details including patient study number, the date code break was performed, the person who broke the code, and reason for code break shall be recorded by the emergency code break service and retained. The Trial Manager will be informed of the unmasking event. If clinically indicated, the participant will be withdrawn from study medication.

Accidental unmasking will be dealt with on a case by case basis, if and when they arise. The patient’s data should continue to be collected according to the study assessment schedule, even in the event of unmasking or withdrawal from study medication, unless the patient refuses.

8.5 Screening assessment

The patient must have received the Patient Information Sheet not less than 24 hours before the screening assessment. The screening and baseline visits can be performed on the same day provided all test results are available. All participants will be consented prior to any study specific procedures being carried out. Please see section 8.13 for Flow chart of study assessments performed at the screening visit.

8.6 Baseline assessment

The baseline visit and patient randomisation must be performed no later than 10 days after the screening visit. Please see section 8.13 for Flow chart of study assessments performed at the baseline visit

8.7 Subsequent assessments

Please see section 8.13 for Flow chart of study assessments

8.8 Visit window for study appointments

When the patient attends for their screening visit, the baseline visit should occur within 10 days of this (it can be the same day). The baseline visit (that is, date of randomisation) becomes time-point zero from which all other visit dates are calculated. Sites should aim to keep weeks 4, 8 and 12 within 0 to +14 days of the calculated visit day and all other visits +/- 14 days of the calculated visit day.

a. If a non-milestone visit falls out of window, the participant should be brought in under their next scheduled visit. If a milestone visit falls out of window, any milestone assessments should be performed at their next scheduled visit.
b. The dosing interval between two doses of ranibizumab, aflibercept or bevacizumab cannot be shorter than four weeks.

A within window flexibility to complete the assessments and treatment is permitted.

8.9 Study assessments and methods

8.9.1 Participant demographics, medical and ophthalmic history

This information can be retrieved from the participant, hospital medical records or general practitioner. Data will include age, gender and ethnic background. Data will also be collected on clinically relevant medical history and its management in the last 24 months, and on any prior ocular history and treatment.

8.9.2 Visual acuity tests

Visual acuity tests are performed using the validated ETDRS vision charts using standard operating procedures. Refracted visual acuity will be done in both eyes at screening, weeks 12, 24, 52, 76 and 100 and at the point of withdrawal. For all other visits, the visual acuity will be tested with the previous most recent protocol refraction. Please refer to the Manual of Operations. Visual acuity examiners will be masked of the treatment. The worksheets used for the visual acuity tests which will detail previous protocol refractions should be retained in a file held with the Principal Investigators team. The visual acuity score will be recorded in the eCRF.

8.9.3 Standard ophthalmic examination

A standard ophthalmic examination using slit lamp biomicroscopy including undilated exam for NVI, RAPD and tonometry should be done in both eyes at all visits. Dilated fundus examination should be performed in both eyes at all milestone visits (screening, baseline, weeks 12, 24, 52, 76 and 100 and at the point of withdrawal). At all other visits, dilated fundus examination should be performed in the study eye and at the discretion of the investigator in the non-study eye. Gonioscopy is indicated prior to dilatation at any visit if NVA, NVI or NVG is suspected.

8.9.4 Spectral Domain Optical Coherence Tomography (SD- OCT)

The central sub-field thickness and total macular volume in both eyes will be recorded from the SD-OCT thickness map at every visit (except baseline), and if applicable, at the point of withdrawal. Any SD-OCT machine may be used for the study but the same model of SD-OCT should be used for each individual throughout the period of the study. SD-OCTs will be transferred to and read by masked graders at the Independent Reading Centres in NetwORC UK. SD-OCTs will be transferred to and read by masked graders at the Independent Reading Centres in NetwORC UK at screening, weeks 52 and 100 only.

8.9.5 Colour Fundus Photography (CFP)

Non stereo, 7-field conventional or wide-angle CFP will be performed at screening, week 52 and week 100 in the study eye. A single posterior pole colour image of the non-study eye will be performed at these visits. CFP will confirm the diagnosis of CRVO and assist interpretation of features identified on fundus fluorescein angiography e.g. to differentiate between non-perfusion and masking due to haemorrhage. If applicable, CFP will be performed at the point of withdrawal, and at any other study visit as per investigator discretion. CFP will be transferred to and read by masked graders at the Independent Reading Centres in NetwORC UK. Please see Manual of Operations for details. Any colour camera capable of taking 7-field CFP or wide angle system may be used but the same model of camera should be used for each individual throughout the period of the study.

8.9.6 Fundus fluorescein angiography (FFA)

Non stereo, 7-field conventional or wide angle FFA will be performed at screening and week 100 in the study eye. A single posterior pole late frame of the non-study eye will be performed at these visits. FFA will quantify the degree of retinal ischaemia and confirm the presence of retinal neovascularisation. If applicable, FFA will be performed at the point of withdrawal, and at any other study visit as per investigator discretion. FFA will be transferred to and read by masked graders at the Independent Reading Centres in NetwORC UK. Please see Manual of Operations for details. Any fluorescein angiography system capable of taking 7-field FFA or wide angle system may be used but the same system should be used for each individual throughout the period of the study.

8.9.7 Questionnaires

The following quality of life and resource use questionnaires will be administered at baseline, 12, 24, 52, 76, 100 weeks and at the point of withdrawal: VFQ-25, EQ-5D with and without vision ‘bolt-on’ and CSRI.

8.9.8 Independent Reading Centres in NetwORC UK

The NetwORC UK will provide each site with a study imaging protocol, incorporated into the Manual of Operations giving instructions and guidance on how to acquire and transfer SD-OCTs, CFPs and FFAs to the Independent Reading Centres. The images will be anonymised to study PIN, and will include the time-point at which the image was collected. The images should be transferred via CD, SFTP or another suitably secure media agreed by the Independent Reading Centre and the Chief Investigator. The images will be accompanied by a transmittal log which will require the patient’s date of birth as an identifier. Sites must ensure that all PINs and dates on images, compliment the information recorded on the transmittal log and that all images are captured, exported and submitted in accordance with the requirements of the study imaging protocols in the Manual of Operations. Sites should aim to transfer the images to the Independent Reading Centres within 2 weeks of capture. The Independent Reading Centres will report to NetwORC UK who will send reports regularly to KCTU for the duration of the study detailing what has been received and what is currently outstanding from each site. NetwORC UK will evaluate study images and report to the study statistician as necessary.

8.9.9 Treatment allocation guess form

Participants and masked optometrists will be asked to complete a treatment allocation guess form at week 100 or at the point of withdrawal to assess how well participant and assessor masking worked for the study.

8.9.10 Study Milestone Assessments

Study milestone assessments, at which key research data is collected, occur at baseline (Week 0) and weeks 12, 24, 52, 76 and 100. These visits, as well as treatment visits at weeks 4 and 8 will be calculated and agreed with the participant prior to randomisation (with flexibility of 0 to +14 days for weeks 4, 8 and 12, and -14 to +14 days for weeks 24, 52, 76 and 100 from the date of randomisation (rather than the date of the previous visit). It is mandatory for all participants to attend all milestone visits, even if a milestone visit falls less than 4 weeks after a treatment visit or if the participant is following an 8 weekly follow up schedule and the next milestone visit falls within the 8 week interval. In practice the schedule is designed to be sufficiently flexible (-14 to + 14 days for all visits after week 12) that this should be avoidable in all but exceptional circumstances. The intervening study treatment visits are deliberately flexible to allow normal clinical practice treatment follow up to be accommodated. All data from the study milestone visits will be entered into the eCRF. For regular treatment visits only BCVA, OCT CST, whether an injection was given, and if no injection was given the reason it was not given will be entered into the eCRF. The latter will be entered onto a single

source data worksheet (SDW) entitled 'Study treatment visit BCVA, OCT CST assessments and injection record' and this will be completed at every participant assessment.

8.10 Definition of end of trial

Patients will be in the trial for approximately 100 weeks from the point of randomisation. End of trial will be defined as the last participant's last study visit.

8.11 Discontinuation criteria

The study may be prematurely discontinued on the basis of new safety information, or for other reasons given by the Data Monitoring and Ethics Committee (DMEC) and/or Trial Steering Committee, Sponsor, regulatory authority or Research Ethics Committee concerned.

8.12 Stopping rules

All data reviewed by the DMEC will determine safety issues. The trial can stop at any time if there are significant safety issues. All serious adverse reactions will be reported to the KCTU within 24 hours of learning of its occurrence.

8.13 Flowchart of study assessments

^Mandatory Visits: Loading (wk 4 & 8) & Milestones (baseline, wks 12, 24, 52, 76, 100)	Screening	Baseline	Week 4	Week 8	Week 12	Week 16	Week 20	Week 24		Week 52		Week 76		** Week 100	Unsch. Visit.	** Withdrawal Visit
Variable treatment visits									4-8 weekly		4-8 weekly		4-8 weekly			
Weeks		0	4	8	12	16	20	24	28-48	52	56-72	76	80-96	100	1-99	13-97
Visit window (days)	-10 to 0	0	0 to +14	0 to +14	0 to +14	-14 to +14	-14 to +14	-14 to +14	-14 to +14	-14 to +14	-14 to +14	-14 to +14	-14 to +14	-14 to +14		
Informed Consent	X															
Inclusion/Exclusion Criteria review	X	X[3]														
Randomisation[1]		X														
Urine Pregnancy test in women of child bearing age.	X															
Patient demographics, medical and ophthalmic history	X															
Adverse events	X	X	X	X	X	X	X	X	X	X	X	X	X	X	X	X
Concomitant medication review	X	X	X	X	X	X	X	X	X	X	X	X	X	X	X	X
Blood Pressure	X	X	X	X	X	X	X	X	X	X	X	X	X	X	X	X
Best corrected ETDRS visual acuity in both eyes (refraction visit =X1)	X1	X	X	X	X1	X	X	X1	X	X1	X	X1	X	X1	X / X1[5]	X1
Standard Ophthalmic Examination	X	X	X	X	X	X	X	X	X	X	X	X	X	X	X	X
SD-OCT in both eyes	X		X	X	X	X	X	X	X	X	X	X	X	X	X	X
7-field or wide-angle colour fundus photography[2]	X									X				X	+/-[5]	X
7-field or wide angle fundus fluorescein angiography[2]	X													x	+/-[5]	X
VFQ-25 and EQ-5D with and without vision 'bolt-on'		X			X			X		X		X		X	+/-[5]	X
Resource Use Questionnaire (CSRI)		X			X			X		X		X		X	+/-[5]	X
Treatment Allocation Guess Form[4]														X		X
Administer IMP*		X	X	X	X	X2	X2	X2	X2	X2	X2	X2	X2		X2	

X1 – Same day refracted best corrected visual acuity
X2 - PRN treatment.
Study Treatment Visit: non shaded square.
Study Milestone Visit: shaded square
^Milestone visits and mandated loading visit dates should be agreed with participant prior to performing randomisation
*Intravitreal injections including immediate post injection checks are performed as per each trial sites local policy and may include a check of ON perfusion or VA or IOP or a combination of these.
** Participants should be reminded to use an effective form of contraception for 6 months after their last trial injection. Females of child bearing potential should be reminded to notify the local study team if they fall pregnant during this time.
[1]Randomisation should only occur once all other assessments at baseline (week 0) have occurred
[2]Further colour fundus photographs and fluorescein angiography may be performed as per investigator discretion. Colour fundus photographs should be done if a patient converts from non-ischaemic to ischaemic CRVO.
[3]To include review of screening assessment test results and confirmation of eligibility
[4]To be completed by participants and masked site optometrists.
[5]To be performed (as required) if unscheduled visit is a milestone visit.

8.14 Treatment procedures

8.14.1 Treatment schedule

After mandated administration in all three study arms at baseline, 4, 8, and 12 weeks, further PRN intervention will be administered at weeks 16 and 20 if retreatment criteria (Section 8.14.2) are met and VA ≤ 83 letters.

Table 8.14.1.a: Re-treatment Criteria and follow-up schedule at week 16 and 20:

Current visit	Injection	Follow-up
Re-treatment criteria – met and VA ≤ 83 letters	Injection	4 weeks
Re-treatment criteria – not met or VA > 83 letters	No injection	4 weeks

From week 24 to week 96, intervals will initially be 4 weekly (with a -14 to + 14 day visit window) with the potential to increase to 8 weekly (with a -14 to + 14 day visit window) if criteria for ‘Stability’ (Section 8.14.3) are achieved. ‘Stability’ is defined as three successive visits from week 16 onwards at which Retreatment Criteria (Section 8.14.2) are not met and so the first time at which treatment could be deferred for 8 weeks is week 24.

Similarly ‘Success’ (Section 8.14.4) is defined as an ETDRS letter score > 83 letters and if present at any retreatment visit from 16 weeks onwards, then treatment should not be given at that visit and the participant reviewed in 4 weeks if ‘success’ is fulfilled at week 16 or 20 weeks and either at 4 or 8 weeks at any other time point depending on their pre-existing visit interval. If at any subsequent visit, Retreatment Criteria are met and BCVA ≤ 83 ETDRS letters then retreatment is commenced. At each visit between weeks 24 and 96 inclusively, ‘Non responder treatment suspension’ criteria maybe met (Section 8.14.5). If so, the PI or his designee at their discretion can suspend treatment to prevent therapy in a participant who has not responded to at least their last three injections. If the criteria for restarting therapy after ‘Non-responder treatment’ suspension (Section 8.14.6) are met, then the participant should resume therapy.

If Retreatment Criteria are met at an 8 weekly or unscheduled visit, then 4 weekly visits will be resumed. Treatment may be ‘Deferred’ in certain circumstances (Section 0) but the participant would still be asked to attend the milestone visits.

Table 8.14.1.b: Retreatment Algorithm for Weeks 24 to 96:

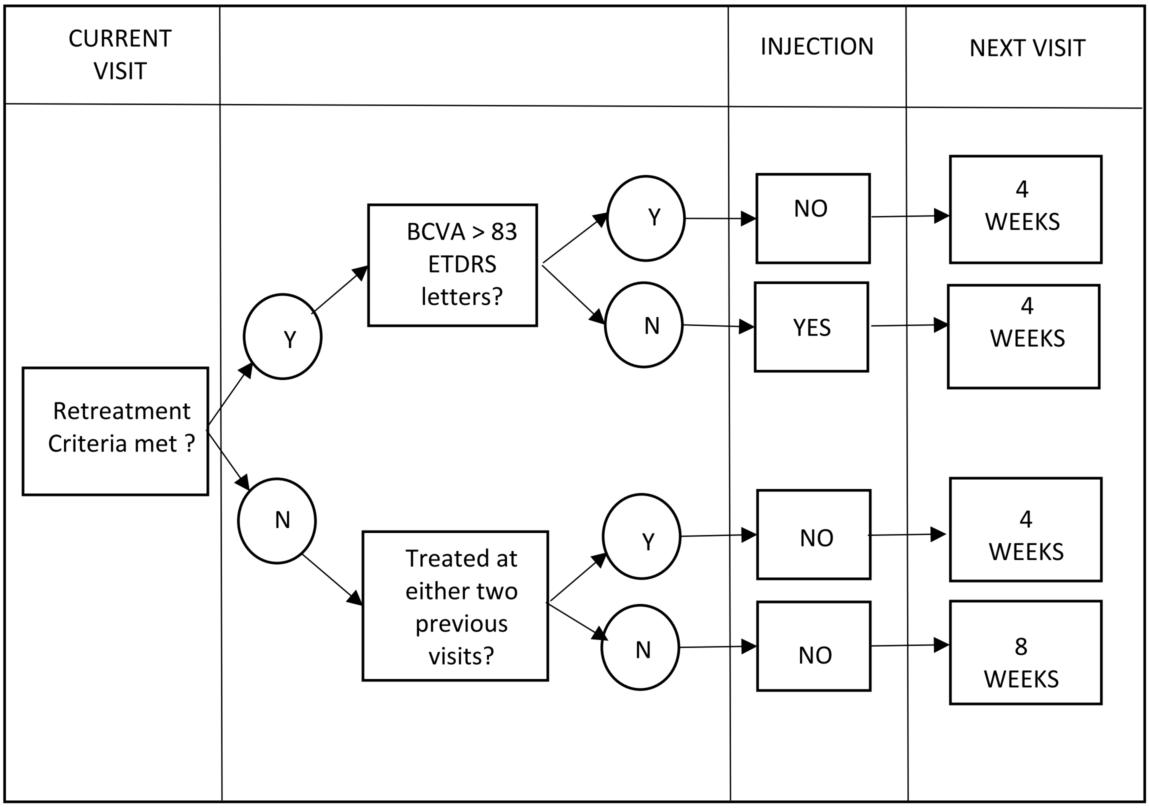

8.14.2 Re-treatment criteria: criteria are met if one or more of the following is present:

1. a decrease in visual acuity of ≥ 6 letters between the current and most recent visit attributed to an increase in OCT CST OR
2. an increase in visual acuity of ≥ 6 letters between the current and most recent visit OR
3. OCT CST >320µm (Spectralis or refer to appendix 1) due to intraretinal or subretinal fluid OR
4. OCT CST increase > 50µm from the lowest previous measurement.

8.14.3 Criteria for 'Stability'

From week 16 onwards, three successive visits in which retreatment criteria are not met fulfil the criteria for 'Stability' and no injection is given at the current visit and the patient is reviewed in 8 weeks (-14 days to +14 day window). This criteria for 'Stability' is incorporated into the week 24 to 96 Retreatment Algorithm, Table 8.14.1.b and is accounted for when the algorithm is followed at each study visit.

8.14.4 Criteria for ‘Success’

At any visit from week 16 onwards if VA > 83 letters (BCVA ≥ 6/6) injection is withheld. This criteria for ‘Success’ is incorporated into the week 24 to 96 Retreatment Algorithm, Table 8.14.1.b and is accounted for when the algorithm is followed at each study visit.

8.14.5 Criteria for ‘Non-responder Treatment Suspension’

From week 24 onwards, treatment can be suspended at any visit if the participant received an injection at the previous three visits and:

1. CST has not decreased by 50um compared to the highest value of CST in the previous 3 visits AND
2. Visual acuity has increased or decreased ≤5 letters from the previous visit

Treatment is deferred and the participant reviewed in 8 weeks. The criteria for ‘Non-responder Treatment Suspension’ are NOT incorporated into the week 24 to 96 Retreatment Algorithm, Table 8.14.1.b. They are at the Investigators discretion and should be considered at each visit

8.14.6 Criteria for restarting therapy after ‘Non-responder Treatment Suspension’

Either of the following is a criteria for restarting therapy:

1. an increase or decrease in BCVA ≥ 6 letters between the current and any visit at or after the point of treatment suspension OR
2. an increase or decrease >50um on OCT CST between the current and any visit at or after the point of treatment suspension.

   The criteria for restarting therapy after ‘Non-responder Treatment Suspension’’ are NOT incorporated into the week 24 to 96 Retreatment Algorithm, Table 8.14.1.b. They are at the Investigators discretion and should be considered at each visit.

8.14.7 Criteria for ‘Persistent Non-responder’

A ‘persistent non-responder’ is defined as a participant who experiences ≤ 5 letter improvement in visual acuity AND < 50um reduction in OCT CST compared to baseline at any assessment in the study at or after 24 weeks.

8.14.8 Temporary Deferral of Treatment

Treatment may be deferred in the following situations:

1. If an eye has experienced adverse effects from prior intravitreal injection, further retreatment with intravitreal agent is at the discretion of the investigator.
2. Treatment with anti-VEGF may be deferred in cases of total vitreous haemorrhage with no clear view of the fundus until the fundus can be sufficiently well visualised to permit subsequent intraocular injection.

3. Anti-VEGF injection may be deferred in an eye that has developed a rhegmatogenous retinal detachment or requires surgical intervention for any reason e.g. tractional retinal detachment threatening the fovea. Anti-VEGF injections may be resumed following surgical intervention.
4. Anti-VEGF injections should be deferred if the interval between the current and previous visit is less than 4 weeks.
5. Anti-VEGF injection may be deferred in a visit where IOP remains above 30mmHg prior to injection despite the use of iopidine or other appropriate topical anti-glaucoma therapy immediately prior to the procedure. The participant may then be prescribed iopidine or other appropriate topical anti-glaucoma therapy for a week and rescheduled for anti-VEGF injection within a week if IOP is reduced to <30 mmHg. Even if this visit falls outside the visit window it will still be considered part of the same visit. At all other times, participants with elevated IOP will be managed with anti-glaucoma therapy at the discretion of the investigator that would reflect their normal clinical practice or according to local site policy.

8.14.9 Unscheduled visits

If at any time, a participant experiences a significant change in visual acuity or new ocular symptoms in the study eye, then he should contact his Study Site. If the change is related to MO secondary to CRVO and an unscheduled visit is required, the injection and follow-up of the patient is based on table 8.14.9a below.

Previous visit (s)	Current visit	Injection	Follow-up
Injected at least 4 weeks ago	Re-treatment criteria met	Injection	Follow-up at either 1. next scheduled visit with creation of a 4 week interval by deferring the visit 0 to + 14 days OR 2. the next but one scheduled visit with creation of a 4 to 6 week interval by foreshortening the visit by 0 to -14 days. Option 2 is only possible if the resultant 'missed' visit is not a milestone visit.
Injected at least 4 weeks ago	Re-treatment criteria not met	No injection	See at next scheduled appointment 4 or 8 weeks
Injection given less than 4 weeks ago	Re-treatment criteria met	No injection	See at next scheduled 4 weekly appointment or schedule additional 4 weekly appointment if participant on 8 weekly follow-up
Injection given less than 4 weeks ago	Re-treatment criteria not met	No injection	See at next scheduled 4 weekly appointment

Table 8.14.9.a: Management of Unscheduled visits

8.15 Withdrawal of Subjects

Participants have the right to withdraw from the study at any time and for any reason, without providing a reason. The investigator also has the right to withdraw participants from the study in the event of inter-current illness, AEs, SAEs, SUSARs, protocol violations or other reasons. Should a participant decide to withdraw from the study, they will be asked to volunteer a reason for withdrawal but are at liberty not to do so.

Should a participant withdraw from study drug only, efforts will be made to continue to obtain follow-up data, with the permission of the participant.

Subjects who withdraw from treatment early will be encouraged to return to the study site for an early termination assessment. All patients who withdraw from treatment will be encouraged to attend the milestone visits, i.e. baseline, weeks 12, 24, 52, 76 and 100. At any point, even after intervention has been declined, participants may resume treatment at the discretion of the site investigator, provided that they resume all study assessments and full visit scheduling. Investigators are encouraged to contact the Chief Investigator to discuss such cases if required.

8.16 Laboratory procedures

A urine pregnancy test will be processed by the study site using a standard pregnancy kit according to local practice. No samples will be processed at Centralised Laboratories.

9 Investigational Medicinal product

9.1 Name and description of all IMPs used in the trial

9.1.1 Aflibercept (2.0mg/50ul)

Aflibercept is a fusion protein that includes the key binding domains of human VEGF receptors 1 and 2 with human IgGFc and acts as a dummy receptor for all VEGF isoforms and placental growth factor preventing increased permeability and MO in CRVO. It is EMA licensed and NICE has recommended it for nvAMD and MO due to CRVO. The TA305 was published in February 2014 and NICE recommends this drug as first line use for this condition too. It is currently supplied in a glass vial to each Site Hospital Pharmacy direct from the manufacturer as a part of routine hospital stock. The vials may be replaced by pre-filled syringes during the study period.

9.1.2 Bevacizumab ((1.25mg/50µl)

Bevacizumab is a full length humanised monoclonal antibody that binds to VEGF A forming a protein complex incapable of binding to the VEGF receptor, thus blocking downstream VEGF action of increased vascular permeability and MO in CRVO. It is not licensed or recommended by the manufacturer for intraocular use but is used worldwide due to its low cost and relative ease of preparation in compounding pharmacies. It has not been compared with these two agents to date in MO caused by CRVO but has been found to be non-inferior to Ranibizumab in nvARMD. For this study it will be supplied in a sealed package containing a prefilled

syringe to each Site Hospital Pharmacy from the Liverpool and Broadgreen Pharmacy Manufacturing Unit, Royal Liverpool University Hospital, Prescot Street, Liverpool L78XP.

9.2 Name and description of the Comparator used in the trial

9.2.1 Ranibizumab (0.5mg/50µl)

Ranibizumab (0.5mg/50ul) is a humanised recombinant monoclonal antibody fragment that binds to VEGF A, preventing receptor interaction and blocking downstream action of VEGF, the key mediator of increased vascular permeability and macular oedema in central retinal vein occlusion. It is EMA licensed and NICE has recommended it for use in nvAMD, DMO and RVO. TA283 for MO due to RVO was issued in May 2013 and it has become the mainstay of routine clinical care for this condition since the third quarter of 2013, has largely superseded the use of Ozurdex, and will be the comparator for this study. It will be supplied in a glass vial, which may be replaced by a prefilled syringe during the study, to each Site Hospital Pharmacy direct from the manufacturer as a part of routine hospital stock.

9.3 Summary of findings from non-clinical studies

Please see section 3.3 Preclinical data and refer to the current version of the SPC on the eMC website: **http://www.medicines.org.uk/emc**.

9.4 Summary of findings from clinical studies

Please see sections 3.1 and 3.2 for Summary of clinical study findings and refer to the current version of the SPC for aflibercept, bevacizumab and ranibizumab on the eMC website: http://www.medicines.org.uk/emc

9.5 Summary of known and potential risks and benefits

Please see sections 3.4 Rationale and risks/benefits and 3.5 Assessment and management of risks. Further information in the current version of the SPC on the eMC website: http://www.medicines.org.uk/emc.

9.6 Source of active intervention and comparator

The comparator, ranibizumab (0.5mg/50ul) is used from routine Site Hospital Pharmacy stock. The intervention, aflibercept (2.0mg/50ul) is used from routine Site Hospital Pharmacy stock. The intervention, bevacizumab (1.25mg/50ul) will be supplied direct to each Site Hospital pharmacy from the Liverpool and Broadgreen Pharmacy Manufacturing Unit, Royal Liverpool University Hospital, Prescot Street, Liverpool L78XP who will be responsible for production, packaging, labelling, distribution and QP release.

9.7 Accountability procedures for the IMPs and the Comparator

9.7.1 Supply, Packaging and labelling of Investigational Medicinal Product

See Section 9.1.2. The bevacizumab will be packaged and labelled in accordance with Good Manufacturing Practice (GMP). Each treatment pack will be allocated a unique ID number, which will link directly to the online randomization system to ensure that the IMP supply is managed appropriately. The ranibizumab (0.5mg/ 50ul) and the aflibercept (2.0mg/50ul) will be supplied from normal Site Hospital Pharmacy stock.

9.7.2 Site Pharmacy Storage, Ordering and Handling Procedures

The Site Hospital Pharmacy will be responsible for receiving the comparator, ranibizumab (0.5mg/50ul) and two interventions, aflibercept (2.0mg/50ul) and bevacizumab (1.25mg/50ul) into the pharmacy and recording their delivery. It will also be responsible for ordering ranibizumab and aflibercept as part of normal hospital stock but the bevacizumab will be ordered by the Trial Manager for each site. Site Pharmacies must alert the Trial Manager if bevacizumab is required and should also let the Trial Manager know when a delivery has been received.

A study medication dispensing and return log will be maintained by the centre pharmacies. Administration records from the trial centres will be retained by the pharmacy department and monitored by the Trial Manager, to ensure that accurate CRF data are recorded. The randomization system will be linked to the IMP supply. The Hospital Site Pharmacy will also be responsible for appropriate storage, dispensing, disposal and recall and destruction logs in accordance with Good Manufacturing and Good Clinical practice and the Site Hospital pharmacies approved policies for IMP accountability and management. Furthermore, each site pharmacy will maintain a record of study drug administration based on the pre-printed form signed by the unmasked investigator that will be returned to the pharmacy at each centre (see Section 9.8).

9.7.3 Prescribing and dispensing procedures

Study medication will be prescribed by an authorised study physician according to the protocol, using a trial specific prescription. Medication will be dispensed according to local pharmacy practice. Documentation of prescribing, dispensing and return of the pre-printed form signed by the unmasked investigator will be kept in the pharmacy file and reconciled with the investigator site file at end of study. A study specific prescription must be submitted to pharmacy as early as possible after randomisation. The pharmacy will have received an email from the randomisation service at the time of randomisation, which must be printed and filed with the dispensing records and which will be referred to by the dispensing pharmacist to confirm whether the participant is correctly randomised to receive the drug.

Masking of treatment allocation: see Section 8.3.

9.8 Drug accountability

Used and unused Trial Study Medication & Study Medication Accountability: Each masking bag will contain a preprinted form which will detail the participant's unique pin number, date of birth, date drug dispensed and injection batch number. After performing the intravitreal injection, the unmasked injector will sign this form to confirm the drug has been given to the allocated patient and return it in the masking bag to the pharmacy. All used drug vials and syringes will be disposed of in the injection room and not returned to pharmacy. Pharmacy departments in each centre will maintain a study medication dispensing log, including date dispensed, batch number, expiry date and return log. The latter will be compiled from the form signed by the unmasked injector. In addition, the study specific prescriptions will be maintained in the pharmacy file for audit purposes. Any administration errors will be reported to the CI and trial statistician. In the event that an injection is not given as scheduled, reasons must be documented in the patients' notes and CRF. The study monitor will check the pharmacy records against the eCRF. All records will be reconciled at the end of the study with the Investigator Site File

9.9 Description and justification of route of administration and dosage

The approved route of administration, i.e. by intravitreal injection through the pars plana of the eye, will be used in all cases under sterile conditions in a designated treatment area in accordance with the Guidelines for Intravitreal Injection of the Royal College of Ophthalmologists (RCOphth) and any approved procedures for the individual Site Hospital. The injection can only be performed by the unmasked injector(s), who must be on the Hospital Site LEAVO study Delegation Log and experienced in intravitreal injection procedures. The dosage of ranibizumab, 0.5mg/50ul and aflibercept, 2.0mg/50ul used in this trial are the EMA approved and NICE recommended doses of these agents for intraocular use. The dosage for bevacizumab, 1.25mg/50ul is the dosage used in the IVAN and CATT clinical trials of treating wet ARMD and the standard dose used in clinical practice. Post injection checks will be in accordance with local hospital policy and may include VA, IOP or optic nerve head perfusion check or a combination of the above. The interval between two doses of all three drugs is not recommended to be less than 4 weeks and this will be adhered to throughout this study.

9.10 Dose modifications

Participants can only receive the specified dose of comparator i.e. ranibizumab, 0.5mg/50ul, and interventions, aflibercept, 2.0mg/50ul and bevacizumab, 1.25mg/50ul. No alterations in these doses are permissible and no alternative supplier of bevacizumab is permissible. The comparator and interventions can only be given in accordance with the protocol and no additional administrations of any study or non-study drug to treat macular oedema (e.g. topical steroids, periocular or intravitreal injection, systemic administration of oral steroid or acetazolamide) or alterations or omissions from the schedule are permissible.

9.11 Assessment of compliance

9.11.1 Protocol compliance

The study will run in accordance with the approved protocol. To ensure a standard approach to study conduct, site personnel will be trained in the protocol at the site initiation visit prior to starting recruitment at the site. Local sites should also contact the Chief Investigator or Trial Manager should any queries relating to the conduct arise. Trained clinicians will be administering the IMP. To ensure a standard approach to study conduct, site personnel will be trained in the protocol prior to starting recruitment. Participant unique PIN no., date of injection, drug, batch number and expiry date will be recorded in the pharmacy log to monitor compliance clinically.

9.11.2 Participant compliance

Clinical trials of DMO that require regular monthly follow-up visits showed that approximately 5% withdraw consent and 5% are lost to follow-up in similar sample-sized studies. Based on previous clinical trial experience of these patients at various sites selected for this study, the compliance rates of these patients to attend intervention and assessment schedules are good because of their fear of visual loss. The usual cause of non-compliance with visits for this type of study is other co-morbidities.

Sites will be instructed to follow up all participants for outcome data. Participants who are no longer receiving injections through the study should also be followed up and not be withdrawn. At the analysis stage, the data will be presented to the study statistician who will make a decision on the final classification of each participant. If a participant withdraws from the study, section 8.15 under Withdrawal of Subjects should be followed.

9.11.3 Compliance with medication

The drug will be administered at all times to the dosage, route of administration and schedule specified in the protocol.

9.12 Post-trial IMP arrangements

Bevacizumab 1.25mg/50ul will not be routinely available after the end of the trial for MO secondary to CRVO. Subjects exiting the trial who continue to require therapy for the condition will be followed up within their local NHS Trust Hospital clinical service and receive standard care for the condition which is likely to be either Ranibizumab (0.5mg/50ul) or Aflibercept (2.0mg/50ul).

9.13 Name and description of each Non-IMP (NIMP)

Prophylactic antibiotic eye drops may be prescribed post injection by any treating physician as per routine NHS practice and will be recorded as a concomitant medication for the study. Dose, duration and frequency

will be in accordance with local practice. Normal NHS prescribing practice in both primary and secondary care will apply with no special arrangements.

Intravenous fluorescein dye used to visualise the retinal circulation by obtaining retinal photographs is important for the initial grading of ischaemic vs non ischaemic CRVO and should be recorded as a concomitant medication for the study. Normal prescribing practice within secondary care will apply with no special arrangements.

9.14 Concomitant procedures

9.14.1 Pan-retinal photocoagulation in study eye

Either complete or sector panretinal photocoagulation to the study eye is permitted if an ischaemic CRVO or ocular neovascularisation is observed in any visit. The patient should then be seen at two weekly intervals until sufficient PRP is applied. The participants will also continue to attend all study visits until end of study. PRP will be recorded as a concomitant procedure.

Please refer to section 10.2.1 Planned hospitalisation, non-emergency procedures and SAE reporting

9.14.2 Vitrectomy surgery in study eye

A study eye in any arm may develop sight-threatening vitreous haemorrhage or retinal detachment. These conditions will be recorded as serious adverse events. Vitrectomy may be performed at the discretion of the investigator and will be recorded as a concomitant procedure.

Please refer to section 10.2.1 Planned hospitalisation, non-emergency procedures and SAE reporting

9.14.3 Cataract surgery in study eye

Anticipated need for cataract surgery in the study period is an exclusion criterion. Cataract may develop in the study eye and will be recorded as an AE. Planned cataract surgery will be allowed in the study eye if in the opinion of the investigator it is visually significant.

Please refer to section 10.2.1 Planned hospitalisation, non-emergency procedures and SAE reporting

9.14.4 Other surgery

Other surgery, eg. PRP to the fellow eye may be indicated in the case of a bilateral CRVO. The patient should then be seen at two weekly intervals until sufficient PRP is applied. The participant will also continue to attend all study visits until the end of study. PRP to the fellow eye will be recorded as a concomitant procedure.

Other planned procedures may be required in the study and non-study eye e.g. surgical repair of ptosis. The ptosis will be reported as an AE.

Please refer to section 10.2.1 Planned hospitalisation, non-emergency procedures and SAE reporting

9.14.5 Treatment of macular oedema in fellow eye

If macular oedema due to any retinal disease is present in the non-study eye, it is advocated that macular laser therapy be given as the first line therapy if appropriate. However, the participant can be treated with intravitreal anti-VEGF therapy or steroid therapy as per discretion of the treating physician. New onset macular oedema will be recorded as an adverse event. Laser therapy or intravitreal injection to the non-study eye will be recorded as a concomitant procedure.

9.14.6 Diagnosis and treatment of Infectious Endophthalmitis and other injection related procedures

Diagnosis and treatment of endophthalmitis is based on investigator judgement and local hospital policy. However, vitreous and aqueous cultures should be obtained and the intravitreal antibiotics used should be recorded as concomitant medications. Infectious endophthalmitis will be recorded as a Serious Adverse Event and will be reported as detailed in section 10.2.

Other injection related procedures e.g. retinal tear, retinal detachment and lens damage will be recorded as a Serious Adverse Event and will be reported as detailed in section 10.2.

9.14.7 Management of ischaemic CRVO, neovascular glaucoma, angle or iris neovascularisation

Ischaemic CRVO, NVA, NVI, NVG, NVE and NVD in the study eye will be recorded as adverse events. Diagnosis and management of these complications of CRVO in the study is based on investigator discretion and local practice. Laser therapy will form the mainstay of therapy and will be recorded as a concomitant procedure. Anti-VEGF agents in the study eye for NVG should be avoided.

9.14.8 Management of systemic complications and other co-morbidities

This will remain under the participant's medical care provider.

10 Recording and reporting of adverse events and reactions

10.1 Definitions

Term	Definition
Adverse Event (AE)	Any untoward medical occurrence in a patient or clinical trial subject administered a study intervention and which does not necessarily have a causal relationship with this treatment.
Adverse Reaction (AR)	Any untoward and unintended response in a subject to a study intervention which **is related** to any dose administered to that subject.

Adverse events can only be classified as serious if they meet the definition of serious:

Term	Definition
Serious adverse event (SAE), serious adverse reaction (SAR) or unexpected serious adverse reaction	Any adverse event, adverse reaction or unexpected adverse reaction, respectively, that:<br>1. results in death,<br>2. is life-threatening,<br>3. requires hospitalisation or prolongation of existing hospitalisation<br>4. results in persistent or significant disability or incapacity, or<br>5. consists of a congenital anomaly or birth defect
Important Medical Event	These events may jeopardise the subject or may require an intervention to prevent one of the above characteristics/consequences. Such events should also be considered ‘serious’.
Unexpected adverse reaction	An adverse reaction the nature and severity of which is not consistent with the information about the study intervention in question set out in the summary of product characteristics (aflibercept and ranibizumab) or the protocol (bevacizumab)
SUSAR	Suspected Unexpected Serious Adverse Reaction

10.2 Procedures for recording and reporting serious adverse events

All SAEs, SARs & SUSARs shall be recorded and reported on the serious adverse event form to the Chief Investigator / delegate within 24 hours of learning of its occurrence. The initial report can be made by completing the serious adverse event form, emailing or faxing to the KCTU (email: ctu@kcl.ac.uk, fax: 020 7848 5229,). A record of this notification (including date of notification) must be clearly documented to provide an audit trail. In the case of incomplete information at the time of initial reporting, a follow up report should be provided as soon as the information becomes available. The site will respond promptly to any queries raised by the Chief Investigator /delegate.

Relationship of the SAE to either study intervention should be assessed by the Principal Investigator/delegate (must be a clinician) at site. The Chief Investigator will be responsible for assessing, the expected or unexpected nature of any serious adverse reactions.

The Chief Investigator/delegate with the support of the KCTU will ensure that Moorfields Eye Hospital, as Sponsor is made aware of any SUSARs and SARs that occur. The Chief Investigator/delegate in conjunction with the Sponsor will be responsible for reporting all SUSARs to the MHRA and relevant ethics committee.

Reporting timelines are as follows:

- SUSARs which are fatal or life-threatening must be reported not later than 7 days after the sponsor is first aware of the reaction. Any additional relevant information must be reported within a further 8 days.
- SUSARs that are not fatal or life-threatening must be reported within 15 days of the sponsor first becoming aware of the reaction.

All Principal Investigators will be informed of all SAEs assessed as fulfilling criteria as a SUSAR (ie, possibly, probably or definitely related to either study intervention and unexpected as per the SPC or the protocol.

10.2.1 Planned "hospitalisations", non-emergency procedures and AE reporting

There are some AEs that meet the definition of serious (see section 10.2) but which do not require reporting on an SAE report form. Common ophthalmology and non-ophthalmology related events which result in ***planned, non-emergency*** hospital admissions for the investigation or treatment of those events and which **are not possibly, probably or definitely related to the IMPs** do not need to be reported on an SAE report form. These events should be recorded on the AE form and the investigation and treatment of ophthalmology related events only should also be recorded on the ophthalmology related concomitant procedure forms. All concomitant medications are recorded on the concomitant medication form. These forms should be updated following each study visit, to ensure the independent data monitoring committee receives accurate reports relating to the occurrence and treatment of adverse events.

Where a common ophthalmology or non-ophthalmology related event worsens or a complication arises as a result of the investigation or treatment of the event and subsequently meets the definition of serious, the reporting process in 10.2 will apply.

10.3 Assessments of Adverse Events

Each adverse event will be assessed for the following criteria:

Category	Definition
Mild	The adverse event does not interfere with the volunteer's daily routine, and does not require intervention; it causes slight discomfort
Moderate	The adverse event interferes with some aspects of the volunteer's routine, or requires intervention, but is not damaging to health; it causes moderate discomfort
Severe	The adverse event results in alteration, discomfort or disability which is clearly damaging to health

10.4 Causality

The assessment of relationship of adverse events to the administration of either study intervention is a clinical decision based on all available information at the time of the completion of the source data worksheets and should be performed by the Principal Investigator, or designee.

Whilst the Principal Investigator is responsible for resolving any queries that arise during the completion of the AE log and eCRF, queries can also be directed to the Chief Investigator and Trial Manager.

The following categories will be used to define the causality of the adverse event:

**Category**	**Definition**
Definitely	There is clear evidence to suggest a causal relationship, and other possible contributing factors can be ruled out.
Probably	There is evidence to suggest a causal relationship, and the influence of other factors is unlikely
Possibly	There is some evidence to suggest a causal relationship (e.g. the event occurred within a reasonable time after administration of either study intervention. However, the influence of other factors may have contributed to the event (e.g. the patient's clinical condition, other concomitant events).
Unlikely	There is little evidence to suggest there is a causal relationship (e.g. the event did not occur within a reasonable time after administration of either study intervention). There is another reasonable explanation for the event (e.g. the patient's clinical condition, other concomitant treatments).
Not related	There is no evidence of any causal relationship.
Not Assessable	Unable to assess on information available.

10.5 Expectedness

Category	Definition
Expected	An adverse event that is classed in nature as serious and which is consistent with the information about the study intervention listed in the SPC clearly defined in this protocol (aflibercept, ranibizumab and bevacizumab).
Unexpected	An adverse event that is classed in nature as serious and which is not consistent with the information about either study intervention in the SPC or clearly defined in this protocol (aflibercept, ranibizumab and bevacizumab).

The reference document to be used to assess expectedness against the study interventions and comparator is the SPC and the protocol. The protocol will be used as the reference document to assess disease related and/or procedural expected events and will take preference over the SPC.

Expected adverse events may be classified into ocular (study eye and non-study eye will be reported separately) and non-ocular. Ocular adverse events may be due to disease progression, injection procedure related, study intervention related or any other related event that the investigator deems clinically significant.

Disease progression: will include retinal neovascularisation, vitreous haemorrhage, conversion of non-ischaemic to ischaemic CRVO (defined as an increase in retinal haemorrhages in all 4 quadrants of the fundus associated with development of a relative afferent pupillary defect and an increase in capillary non-perfusion vs baseline on FFA if performed), iris or angle neovascularisation and neovascular glaucoma.

Injection related adverse events may include subconjunctival haemorrhage of greater than 1cm^2 in size, marked conjunctival hyperaemia, eye pain, transient reduced visual acuity, post injection floaters deemed clinically significant by the investigator, photopsiae, field defects or raised intraocular pressure > 30mmHg. Endophthalmitis, traumatic cataract, iatrogenic retinal hole or tear and any retinal detachment are serious adverse events.

Other related adverse events include allergic reaction to the fluorescein dye. Any confirmed APTC events will be documented and include vascular deaths, non-fatal myocardial infarction, non-fatal stroke, other thrombo-embolic events, non-ocular haemorrhage and recorded as SAEs.

10.6 Seriousness

Collection, recording and reporting of adverse events (including serious and non-serious events and reactions) to the sponsor will be completed according to the study specific SOPs.

10.7 Notification of deaths

Death will be treated as an SAE and should be reported in the same format as described in section 10.2Procedures for recording and reporting Serious Adverse Events.

10.8 Reporting SUSARs

The sponsor in conjunction with the Chief Investigator/delegate, will notify the main REC and MHRA of all SUSARs. SUSARs that are fatal or life-threatening must be notified to the MHRA and REC within 7 days after the sponsor has learned of them. Other SUSARs must be reported to the REC and MHRA within 15 days after the sponsor has learned of them.

10.9 Development Safety Update Reports

The Chief Investigator/delegate will prepare and submit a Development Safety Update Reports (DSUR) to the main REC and the MHRA in conjunction with the Sponsor's office. The report will be submitted within 60 days of the Developmental International Birth Date (DIBD) of the trial each year until the trial is declared ended.

10.10 Annual progress reports

The Chief Investigator/delegate will prepare and submit an annual progress report (APR) to the REC within 30 days of the anniversary date on which the favourable opinion was given, and annually until end of trial has been declared.

10.11 Pregnancy

In the event a female participant becomes pregnant, this should be reported to KCTU via fax or email (Fax: 020 7848 5229, email: ctu@kcl.ac.uk) using a pregnancy form as soon as the Investigator becomes aware of it. The pregnancy will be monitored to determine outcome. Any information related to the pregnancy following the initial report should be reported on a follow up pregnancy form.

Further treatment with any anti-VEGF therapy should be stopped on becoming aware of pregnancy but collection of outcome data should continue to the end of the study provided the participant is willing to do so.

Participants who wish to withdraw should be withdrawn as described in section 8.15 *Withdrawal of Subjects*

This process should also be followed if a female participant becomes pregnant within 6 months after their last trial injection regardless of whether they are still in follow up or not.

Any SAEs experienced during the pregnancy must be reported on an SAE form as described in 10.2 *Procedures for recording and reporting Serious Adverse Events*.

10.12 Overdose

In the event that a higher does is given to a participant, the site should notify the Chief Investigator/delegate. Follow up action will be decided on a case by case basis. Participants do not need to be withdrawn from the study and should remain on treatment and in follow up. Sites will be instructed to complete the adverse event form if such an event occurs.

10.13 Reporting Urgent Safety Measures

Any urgent safety measures taken should be immediately reported to the Chief Investigator or her assignee. Any queries that arise should be promptly resolved by the site to ensure reporting timelines are adhered to. The Chief Investigator /Sponsor shall immediately and in any event no later than 3 days from the date the measures are taken, give written notice to the MHRA and the relevant REC of the measures taken and the circumstances giving rise to those measures.

10.14 Type and duration of the follow-up of subjects after adverse events

Any ongoing AEs during the patient's participation in the study will be followed up until resolution. AEs, ARs, SAEs, SARs and SUSARs will be reportable for up to 30 days after the last intervention session.

10.15 Notification of Serious Breaches to GCP and/or the protocol

A "serious breach" is a breach which is likely to effect to a significant degree –

(a) the safety or physical or mental integrity of the subjects of the trial; or

(b) the scientific value of the trial.

The sponsor of a clinical trial shall notify the licensing authority in writing of any serious breach of –

(a) the conditions and principles of GCP in connection with that trial; or

(b) the protocol relating to that trial, as amended from time to time, within 7 days of becoming aware of that breach.

The Principal Investigator should notify the Chief Investigator/delegate if a serious breach in GCP/protocol is thought to have occurred as soon as he/she becomes aware of it. The Chief Investigator shall notify the sponsor as soon as he becomes aware of any case where the above definition applies during the trial conduct phase. The Chief Investigator will also notify the Trial Steering Committee and Data Monitoring Committee of serious breaches, throughout the course of the study. The Chief Investigator/delegate and the sponsor will be responsible for notifying serious breaches in GCP/protocol to the MHRA within the required timeframe and in line with Sponsor requirements.

11 Data management and quality assurance

11.1 Confidentiality

Data will be handled, computerised and stored in accordance with the Data Protection Act 1998. Participants will be identified via a unique PIN, date of birth and initials. Identifiable information will not be stored in the eCRF and will not leave the site. Any participant contact information will be stored within the site on password protected computers or within secured locations with limited access.

11.2 Data collection tools and source document identification

Written informed consent will be obtained prior to screening and any other study specific procedures are performed.

SAE data will be collected on paper SAE report forms and emailed or faxed to the KCTU. Summary details of SAEs will be transcribed to the adverse event section of the eCRF. For all other data collected, source data worksheets will be used for each patient and data will be entered onto the eCRF database. Source data worksheets will be reconciled at the end of the trial with the patients NHS medical notes in the recruiting centre. During the trial, critical clinical information will be written in the medical notes to ensure informed medical decisions can be made in the absence of the study team. Trial related clinical letters will be copied to the medical notes during the trial. The Principal Investigator will provide an electronic signature for each patient Case Record Form once all queries are resolved and immediately prior to database lock.

It will be the responsibility of the Principal Investigator and his team to ensure the accuracy of all data entered in the worksheets and the eCRF are in accordance with Good Clinical Practice. The delegation log will

identify all those personnel with responsibilities for data collection and handling, including those who have access to the trial database. The Principal Investigator will be responsible for ensuring that source data worksheets are filed in a suitably secure location to ensure source data verification can be undertaken throughout the study.

11.3 Data handling and analysis

All study data and site files will be kept at site in a secure location with restricted access.
The study will employ an eCRF created using the InferMed MACRO database system. Data will be managed via this system.

Database Website Address:
Go to www.ctu.co.uk and click the link to MACRO EDC V4 towards the top of the screen.

The eCRF will be created in collaboration with the trial statistician and the CI and maintained by the KCTU. It will be hosted on a dedicated secure server within KCL. This system is regulatory compliant (GCP, 21 CRF11, EC Clinical Trial Directive) and will have a full audit trail, data discrepancy functionality, database lock functionality, and supports real time data cleaning and reporting. The Trial Manager will be responsible for providing usernames and passwords to permitted local study personnel. Only those authorised by the Trial Manager will be able to use the system.

11.4 Quality assurance

The study incorporates a range of data management quality assurance functions. The eCRF system will contain a range of validations defined by the trial team that will alert sites to inconsistencies in the data being entered which will be monitored by the Trial Manager. The Trial Manager will provide study training, ongoing study support and will conduct regular monitoring visits at each centre, checking source data for transcription errors. Any necessary alterations to entered data will be date and time stamped within the eCRF A detailed monitoring plan and data management plan will be developed and updated as the trial progresses, detailing the quality control and quality assurance checks to be undertaken.

11.5 Database lock

Prior to database lock, the Trial Manager will review any outstanding warnings on the eCRF and resolve or close these as appropriate before database lock. Local study personnel should resolve any queries that arise promptly. Once all queries have been resolved no further changes will be made to the database unless specifically requested by the Study Office in response to the statistician's data checks. The study PI will review all the data for each participant and provide electronic sign-off to verify that all the data are complete and correct. At this point, all data will be formally locked for analysis. At the end of the trial, each centre will be supplied on a CD-ROM containing the eCRF data for their centre. This will be filed locally for

any future regulatory inspection or internal audit.

12 Record keeping and archiving

The Chief Investigator will be custodian for the data generated from the study. The Chief Investigator will be responsible for archiving the original data. All data will be archived for at least 5 years from the end of the trial and will be archived in accordance with Sponsor and regulatory requirements. Principal Investigators will be responsible for securely archiving local data generated, essential documents and source data in accordance with local requirements, but for at least 5 years from the end of the study. Investigators should provide archiving details to the Chief Investigator/delegate and will be instructed that authorisation from the Chief Investigator should be obtained before study data or study documentation is destroyed. Essential documents held by the KCTU will be returned to the Chief Investigator for archiving by the Sponsor organisation. eCRF data will also be exported and provided to the Chief Investigator for archiving.

13 Statistical Considerations

The trial statisticians will be responsible for all statistical aspects of the trial from design through to analysis and dissemination.

13.1 Outcomes

13.1.1 Primary outcome

Change in best corrected visual acuity from baseline to 100 weeks in the study eye of all patients measured in ETDRS letter score at 4 metres.

13.1.2 Secondary Outcomes

13.1.2.1 Visual Acuity and Clinical Outcomes

1. Change in best corrected visual acuity ETDRS letter score measured at 4 metres between baseline and 52 weeks.
2. A ≥ 15 ETDRS letter improvement (appreciable visual gain), a ≥ 10 letter improvement, a <15 letter loss and a ≥ 30 ETDRS letter loss (severe visual loss) at 52 and 100 weeks.
3. A ≥73 ETDRS letters or better than 6/12 Snellen equivalent (ie approximate driving visual acuity), a ≤58 ETDRS letters (≤6/24) and a ≤ 19 letters (≤3/60)(CVI partial and severe visual impairment) outcome at 52 and 100 weeks.
4. The change in OCT CST and macular volume from baseline at 52 and 100 weeks.
5. OCT CST < 320μm (Spectralis or refer to appendix 1) at 52 and 100 weeks (key guide to subsequent NHS clinical practice).
6. The number of injections performed in the study eye at 100 weeks.

7. Changes in the area of non-perfusion at 100 weeks.
8. Changes in OCT anatomical features over time and at 100 weeks.

13.1.2.2 Patient reported and cost-effectiveness outcomes

1. Quality of life scales (VFQ25 composite score, distance and near subscales, and EQ-5D with and without vision 'bolt-on') at 0, 12, 24, 52, 76 and 100 weeks.

2. Resource utilization (Client Service Receipt Inventories) at 0, 12, 24, 52, 76 and 100 weeks.

13.1.2.3 Safety and tolerability.

1. Occurrence of local and systemic side effects at 100 weeks
2. Development at week 100 i. to become a persistent non-responder (see Section 8.14.7) ii. of a change in retinal non-perfusion compared to screening iii. of anterior and posterior segment neovascularisation

13.1.2.4 Pre-specified sub-group analyses

1. To determine differences between arms in mean change in best corrected visual acuity at 100 weeks across baseline subgroup variables defined by i) baseline visual acuity stratified as ≤38 letters, 39-58 letters, 59-78 letters, ii) duration of disease stratified as: <3 months, 3-6 months and > 6 months, iii) treatment stratified as naïve vs previous treatment iv) quantity of retinal ischaemia (<10 , ≥10 and < 30, and ≥ 30 DA of non-perfusion).

13.2 Sample size recruitment

13.2.1 Sample Size Calculation

Bevacizumab and aflibercept are hypothesised to be substantially inferior to ranibizumab, if in each case, the mean of the primary outcome (change in best corrected ETDRS visual acuity letter score) is worse by a margin of five letters, a previously used non-inferiority margin (10), representing the minimum VA change a patient may distinguish. For CRVO, Campochiaro et al. (3) reported a standard deviation of 14.3 in the ranibizumab 0.5mg arm. 12-month lost to follow-up was 8.4% in ranibizumab arms. In the absence of 24-month data, we have assumed a comparable standard deviation (SD) of 14.3 at 100 weeks, and allowed for 15% dropout. The two null hypotheses, that bevacizumab is substantially inferior to ranibizumab, and that aflibercept is substantially inferior to ranibizumab, will each be rejected if the estimated 95% confidence interval for the difference in treatment means lies wholly above the five letter margin in each case. Assuming equal efficacy, there will be 80% power to reject each null hypothesis and declare non inferiority with 130 followed-up patients analysed per arm. Allowing for 15% missing data at 100 weeks, 459 patients will be randomized to the three arms (equal allocation ratio; 153 per arm) for the CRVO patient group. Sample size calculations were performed using nQuery Advisor 4.0 software. The primary method of analysis will be a

linear mixed effects model with adjustment for baseline which is expected, other things being equal, to increase the power to detect non-inferiority.

The primary method of analysis will include all available refracted data of the primary outcome up to and including 100 weeks, including data from the 15% of patients we anticipate could be missing the 100 weeks primary outcome endpoint, thereby giving flexibility to provide increased power or a higher dropout allowance for the stated power without having to amend the sample size in this event.

13.2.2 Planned recruitment rate

Approximately 40 sites will be opened and recruit into this study. It is anticipated that 459 participants will be recruited over an 18 months period. The DMEC will receive recruitment updates and based on committee recommendations new sites will be added as needed.

13.3 Statistical analysis plan

A detailed statistical analysis plan was completed before the start of the trial and commented on by the DMEC and approved by the TSC. The plan is accompanied by a Health Economics Analysis Plan, and is updated and re-approved by the TSC when the protocol is amended.

13.3.1 Summary of baseline data and flow of patients

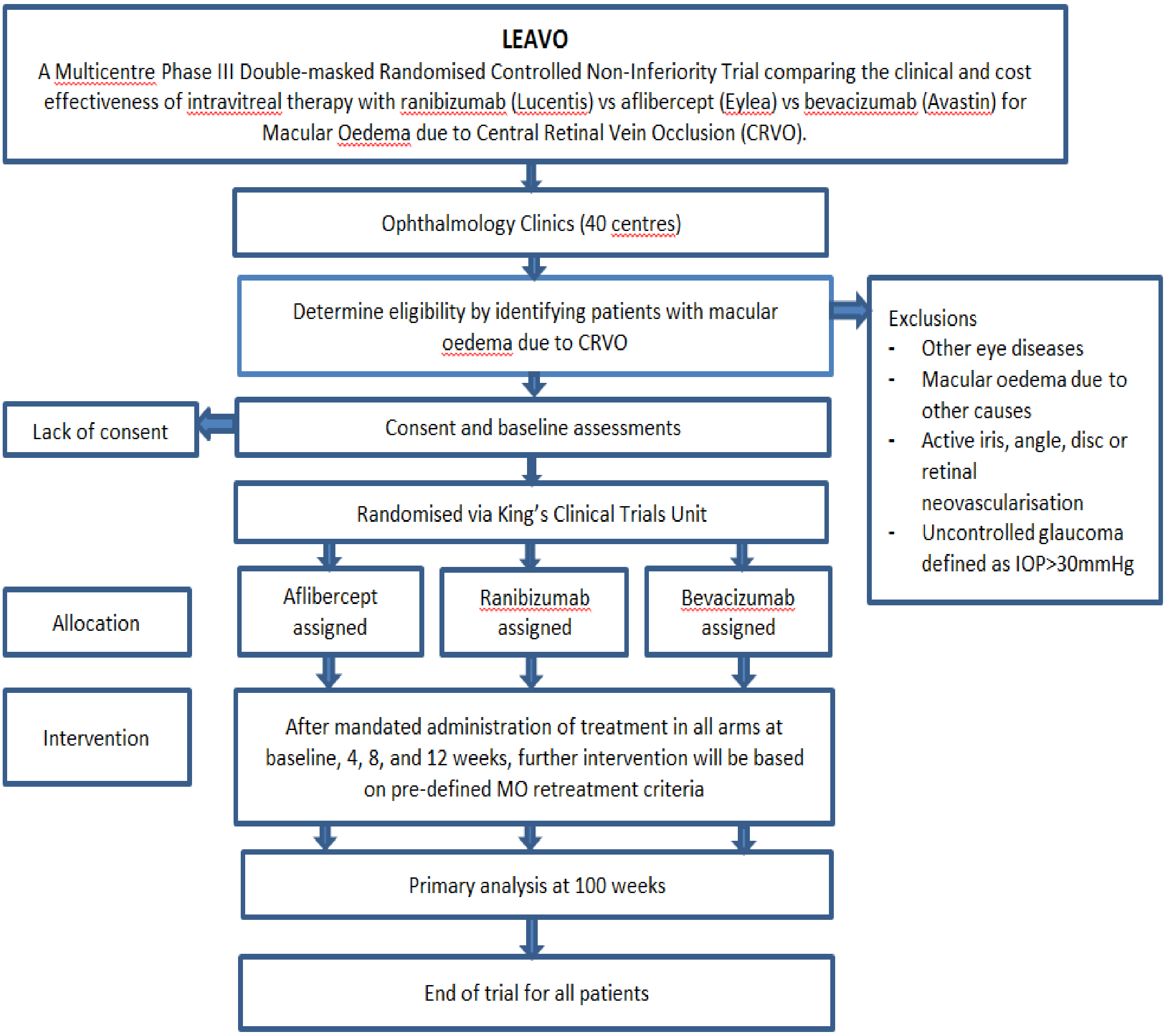

13.3.2 Primary outcome analysis

Analyses will be on both an intention to treat (ITT) basis and a Per Protocol (PP) basis. The primary outcome will be compared between arms primarily at the 100-week point and secondarily at the 52-week point using a linear mixed effects model with patient as a random effect to allow for within-patient correlation of repeated measures over time. The fixed effects will consist of arm, time, the continuous form of the baseline of the outcome using the missing indicator method, the remaining minimisation stratifiers and the interactions of these with time. The test for non-inferiority will be one-sided at the 2.5% significance level, and presented as an estimated effect with two-sided 95% confidence interval compared against the non-inferiority margin. Treatment effect estimates and confidence intervals at a time point will be obtained directly from the model by

setting that time point as the reference.

For the analysis of the primary outcome, the mixed effects model will be re-fitted in a reduced per protocol (PP) population, defined as the subset of patients found to be eligible at entry and who had minimal sufficient exposure to the treatment regimen, defined as 4 treatments correctly assessed and received during the first 6 visits up to week 20. For each of the first four visits, a correct treatment is defined as receiving the injection. For the 5th and 6th visits, a correctly assessed and received treatment is defined to be the receipt of an injection where this is indicated to be required by the retreatment criteria or the non-receipt of an injection where this is indicated by the retreatment criteria. Non-inferiority will only be concluded if this is declared by both the ITT analysis and the PP analysis at 100 weeks. Non-inferiority will also be assessed in ITT and PP populations at 52 weeks.

13.3.3 Secondary outcome analysis

Secondary outcome analyses will be on an ITT basis only, and assessed with tests at the two-sided 5% level of significance. Continuous outcomes will be compared between arms using a linear mixed effects model, as specified for the primary outcome ITT analysis, incorporating prior measurements of the outcome over time. Binary outcomes will be compared between arms using logistic regression. Continuous and binary outcomes will be reported as adjusted differences in means or odds ratios respectively. All tests will be two-sided at the 5% significance level and interpreted cautiously with a focus on interpreting effect sizes with 95% confidence intervals. Safety outcomes will be reported as unadjusted patient proportions and rates within and between arms with 95% confidence intervals using exact methods where appropriate.

13.3.4 Sensitivity and other planned analyses

Sensitivity to the missing at random assumption made in the primary outcome analysis will be undertaken to assess sensitivity to the handling of missing 100-week data, and to the use of concomitant treatments, and will be detailed in the statistical analysis plan.

If non-inferiority is concluded for either of the investigational treatments, then superiority will be assessed. If non-inferiority is concluded for both the investigational treatments then there will be a formal test of superiority to compare these two investigational treatments.

13.4 Randomisation methods

Only one eye can be randomised into the trial. In 95% of cases, one eye will be affected by CRVO and will be the ‘worst seeing eye’ and will therefore be randomised. On rare occasions, some patients may have bilateral CRVO that meet the eligibility criteria. In these cases the worst-seeing eye will be randomised unless the patient opts for the ‘better seeing eye’ to be randomised.

Randomisation will be via a bespoke web based randomisation system hosted at the KCTU. 459 adult patients with MO due to CRVO will be randomised 1:1:1 at the level of the individual using the method of minimisation incorporating a random element. The three stratifying factors are visual acuity (stratified by screening BCVA letter score (≤38 [approximate Snellen equivalent ≤6/60], 39–58 [approximate Snellen

equivalent 6/48 to 6/24], ≥59 [approximate Snellen equivalent ≥ 6/18]), duration of disease from date of CRVO diagnosis to commencement of therapy (< 3 months, 3-6 months and > 6 months) and treatment naïve vs previous treatment.

13.5 Interim analysis

Formal interim analysis of the primary outcome for early stopping is not planned for this study. Regular interim reports will be prepared as needed for DMEC meetings.

13.6 Other statistical considerations

A detailed statistical analysis plan is in place and was agreed with the DMC and TSC prior to the availability of primary outcome data being supplied to the study statisticians.

14 Name of Committees involved in trial

14.1 Trial Steering Committee (TSC)

The TSC is the Committee, responsible for monitoring the overall integrity, conduct and safety of the trial. It will monitor its progress; investigate any serious adverse events; and take account of regular reports from the DMEC and communication from the TMG. Ultimate responsibility for any decision required on the trial's continuation will lie with the TSC. The Committee will include an Independent Chair, a Professor of Statistics, an Independent Ophthalmologist and General Physician, Consultant in Public Health, Senior Department of Health Policy Maker, two principal investigators and two patient representatives. TSC meetings will take place at least annually and these will be arranged by the Chief investigator and the Trial Manager in conjunction with the Chair. Increased frequency of meetings will be arranged depending on the requirements of the study DMEC and TSC recommendations. An NIHR HTA CET representative and Moorfields Eye Hospital representative (Sponsor) may also be invited.

14.2 Data Monitoring and Ethics Committee (DMEC)

.

An independent DMEC of three persons, one Professor of Statistics and two Retina Specialists will meet regularly, to safeguard the interests of trial participants, assess the safety and efficacy of the interventions during the trial, and monitor the overall conduct of the clinical trial. Its terms of reference are to receive and review the progress and accruing data of the trial and provide advice and recommendations on trial conduct to the Trial Steering Committee. The study may be prematurely discontinued on the basis of new safety information, or for other reasons given by the DMEC and/or TSC, Sponsor, regulatory authority or Research Ethics Committee concerned. All data reviewed by the DMEC will determine safety issues. All serious adverse reactions will be reported to the KCTU within 24 hours of learning of their occurrence.

14.3 Trial Management Group (TMG)

The TMG will be responsible for monitoring the delivery of the trial on a day to day basis and will be supported and managed via the KCTU. The TMG membership will consist of: Chief Investigator, Co-Lead, Trial Manager, Data Manager, the Lead and Trial Statistician(s) and Senior Members of KCTU. Other members of the wider research team may be invited on a meeting by meeting basis depending on the scope covered.

15 Direct Access to Source Data/Documents

Monitoring of study conduct and data collected will be performed by a combination of central review and site monitoring visits to ensure the study is conducted in accordance with GCP. Study site monitoring will be undertaken by the Trial Manager. The main areas of focus will include consent, serious adverse events, and essential documents in study site files.

Site monitoring will include:

- Reviewing all consent forms within the site file and medical notes.
- Source data verifying serious adverse events against medical records and a proportion of the primary outcome measure.
- Checking essential documents in the investigator site file and study files.

Central reviews will include:

- Ensuring accuracy and completeness of all applications for study authorisations and submissions of progress/safety reports, prior to submission
- Ensuring all documentation essential for study initiation is in place prior to site authorisation
- Reporting and following up all monitoring findings with the appropriate persons in a timely manner.

The investigator(s)/ institution(s) will also permit trial-related monitoring, audits, REC review, and regulatory inspection(s), providing direct access to source data/documents. Trial participants are informed of this during the informed consent discussion. Participants will consent to provide access to their medical notes.

16 Ethics and regulatory requirements

16.1 Ethical issues

The main ethical issues in relation to this study are the use of intravitreal injections. However, this is now standard of care for wet age related macular degeneration, diabetic macular oedema and retinal vein occlusion. There are at least 5 extra visits that the participants need to undergo in excess of standard of care. The precise risks and benefits of participating in the clinical study will be outlined in patient information sheets, formulated with service user involvement.

Both ranibizumab and aflibercept are licensed for use in this indication. Bevacizumab is not licensed for intravitreal use but it is the most widely used anti-VEGF agent worldwide and has been shown to be non-inferior to ranibizumab in nvAMD.

Any breach of confidentiality will be minimised by adherence to the UK Data Protection Act 1998 and the approved protocol

16.2 Approval requirements

The Chief Investigator with the support of KCTU and sponsor will ensure that the trial protocol, patient information sheet, consent form, GP letter and submitted supporting documents have been approved by the appropriate regulatory body (MHRA in UK) and a main REC, prior to any patient recruitment. The protocol and all agreed substantial protocol amendments, will be documented and submitted for ethical and regulatory approval (as appropriate) prior to implementation.

Before the site can enrol patients into the trial, the Chief Investigator/Principal Investigator or designee must apply for NHS permission from their Trust Research & Development (R&D) and be granted written permission. It is the responsibility of the Chief Investigator/ Principal Investigator or designee at each site to ensure that all subsequent amendments gain the necessary approval. This does not affect the individual clinician's responsibility to take immediate action if thought necessary to protect the health and interest of individual patients (see section 10.13 on reporting urgent safety measures).

Within 90 days after the end of the trial, the Chief Investigator/Sponsor will ensure that the main REC and the MHRA are notified that the trial has finished. If the trial is terminated prematurely, those reports will be made within 15 days after the end of the trial.

The Chief Investigator will supply the Sponsor with a summary report of the clinical trial, which will then be submitted to the MHRA and main REC within 1 year after the end of the trial.

16.3 Monitoring requirement for the trial

The Trial Manager will conduct source data verification as described in section 15 on *Direct Access to Source Data/Documents*.

17 Finance

The study is funded through the NIHR HTA CET Programme.

18 Insurance

The participating NHS Trusts have liability for clinical negligence that harms individuals towards whom they have a duty of care. NHS indemnity covers NHS staff and medical academic staff with honorary contracts conducting the trial. There are no arrangements for non-negligent compensation.

19 Publication policy

The data will be the property of and publication will be the responsibility of the Chief Investigator. It is planned to publish this study in peer review journals and to present data at national and international meetings. Results of the study will also be reported to the Sponsor and Funder, and will be available on their web site. All manuscripts, abstracts or other modes of presentation will be reviewed by the Trial Steering Committee and Funder prior to submission. Individuals will not be identified from any study report. A copy of the results of the study will also be available to participants if a copy is requested. No personal data of participants will be detailed in any publication submitted.

20 Statement of compliance

The trial will be conducted in compliance with the approved protocol, the UK Regulations and as amended and GCP.

21 References

(1) Williamson T et al. Central retinal vein occlusion: what's the story ? Br J Ophthalmol 1997; 81:698-704.

(2) NICE FAD: ranibizumab for macula oedema secondary to retinal vein occlusion. 2013 April,NICE. www.NICE.org (accessed 27-04-2014).

(3) Campochiaro PA et al. Sustained benefits from ranibizumab for macular edema following central retinal vein occlusion: twelve-month outcomes of a phase III study. Ophthalmology 2011;118:2041-9.1)

(4) Clarkson JG et al. Evaluation of Grid pattern Photocoagulation for Macular Edema in Central Vein Occlusion.CRVO Group M Report. Ophthalmology 1995; 102:1425-1433.

(5) Heier JS et al. Ranibizumab for macular edema due to retinal vein occlusions: long-term follow-up in the HORIZON trial. Ophthalmology. 2012;119:802-9.

(6) Holz et al. VEGF-Trap-Eye for macular oedema secondary to central retinal vein occlusion. Br J Ophthalmol 2013:97:278-284.

(7) Brown DM et al, Intravitreal aflibercept for macular edema secondary to central retinal vein occlusion: 1 year results from the Phase III COPERNICUS study. Am J Ophthalmol 2013;155:429-437.

(8) Bevacizumab in Eye Conditions: Issues related to Quality, Use, Efficacy, and Safety. Aug 2012. www.nicedsu.org.uk/Macular-oedema-bevacizumab *(accessed 27-05-2013)*

(9) Chakravarthy et al. Alternative treatments to inhibit VEGF in age-related choroidal neovascularisation: two year findings of the IVAN randomised controlled trial. Lancet 2013, 140-6736(13) 61501-9

(10) Martin DF et al. Ranibizumab and bevacizumab for treatment of neovascular age-related macular degeneration: two-year results, CATT Research Group. Ophthalmology 2012;119:1388-98.

(11) Curtis LH et al, Risks of mortality, myocardial infarction, bleeding, and stroke associated with therapies for age-related macular degeneration. Arch Ophthalmol. 2010 Oct;128(10):1273-9.

(12) Carneiro AM et al. Arterial thromboembolic events in patients with exudative age-related macular degeneration treated with intravitreal bevacizumab or ranibizumab. Ophthalmologica. 2011;225(4):211-21.

(13) Rajendram R, Fraser-Bell S, Kaines A, Michaelides M, Hamilton RD, Esposti SD, Peto T, Egan C, Bunce C, Leslie RD, Hykin PG. A 2-year prospective randomized controlled trial of intravitreal bevacizumab or laser therapy (BOLT) in the management of diabetic macular edema: 24-month data: report 3. Arch Ophthalmol. 2012 Aug;130(8):972-9.

(14) Ding X et al. Prospective study of intravitreal triamcinolone acetonide versus bevacizumab for macular edema secondary to central retinal vein occlusion. Retina. 2011; 31:838-45.

(15) Epstein DL et al. Benefit from bevacizumab for macular edema in central retinal vein occlusion: twelve-month results of a prospective, randomized study. Ophthalmology. 2012;119:2587-91.

(16) NICE FAD: ranibizumab for macula oedema secondary to retinal vein occlusion. 2014 January,NICE. www.NICE.org (accessed 27-04-2014).

(17) Chakravarthy U et al. Ranibizumab versus bevacizumab to treat neovascular age-related macular degeneration: one-year findings from the IVAN randomized trial. Ophthalmology. 2012;119:1399-411.

22 Appendices

Appendix 1: SD-OCT machines and associated CST

SD-OCT machines	Upper limit of normal Central Sub-field Thickness (µm)
Heidelberg Spectralis	320um
Zeiss Cirrus	300
Topcon - 1000	300
Topcon - 2000	300
Optovue RTVue-100	300

In the unlikely event that a site uses an SD-OCT machine not listed above, this will be dealt with on a case by case basis and the upper limit of normal for CST determined.

A Multicentre Phase III Double-masked Randomised Controlled Non-Inferiority Trial comparing the clinical and cost effictiveness of intravitreal therapy with ranibizumab (Lucentis) vs aflibercept (Eylea) vs bevacizumab (Avastin) for Macular Oedema due to Central Retinal Vein Occlusion (CRVO)

Statistical Analysis Plan

Version 5.4

EudraCT no: 2014-000272-26

ISCRN: 13623634

CONTENTS

1.0 Introduction........8
1.1 Derivation of the statistical analysis plan........8
1.2 Purpose and scope of the statistical analysis plan........8
2.0 Overview of the condition and treatment........9
2.1 Description of the condition and its importance/scale........9
2.2 Description of the standard treatment (or placebo or current care)9
2.3 Description of the investigational treatments........9
2.4 Description of the motivation for the study / need to investigate the new treatment........10
3.0 Populations and Study Sample........10
3.1 Target Population........10
3.2 Trial Population........10
3.3 Trial Samples........12
3.3.1 Intention To Treat (ITT)........12
3.3.2 Per Protocol (PP)........12
3.4 Inclusion and Exclusion criteria........13
4.0 Objectives, principal research question and associated hypotheses14
4.1 Principal Trial objective........14
4.2 Principal Research Question........14
4.3 Hypotheses........14
4.4 Study objectives........15
4.4.1 Primary objective:........15
4.4.2 Secondary Objectives........15
5.0 Trial design........17
5.1 Treatment arms........17
5.2 Type of RCT........17
5.3 Frequency and duration of follow-up........17
6.0 Trial measures........17
6.1 Primary outcome........17
6.2 Secondary outcomes........18
6.2.1 Continuous outcome variables:........18
6.2.2 Categorical outcome variables:........18
6.3 Timing of measures........18
6.4 Participant duration in the study........20
6.5 Final assessment........20

7.0 Sample Size 20
7.1 Determination of the primary outcome effect size 20
7.2 Determination of the primary outcome variability 20
7.3 Clustering of outcomes from eyes within subjects effects 20
7.4 Power to detect effects 21
7.5 Determination of the sample size based on the primary outcome 21
7.6 Detectable effects sizes expressed in general standardised form 21
8.0 Randomisation and Subgroups 21
8.1 Arms 21
8.2 Method of allocation 21
8.3 Relative timing of randomisation 21
8.4 Subgroup variables 23
9.0 Blinding 23
10.0 Data and Distributions 23
10.1 Data decisions made 23
10.2 Outcomes requiring derivation 23
10.2.1 Procedure for deriving variables 24
10.2.2 Missing items in scale and subscales 24
10.3 Use of data transformation 24
10.4 Defining Outliers 24
10.5 Handling outliers 24
11.0 Descriptive analysis 26
11.1 Flow diagram 26
11.2 Baseline comparability of randomised groups 26
11.3 Comparison of rates of adherence and follow-up 27
12.0 Analysis covariates 27
12.1 Stratifiers 27
12.2 Baseline 27
13.0 Primary outcome analysis 27
13.1 Statistical Model 27
13.2 Intention to Treat Strategy 28
13.3 Per Protocol analysis 28
13.4 Concluding non-inferiority 28
13.5 Superiority 28
13.6 Subgroup analysis 28
13.7 Sensitivity to missing data 29

13.8 Sensitivity analysis to use of concomitant treatments 30
13.9 Interim analysis 30
14.0 Secondary outcome analysis 30
14.1 Analysis of continuous outcomes 30
14.2 Analysis of binary outcomes 30
14.3 Analysis methods for secondary outcomes 30
15.0 Handling multiple comparisons 31
16.0 Software 32
17.0 DMC monitoring 32
18.0 Acknowledgments 32
19.0 Amendments to Versions 32
Reference list 34
Appendix I – Lists of Tables for DMC and for main trial 36
Appendix II – Record of data decisions during the blinded review 38
21.1 Record of data decisions 38
21.2 Record of analysis decisions 38
Appendix III – Record of data decisions after the blind-break 39
22.1 Record of data decisions ..... Error! Bookmark not defined.
22.2 Record of analysis decisions 39
Signatures 40

ABBREVIATIONS:

BCVA	Best Corrected visual acuity
CFP	Colour Fundus Photography
eCRF	Electronic Case Report Form
CSRI	Client Service Receipt Inventory
CST	Central Subfield Thickness
CTU	Clinical Trials Unit
CVI	Certificate of Visual Impairment
DA	Disk areas
DMC	Data Monitoring Committee
DMO	Diabetic Macular Oedema
EMA	European Medicines Agency
ERM	Epiretinal Membranes
EQ-5D	Euro Quality of life questionnaire
ETDRS	Early Treatment Diabetic Retinopathy Study
FDA	Food and Drug Administration
FFA	Fundus Fluorescein Angiography
HEDMAP	Health Economic and Decision Modelling Analysis Plan
ICH	International Conference on Harmonization
IMP	Investigational Medicinal Product
ISRCTN	International Standard Randomised Clinical Trials Number
ITT	Intention to treat
LME	Linear mixed effects model
NPDR	Non Proliferative Diabetic Retinopathy
NVD	Neovascularisation Disc
NVE	Neovascularisation elsewhere
OCT	Optical Coherence Tomography
PDR	Proliferative Diabetic Retinopathy
PP	Per protocol
RCT	Randomised Control Trial
REC	Research Ethics Committee
SAR	Serious Adverse Reaction
SAP	Statistical Analysis Plan
SOP	Standard Operating Procedure
TSC	Trial Steering Committee
UK	United Kingdom
VA	Visual Acuity
VAS	Visual Analogue Scale
VEGF	Vascular Endothelial Growth Factor
VFQ-25	Visual Function Questionnaire
VMT	Vitreomacular Traction

INVESTIGATORS:

Trial Statistician:

Name: Joana Vasconcelos
Address: Imperial Clinical Trials Unit, Stadium House, 68 Wood Lane, Imperial College London, London W12 7RH
Email: joana.vasconcelos@imperial.ac.uk

Lead Statistician:

Name: Professor Toby Prevost
Address: Imperial Clinical Trials Unit, Stadium House, 68 Wood Lane, Imperial College London, London W12 7RH
Email: a.prevost@imperial.ac.uk

Chief Investigator:

Name: Mr. Philip Hykin
Address: Moorfields Eye Hospital. 162, City Road, London EC1V 2PD.
Email: Philhykin@aol.com

Co-lead:

Name: Professor Sobha Sivaprasad
Address: Moorfields Eye Hospital & UCL Institute of Ophtalmology. 162, City Road, London EC1V 2PD
Email: sobha.sivaprasad@nhs.net

Chair of Trial Steering Committee:

Name: Miss Susan Downes
Address: Oxford Eye Hospital, Oxford University Hospitals, West Wing, John Radcliffe Hospital, Headley Way, Oxford OX3 9DU
Email: susan.downes@ouh.nhs.uk

Chair of Data Monitoring Committee:

Name: Prof Sarah Walker
Address: Professor of Statistics,
Medical Research Council Clinical Trials Unit (MRC CTU) Aviation House, 125 Kingsway, London, WC2B 6NH
Email: :asw@ctu.mrc.ac.uk, t: 020 7670 4726, f: 020 7670 4969

Sponsor:

Name: Ms Maria Hassard
Address: NIHR Moorfields Biomedical Research Centre, Moorfields Eye Hospital & UCL Institute of Ophthalmology 162 City Road, London, EC1V 2PD
Email: maria.hassard@moorfields.nhs.uk

Funder:

Name: NIHR HTA CET – National Institute for Health Research, Health Technology Assessment Programme, Clinical Trials and Evaluation Stream
Contact name: Simon Bevan
Email: s.bevan@soton.ac.uk

1.0 Introduction

1.1 Derivation of the statistical analysis plan

The present statistical analysis plan was derived from the trial protocol, by the trial statistician, Joana Vasconcelos, with the supervision of Professor Toby Prevost. The trial statistician is responsible for developing the SAP as well as for carrying out the statistical analysis for interim and final statistical reporting of the trial. The supervisor will revise the SAP and give an overall verification of the analysis throughout the study, in keeping with the Standardised Operating Procedures (SOPs) of the King's Clinical Trials Unit, including the SOP for developing the Statistical Analysis Plan.

The formation of this Plan has drawn on statistical guidance from: the ICH Harmonised Tripartite Guideline: Statistical Principles for Clinical Trial E9 and E3(1), the CONSORT statement for reporting trials(2), the Committee for Medicinal Products for Human Use (formerly known as the Committee for Proprietary Medicinal Products) report(3), from general issues in non-inferiority designs(4, 5) and from specific trial issues (6, 7).

The trial statistician will write the first version of the plan. After revision by the supervisor the plan will be filed as version number 2. The plan will then be discussed with the Principal Investigator for further input and filed as version number 3. The plan will then be sent to the DMC and TSC for final approvals and saved as version 4 and 5 of the plan, respectively.

1.2 Purpose and scope of the statistical analysis plan

The purpose of this Statistical Analysis Plan is to set out the study objectives and hypotheses, and the analytical approaches and procedures necessary to address these for the main trial paper and to provide guidance for further research reported in other papers, promoting consistent approaches and methods.

As there can typically be more than one analytical approach to address a hypothesis, there is the potential for different results to be produced from using alternative approaches, alternative methods, alternative outcome definitions and the alternative data that may be involved. These differences can be influential, for example, when results are of borderline statistical significance.

Therefore, this Plan records those decisions that can be made about study hypotheses, outcome definitions and statistical procedures, along with their basis and the appropriateness of the assumptions required for their use, in advance of the main trial analysis, while any access to unmasked follow-up data and to trial arm is prevented.
Changes within subsequent versions of the Plan prior to analysis will be dated, with the basis for the changes reasoned, and recorded within the plan.

Other analysis decisions may need to be made later, based on viewing the observed distribution of the data. Where possible these decisions will be made prior to access to trial arm, or where necessary from control arm data alone. The main place for this will be the "pre-analysis review" phase, blinded to treatment arm (ICH E9 (1)), taking place after final participant follow-up and prior to the study arm being made available. Prior to the first DMC

meeting with follow-up data, indications, such as on the need for data transformation, will be made at a point when baseline data only is available without access to arm.

Decisions will be supported by reasoning and justification, and these will be appended to the Statistical Analysis Plan, and dated, to provide a record of any post-analysis decisions and their basis.

It is not intended that the strategy set out in the plan should prohibit sensible practices. However, the principles established in the plan will be followed as closely as possible when analysing and reporting the trial.

2.0 Overview of the condition and treatment

2.1 Description of the condition and its importance/scale

Retinal Vein Occlusion (RVO) is a blockage of the small retinal veins by a blood clot. When the blood cannot drain away from the retina, there is an accumulation of pressure in the blood vessels resulting in leakage of fluid and blood causing macular oedema and ischemia. This condition can affect the central retinal vein (CRVO) (formed by the union of the four retinal veins (one retinal vein drains each quarter of the eye)) or a major branch retinal vein (BRVO), where blockage occurs somewhere along the course of one of the four retinal veins.CRVO is characterised by retinal haemorrhages, venous dilatation and tortuosity in all four quadrants of the retina and is typically more severe than BRVO(8, 9).

Approximately 6,860 people develop CRVO every year in England and Wales of whom 5,150 are potentially eligible for treatment. Once established, the visual impairment due to CRVO is typically profound with no tendency to improve spontaneously. Without intervention permanently impaired visual loss is likely to occur. In this study the focus will be on CRVO.

2.2 Description of the standard treatment (or placebo or current care)

Until 2011 no treatment was available to improve vision in people with CRVO. In 2011, NICE recommended the NHS an implant called “Ozurdex”, which although improving vision, could cause cataracts and glaucoma with repeated use, was difficult to administer and had only moderate uptake in the UK. In the meantime another treatment, more effective with fewer side effects, Ranibizumab (Lucentis, Novartis, & Genentech), was approved by the FDA and EMA for macular oedema due to CRVO. Ranibizumab is a humanized, affinity-matured VEGF antibody fragment that binds to and neutralizes all isoforms of VEGF-A and their biologically active degradation products and it was the first anti-VEGF therapy to demonstrate improved visual outcomes in patients with neovascular age related vascular degeneration. It is EMA licensed for use in wet age related macular degeneration, diabetic macular oedema and retinal vein occlusion and NICE recommended for all three.

2.3 Description of the investigational treatments

This study aims to determine if the two anti-VEFG agents Bevacizumab or Afilbercept are as effective as Ranibizumab in reducing visual loss from MO due to CRVO, whether they have

an equivalent side effect profile and whether either could be considered as a recommended NHS treatment based on non-inferior clinical effectiveness and superior cost-effectiveness.

Aflibercept or VEGF Trap-Eye (Eylea, Bayer/Regeneron), is a fusion protein of the key domains of VEGF receptors 1 and 2 and human IgGFc that blocks all VEGF-A isoforms and placental growth factor. Like ranibizumab, it is EMA licensed for nvAMD, DMO and RVO. It is FDA approved for CRVO and NICE has recommended this drug for MO due to CRVO (TA 305).

Bevacizumab (Avastin, Genetech/Roche), is a monoclonal antibody that inhibits vascular endothelial growth factor (VEGF), a mediator in the pathogenesis of common and disabling eye disorders including neovascular age related macular degeneration (nvAMD), diabetic macular oedema (DMO) and retinal vein occlusion (RVO). Bevacizumab is EMA licensed for the treatment of cancer but not for use in the eye. There is limited evidence regarding its use in central retinal vein occlusion (CRVO) with macular oedema (MO). To date, bevacizumab has been found to be non-inferior to ranibizumab for all visual acuity primary and secondary endpoints in nvAMD in the IVAN and CATT studies (10, 11).

2.4 Description of the motivation for the study / need to investigate the new treatment

This project will compare the relative clinical and cost effectiveness of the anti-VEGF agents bevacizumab, aflibercept, and ranibizumab in MO due to CRVO over 100 weeks which is of critical importance to the NHS in the next 10 years. The NICE Final Appraisal Document for ranibizumab in RVO has recommended that further research is required comparing ranibizumab and bevacizumab (12) and there are no comparisons to date of bevacizumab and ranibizumab with aflibercept. This trial will be the first well-powered Phase III trial exploring the relative effectiveness of these drugs and aflibercept in the management of MO in CRVO. This study will inform us of the potential use of the most clinically effective and cost-effective drug for this condition in the NHS by providing a better understanding of the economic and societal impact of RVO in the long-term and help decision makers evaluate and compare these medical interventions over the duration of the natural history of the condition.

3.0 Populations and Study Sample

3.1 Target Population

The *target population*, to which inferences from the end of this trial are intended to generalise, is the population of adult patients with MO due to CRVO.

3.2 Trial Population

The trial population, from which the study sample is drawn, is further defined to be adults aged 18 year or over, of less than 12 months duration who attend the 40 ophthalmology centres in the UK with expertise in retinal disorders and a proven track record in effective research.

Only one eye per patient will be included in the trial. In subjects with both eyes meeting the eligibility criteria, then the 'worst seeing eye' will be enrolled unless the patients preference is for the best seeing eye (see section 7.3).

3.3 Trial Samples

3.3.1 Intention To Treat (ITT)

The achieved trial sample comprises those patients who consent to participate and are actually randomised into this trial. These patients are the study subjects.

This randomised trial sample is also the trial Intention To Treat (ITT) population. The intention-to-treat principle states that every subject will be analysed according to the treatment group to which they were randomised. In this trial, subjects' data will be analysed according to the *Intention to Treat Strategy* (13), under which at least one analysis is recommended to be based on the ITT population.

The trial ITT population comprises all randomised participants, regardless of eligibility (inclusion/exclusion) error, post-randomisation withdrawal, and whether the correct study treatments were received, or other interventions received.

3.3.2 Per Protocol (PP)

Definition

A per protocol set of subjects will also be included. These will be defined as the subset of the found to be eligible at entry and who had minimal sufficient exposure to the treatment regimen, defined as 4 treatments correctly assessed and received during the first 6 visits up to week 20. For each of the first four visits, a correct treatment is defined as receiving the injection. For the 5th and 6th visits, a correctly assessed and received treatment is defined to be the receipt of an injection where this is indicated to be required by the retreatment criteria or the non-receipt of an injection where this is indicated by the retreatment criteria..

Rationale

The main reason for having a per protocol set comes from the fact that this is a non-inferiority trial and so the use of the full analysis set is generally not conservative (ICH E9 section 5.2.3 (1)). As Lesaffre 2008 (7) states, "*dropouts and a poor conduct of the study might direct the results of the two arms towards each other*". Although this can be interpreted as an indication that the per protocol analysis is the conservative choice for non-inferiority studies Garrett AD 2003 (6) state that "*The perceived conservative nature of the PP population appears to be much more a reflection of reduced patient numbers than the presence of bias, while bias can be in either direction depending on the pattern of violations*". Moreover, with two active treatments it may be more likely that any bias affecting both treatments would be reduced in comparison to a placebo-controlled trial.

Prominence

Non-inferiority will only be declared if both ITT and the PP analysis are supportive of a non-inferiority conclusion. This is supported by the Committee on Proprietary Medical Products Points-to-Consider (5) and several other papers (7, 14).

The requirement to declare noninferiority in both the ITT and the PP analyses promotes the adherence to treatment protocol and the minimisation of exclusions, maintaining power.

3.4 Inclusion and Exclusion criteria

The inclusion and exclusion criteria are the following:

Inclusion criteria:
1. Subjects of either sex aged ≥ 18 years.
2. Clinical diagnosis of centre-involving macular oedema (MO) due to CRVO
3. CRVO of ≤ 12 months duration.
4. Best corrected visual acuity in the study eye ≥ 19 and ≤ 78 ETDRS letters (approximate Snellen VA 3/60 to VA 6/9).
5. Best corrected visual acuity in the non-study eye ≥ 14 ETDRS letters (approximate Snellen VA ≥ 2/60.
6. SD-OCT central subfield retinal thickness (CST) > 320µm (Spectralis) predominantly due to MO secondary to CRVO in the study eye.
7. Media clarity, pupillary dilatation and subject cooperation sufficient for adequate fundus imaging of the study eye.
8. In cases of bilateral CRVO, if both eyes are potentially eligible, unless the patient prefers otherwise, the worst seeing eye will be recruited.

Exclusion criteria (applied to the study eye only and to the non study eye only where specifically stated):
1. Macular oedema considered to be due to a cause other than CRVO (e.g. diabetic macular oedema, Irvine-Gass syndrome).
2. An ocular condition is present that, in the opinion of the investigator, might affect macular oedema or alter visual acuity during the course of the study (e.g. vitreomacular traction).
3. Any diabetic retinopathy or diabetic macular oedema at baseline clinical examination of the study eye.
4. Moderate or severe non proliferative diabetic retinopathy (NPDR) or quiescent, treated or active proliferative diabetic retinopathy (PDR) or macular oedema in the non-study eye. Note: Mild NPDR only is permissible in the non-study eye.
5. History of treatment for MO due to CRVO in the past 90 days with intravitreal or peribulbar corticosteroids or in the last 60 days with anti-VEGF drugs or > 6 prior anti-VEGF treatments in the previous 12 months.
6. Active iris or angle neovascularisation, neovascular glaucoma, untreated NVD, NVE and vitreous haemorrhage or treatment for these conditions in the last 1 month.
7. Uncontrolled glaucoma [>30mmHg], either untreated or on anti-glaucoma medication at screening.
8. Any active periocular or intraocular infection or inflammation (e.g. conjunctivitis, keratitis, scleritis, uveitis, endophthalmitis).

<u>Systemic exclusion criteria:</u>
9. Uncontrolled blood pressure defined as a systolic value > 170mmHg and diastolic value > 110mmHg.
10. Myocardial infarction, stroke, transient ischaemic attack, acute congestive cardiac failure or any acute coronary event < 3 months before randomisation.
11. Women of child bearing potential unless using effective methods of contraception throughout the study and for 6 months after their last injection for the trial. Effective contraception is defined as one of the following:
    a. Barrier method: condoms or occlusive cap with spermicides.

b. True abstinence: When it is in line with the preferred and usual lifestyle of the subject. Periodic abstinence (e.g. calendar, ovulation, symptothermal, post-ovulation methods) and withdrawal are not acceptable methods of contraception.
c. Have had tubal ligation or bilateral oophorectomy (with or without hysterectomy).
d. Male partner sterilisation. The vasectomised male partner should be the only partner for the female participant.
e. Use of established oral, injected or implanted hormonal methods of contraception and intrauterine device.

12. Pregnant or lactating women.
13. Males who do not agree to an effective form of contraception for the duration of the study and for 6 months after their last injection for the trial.
14. Hypersensitivity to the active ingredients aflibercept, bevacizumab or ranibizumab or any of the excipients of these drugs.
15. Hypersensitivity to Chinese Hamster Ovary (CHO) cell products or other recombinant human or humanised antibodies.
16. A condition that, in the opinion of the investigator, would preclude participation in the study.
17. Participation in an investigational trial involving an investigational medicinal product within 90 days of randomisation.

4.0 Objectives, principal research question and associated hypotheses

4.1 Principal Trial objective

The objective of the LEAVO trial is to determine if Bevacizumab or Afilbercept are as effective as Ranibizumab in reducing visual loss from MO due to CRVO, whether they have an equivalent side effect profile and whether either could be considered as a recommended NHS treatment based on non-inferior clinical effectiveness and superior cost-effectiveness.

4.2 Principal Research Question

The principle research question is as follows: Is visual acuity following Aflibercept or Bevacizumab non-inferior to Ranibizumab in eyes with MO due to CRVO at 100 weeks?

4.3 Hypotheses

The hypotheses refer to the populations of relevant patients rather than study subjects.

The *Working hypothesis*: The so-called "working hypothesis" is the hypothesis which motivates the trial, which the trial results may or may not support. It is that the change in best corrected visual acuity is non-inferior in patients treated with either Aflibercept or Bevacizumab compared to patients treated with Ranibizumab.

The Statistical *Null Hypothesis* 1: Bevacizumab is inferior to Ranibizumab in eyes with MO due to CRVO at 100 weeks.
The Statistical *Null Hypothesis* 2: Aflibercept is inferior to Ranibizumab in eyes with MO due to CRVO at 100 weeks.

Statistical *Alternative hypothesis* 1: Bevacizumab is noninferior to Ranibizumab in eyes with MO due to CRVO at 100 weeks.
Statistical *Alternative hypothesis* 2: Aflibercept is noninferior to Ranibizumab in eyes with MO due to CRVO at 100 weeks.

4.4 Study objectives

4.4.1 Primary objective:

1. To determine whether bevacizumab is non-inferior to ranibizumab in treating visual loss due to MO secondary to central retinal vein occlusion at 100 weeks
2. To determine whether aflibercept is non-inferior to ranibizumab in treating visual loss due to MO secondary to central retinal vein occlusion at 100 weeks.

4.4.2 Secondary Objectives

1. To determine the difference between arms in mean change in best corrected visual acuity at 52 weeks.
2. To determine the difference between arms in the proportion of participants with ≥ 15 ETDRS letter improvement (appreciable visual gain), ≥ 10 letter improvement, <15 letter loss and ≥ 30 ETDRS letter loss (severe visual loss) at 52 and 100 weeks.
3. To determine the difference between arms in the proportion of participants with ≥73 ETDRS letters or better than 6/12 Snellen equivalent (ie approximate driving visual acuity), ≤58 ETDRS letters (≤6/24) and ≤ 19 letters (≤3/60)(CVI partial and severe visual impairment) at 52 and 100 weeks.
4. To determine the difference between arms in the mean change in OCT CST and macular volume at 52 and 100 weeks.
5. To determine the difference between arms in the proportion of participants with OCT CST < 320µm (Spectralis or refer to protocol appendix 1) at 52 and 100 weeks (key guide to subsequent NHS clinical practice).
6. To determine the differences between arms in the mean number of injections performed per study eye per participant at 100 weeks.
7. To determine any differences in the relative effectiveness of the investigational treatments and comparator on quality of life and resource utilization, reported as Incremental Cost Effectiveness Ratios (ICERs) at 52 weeks.
8. To determine any differences in the relative effectiveness of the investigational treatments and comparator on quality of life and resource utilisation, reported as Incremental Cost Effectiveness Ratios (ICERs) at 100 weeks.
9. To detect any differences in the prevalence of local and systemic side effects at 100 weeks.
10. To determine differences between arms at 100 weeks in the proportion i. of persistent non-responders (see protocol Section 8.14.7), ii. of participants that develop a change in retinal non-perfusion compared to screening iii. of participants that develop anterior and posterior segment neovascularisation.
11. To determine differences between arms in mean change in best corrected visual acuity at 100 weeks due to i) baseline visual acuity stratified as ≤38 letters, 39-58 letters, 59-78 letters, ii) duration of disease stratified as: <3 months, 3-6 months and > 6 months, iii) treatment stratified as naïve vs previous treatment iv) quantity of retinal ischaemia (<10, ≥10 and < 30, and ≥ 30 DA of non-perfusion).
12. To determine differences between arms in changes in area of non-perfusion at 100 weeks and OCT anatomical features over time and at 100 weeks.

5.0 Trial design

This is a two year multicentre (approximately 40 centres), double-blind pragmatic individually randomised controlled trial that will test the non-inferiority visual acuity from treatment with Bevacizumab and Aflibercept to Ranibizumab at 100 weeks in 459 adult participants with MO due to CRVO of less than 12 months duration.

5.1 Treatment arms

The trial is randomised with three arms and with equal allocation of participants in a 1:1:1 ratio to the three arms.

Arm A: Treatment: An intravitreal injection of Aflibercept (Eylea, Bayer) (2.0mg/5µl) will be administered at baseline, 4, 8 and 12 weeks. After this the retreatment criteria is ascertained (see Protocol section 8.14).

Arm B: Treatment: An intravitreal injection of Bevacizumab (Avastin, Roche) (1.25mg in 50ul) will be administered at baseline, 4, 8 and 12 weeks. After this the retreatment criteria is ascertained (see Protocol section 8.14).

Arm C: Control: An intravitreal injection of Ranibizumab (Lucentis, Novartis) [0.5mg/50ul] will be administered at baseline, 4, 8 and 12 weeks. After this the retreatment criteria is ascertained (see Protocol section 8.14).

Different labels can be used for the control arm, such as placebo, comparator, standard care, or control.

5.2 Type of RCT

This is a phase III, parallel groups' trial.

5.3 Frequency and duration of follow-up

Participants in all 3 study arms will be seen at weeks 0, 4, 8, 12, 16, 20 and 24. After this participants will potentially be seen every 4 weeks until week 96 if retreatment criteria are met. If retreatment criteria are not met at three successive visits from week 24 onwards the visit interval is increased to 8 weekly until week 96. They will also be finally seen at 100-weeks.

6.0 Trial measures

6.1 Primary outcome

The primary outcome is Best Corrected Visual Acuity (BCVA) in the study eye measured in ETDRS letter score at 4 metres at 100 weeks. Measurements of BCVA at milestone visits are included in the analysis of the primary outcome. Any BCVA measurement will be excluded from the analysis if it is both more than 3 standard deviations below the mean at that timepoint (including all measurements) AND taken within 3 months of occurrence of a vitreous haemorrhage or another cause unrelated to maculopathy secondary to CRVO (such as neovascular glaucoma).

6.2 Secondary outcomes

The secondary efficacy outcome measures are listed as follows according to the type of variable they will be formally analysed at 52 weeks and 100 weeks, but also measured at other time points.

6.2.1 Continuous outcome variables:

i. Visual Acuity and Clinical Outcomes

1. Change from baseline in ETDRS letter score measured at 4 metres at 52 weeks.
2. Change from baseline in mean OCT central subfield thickness (CST) at 52 and 100 weeks.
3. Change from baseline in macular volume at 52 and 100 weeks.
4. Number of injections performed in the study eye at 100 weeks
5. Change in retinal non-perfusion as assessed by mean disc area of non-perfusion at 100 weeks and by the ischaemic index at 100 weeks.

ii. Patient reported outcomes

1. National Eye Institute visual function questionnaire (VFQ25) composite score, distance, and near subscales at 52 and 100 weeks.
2. Quality of life (EQ-5D with and without vision bolt-on) at 52 and 100 weeks.

iii. Economic reported outcomes (this is detailed in the health economics analysis plan)

1. Quality of life scales (VFQ25 composite score, distance and near subscales, and EQ5D with and without vision bolt-on) at 0, 12, 24, 52, 76 and 100 weeks.
2. Resource utilization (Client Service Receipt Inventories) at 0, 12, 24, 52, 76 and 100 weeks.

6.2.2 Categorical outcome variables:

i. Visual Acuity and Clinical Outcomes

1. Participants with ≥15 ETDRS letter improvement (appreciable visual gain), ≥ 10 letter improvement, <15 letter loss and ≥ 30 ETDRS letter loss (severe visual loss) at 52 and 100 weeks.
2. Participants with ≥73 ETDRS letters or better than 6/12 Snellen equivalent (i.e. approximate driving visual acuity), ≤58 ETDRS letter (≤6/24) and ≤19 letters (≤3/60) (CVI partial and severe visual impairment) at 52 and 100 weeks.
3. Participants with OCT CST <320µm (Spectralis or refer to protocol appendix 1) at 52 and 100 weeks (key guide to subsequent NHS clinical practice).
4. Participants with the anatomical OCT features: diffuse intraretinal oedema, intraretinal cystic change, subretinal fluid, vitreomacular interface abnormaility (either VMT or ERM) over time and at 100 weeks.

ii. Safety and tolerability

1. Prevalence of local and systemic side effects at 100 weeks.
2. Participants that are persistent non-responders (section 8.14.7 of the protocol) and that develop anterior and posterior segment neovascularisation at 100 weeks.

6.3 Timing of measures

A full schedule on the timing of measures is provided in below:

^Mandatory Visits: Loading (wk 4 & 8) & Milestones (baseline, wks 12, 24, 52, 76, 100)	Screening	Baseline	Week 4	Week 8	Week 12	Week 16	Week 20	Week 24		Week 52		Week 76		**Week 100	Unsch. Visit.	**Withdrawal Visit
Variable treatment visits									4-8 weekly		4-8 weekly		4-8 weekly			
Weeks		0	4	8	12	16	20	24	28-48	52	56-72	76	80-96	100	1-99	13-97
Visit window (days)	-10 to 0	0	0 to +14	0 to +14	0 to +14	-14 to +14	-14 to +14	-14 to +14	-14 to +14	-14 to +14	-14 to +14	-14 to +14	-14 to +14	-14 to +14		
Informed Consent	X															
Inclusion/Exclusion Criteria review	X	X[3]														
Randomisation[1]		X														
Urine Pregnancy test in women of child bearing age.	X															
Patient demographics, medical and ophthalmic history	X															
Adverse events	X	X	X	X	X	X	X	X	X	X	X	X	X	X	X	X
Concomitant medication	X	X	X	X	X	X	X	X	X	X	X	X	X	X	X	X
Blood Pressure	X	X	X	X	X	X	X	X	X	X	X	X	X	X	X	X
Best corrected ETDRS visual acuity in both eyes (refraction visit =X1)	X1	X	X	X	X1	X	X	X1	X	X1	X	X1	X	X1	X / X1[5]	X1
Standard Ophthalmic Examination	X	X	X	X	X	X	X	X	X	X	X	X	X	X	X	X
SD-OCT in both eyes	X		X	X	X	X	X	X	X	X	X	X	X	X	X	X
7-field or wide-angle CFP[2]	X									X				X	+/-[5]	X
7-field or wide angle FFA[2]	X													x	+/-[5]	X
VFQ-25 and EQ-5D with and without vision 'bolt-on'		X			X			X		X		X		X	+/-[5]	X
CSRI		X			X			X		X		X		X	+/-[5]	X
Treatment Allocation Guess Form[4]														X		X
Administer IMP*		X	X	X	X	X2	X2	X2	X2	X2	X2	X2	X2		X2	X

X1 – Same day refracted best corrected visual acuity
X2 - PRN treatment.
Study Treatment Visit: non shaded square.
Study Milestone Visit: shaded square
^Milestone visits and mandated loading visit dates should be agreed with participant prior to performing randomisation
*Intravitreal injections including immediate post injection checks are performed as per each trial sites local policy and may include a check of ON perfusion or VA or IOP or a combination of these.
** Participants should be reminded to use an effective form of contraception for 6 months after their last trial injection. Females of child bearing potential should be reminded to notify the local study team if they fall pregnant during this time.
[1]Randomisation should only occur once all other assessments at baseline (week 0) have occurred
[2]Further colour fundus photographs and fluorescein angiography may be performed as per investigator discretion. Colour fundus photographs should be done if a patient converts from non-ischaemic to ischaemic CRVO.
[3]To include review of screening assessment test results and confirmation of eligibility.
[4]To be completed by participant, masked investigator, site optometrists.
[5]To be performed (as required) if unscheduled visit is a milestone visit.

6.4 Participant duration in the study

Each study subject will participate in the trial from the day that the they give informed consent to their last final visit at 100 weeks.

6.5 Final assessment

The final study assessment is when the last study subject achieves their 100 weeks assessment.

7.0 Sample Size

7.1 Determination of the primary outcome effect size

Bevacizumab and aflibercept are defined to be substantially inferior to ranibizumab, if in each case, the mean of the primary outcome (change in best corrected ETDRS visual acuity letter score) is worse by a margin of five letters.

The two null hypotheses, that bevacizumab is substantially inferior to ranibizumab, and that aflibercept is substantially inferior to ranibizumab, will each be rejected if the estimated 95% confidence interval for the difference in treatment means lies wholly above the five letter margin in each case.

The choice of a five-letter margin is 32% higher than the available estimated 12-month placebo-controlled effect of 6.6 letters (15) for ranibizumab, the standard (comparator) treatment for LEAVO. This margin choice is therefore consistent with maintaining assay sensitivity sufficiently to be able to declare non-inferiority.

7.2 Determination of the primary outcome variability

For a similar trial on CRVO (15) the standard deviation reported in Ranibizumab arms at 12 months was 14.3. In the absence of 24-month data we have assumed a comparable SD of 14.3 at 24 months.

7.3 Clustering of outcomes from eyes within subjects effects

Only one eye per subject can be selected for the study. In 95% of cases, one eye will be affected by CRVO. As explained in the protocol, bilateral RVO is rare, but if it happens and both eyes are eligible, the eye included is the 'worst seeing eye'. However, participants will be given the choice if both eyes are found to be eligible. All observations are in this way able to be assumed to be independent in the sample size calculation and statistical analysis.

7.4 Power to detect effects

There is 80% power to detect non-inferiority using a two-sided 95% confidence interval from an analysis of covariance test with adjustment for baseline visual acuity and randomisation stratifiers.

7.5 Determination of the sample size based on the primary outcome

The sample size was set to be 459 participants, 153 per arm (1 eye per subject). The target of 390 subjects followed up with primary outcome required in the analysis involves a 15% allowance for dropout and protocol exclusions.

Sample size calculations were performed using nQuery Advisor 4.0 software.

7.6 Detectable effects sizes expressed in general standardised form

For a continuous secondary outcome, with 153 subjects per arm followed up we can detect effects of size 0.45 Standard Deviation's difference between means with 80% power using a two-sided t-test at the 5% significance level. For binary outcomes, we have at least 90% power to detect a difference in proportions of 0.2 using a chi-squared test at the 5% significance level.

8.0 Randomisation and Subgroups

8.1 Arms

Each participant will be equally randomised to one of three arms: bevacizumab, aflibercept or ranibizumab.

8.2 Method of allocation

The method of minimisation incorporating a random element will be used. There will be three stratifying factors: visual acuity (stratified by baseline BCVA letter score (≤38 [approximate Snellen equivalent <6/60], 39–58 [approximate Snellen equivalent between 6/48 and 6/24 exclusive], ≥59 [approximate Snellen equivalent ≥ 6/18]) and onset of symptoms to presentation at hospital and commencement of therapy (< 3 months, 3-6 months and > 6 months) and prior treatment or not.

8.3 Relative timing of randomisation

Randomisation will be via a bespoke web based randomisation system hosted at the King's CTU on a secure server. Once a participant enters the study and their data is entered into the

eCRF, they will be allocated a unique study PIN. This, along with their date of birth and initials will be used to identify the participant and their data throughout the study.

8.4 Subgroup variables

Three subgroup variables will be considered: i) baseline visual acuity (low, moderate, high: ≤38 letters, 39-58 letters, 59-78 letters), ii) disease duration (<3 months, ≥3 months) and iii) quantity of retinal ischaemia (non-ischaemic vs ischaemic vs very ischaemic CRVO (<10, ≥10 and ≥ 30 DA of non-perfusion) .

These are based on the fact that visual gain in the low vision group may be higher than that achieved by the high vision group and this effect may be different between arms. Patients with ischaemic CRVO may not have similar visual acuity gains to those with no ischaemia and this effect may be different between treatment arms. The shorter the duration of disease, the better the visual acuity outcomes but this may vary between treatment arms.

9.0 Blinding

The trial will be double masked. Study participants, clinicians and members of the research team who will undertake key measurements (visual acuity, morphology) will be masked to group allocation. The clinician administering the drug injected into the vitreous will not be masked. This will ensure that the study has a high level of both treatment integrity (delivery of the treatment as intended) and treatment differentiation (treatment conditions differed from one another in the intended manner).

The trial statistician will have access to the accumulating outcome data that is required for reporting to the DMC.Both the trial statisticians will attend both the open and closed DMC meetings.

10.0 Data and Distributions

10.1 Data decisions made

The data manager will make limited decisions about data variables and values so that issues such as missing data can be comprehensively handled by the trial statistician. Decisions which impact on the analysis will be recorded in an appendix of this statistical analysis plan.

10.2 Outcomes requiring derivation

List of outcomes with source of derivation code:

1) **VFQ-25** (16): a validated tool for vision related quality of life. It consists of a base set of 25 vision targeted questions representing 11 vision-related sub-scales, plus an additional single-item general health rating question. The overall composite score is computed as the simple average of the vision-targeted sub-scale scores, excluding the general health rating question. The overall score can range from 0 (worst possible score) to 100 (best).

   EQ-5D (17-19) with and without vision bolt-on: The EQ-5D is a generic instrument for describing and valuing health. It is based on a descriptive system that defines health in terms of 5 dimensions (Mobility, Self-care, Usual activities,

Pain/Discomfort, Anxiety/Depression. Each dimension has 5 response categories (EQ-5D-5L) corresponding to eg. “no problems",”slight problems”, “moderate problems”, “severe problems”, and “unable to/extreme problems”. A preference-based score ranges from states worse than dead (<0) to 1 (full health), anchoring dead at 0. In addition, the EQ-5D includes a visual analogue scale (EQ-VAS), which records the respondent’s self-rated health on a vertical scale where the endpoints are labelled ‘Best imaginable health state’ (marked as 100) and ‘Worst imaginable health state’ (marked as 0).

The EQ-5D with bolt-on is similar to the EQ-5D-5L but another dimension was added (vision) in order to overcome perceived inadequacies in a particular population. The corresponding scoring system for the EQ-5D vision 'bolt-on' on has not been finalised yet. Further details are developed in the HEDMAP.

10.2.1 Procedure for deriving variables

If there is existing syntax code to derive a variable within the King's Clinical Trials Unit then this will be used. Otherwise new code will be developed by the trial statistician and verified by the senior statistician.

10.2.2 Missing items in scale and subscales

The number (%) of patients with complete data for each scale will be reported. If scales provide missing value guidance then this will be used.

10.3 Use of data transformation

It is not anticipated that any continuous outcomes will need to be considered for transformation, because the sample size is reasonably large for group comparisons in the main trial analyses. Assumptions of normality and constant variance required by the models will be examined using residual and other diagnostic plots. If it is relevant, and necessary, where sample size is reduced, a log transformation will be considered, because this retains a sensible interpretation for inferences; in relative terms between arms. If an absolute interpretation is needed, then data transformation may not be undertaken, but a nonparametric Bootstrap method for obtaining confidence intervals may be considered (20).

10.4 Defining Outliers

Outliers are observations that have extreme values relative to other observations observed under the same conditions. An outlier will be defined here as a data-point being at least four standard deviations from the mean of its distribution of values observed across other patients. This definition will apply to the transformed scale for those outcomes that have been log transformed.

A “bivariate outlier‟ for checking will be defined here as a pair of successive serial data-points of the same measure for a participant whose difference is at least four standard deviations from the mean of all patients’ such differences. Simple plots of successive pairs of serial measures will be used through the 24-month period to assist in identifying outliers for data checking.

10.5 Handling outliers

Outliers will be identified for further investigation by looking at the distributions of the data through histograms, scatter plots or box-plots. Univariate tests for the compatibility of the distribution with a normal distribution will not be undertaken since they can be too sensitive to departures that are often not relevant for the comparison of means (Central Limit Theorem).

Once an outlier is found, a blinded member of the team with sufficient clinical experience will be involved in the decisions as to whether a data value is impossible versus implausible versus plausible. If the outlier is impossible, then it will be set to missing, and a list of these occurrences will be appended to this SAP. If an outlier is clinically plausible, the outlier will remain. If an outlier is clinically implausible (but possible), it will not be ignored or deleted but will be retained for ITT analysis.

If outliers remain in the distribution of a variable, then data transformations or nonparametric methods of analysis may be considered.

Sensitivity analysis will be undertaken to check whether the outlier is influential by obtaining results with and then without inclusion of the outlier. If the conclusions are changed, then this will be noted.

11.0 Descriptive analysis

11.1 Flow diagram

The flow diagram of the study is the one below. This will include the number randomised, who comprise the intention to treat and per protocol population, and the numbers followed-up to be in the analyses of the primary outcome as well as the main reasons for missing data by stages of the trial.

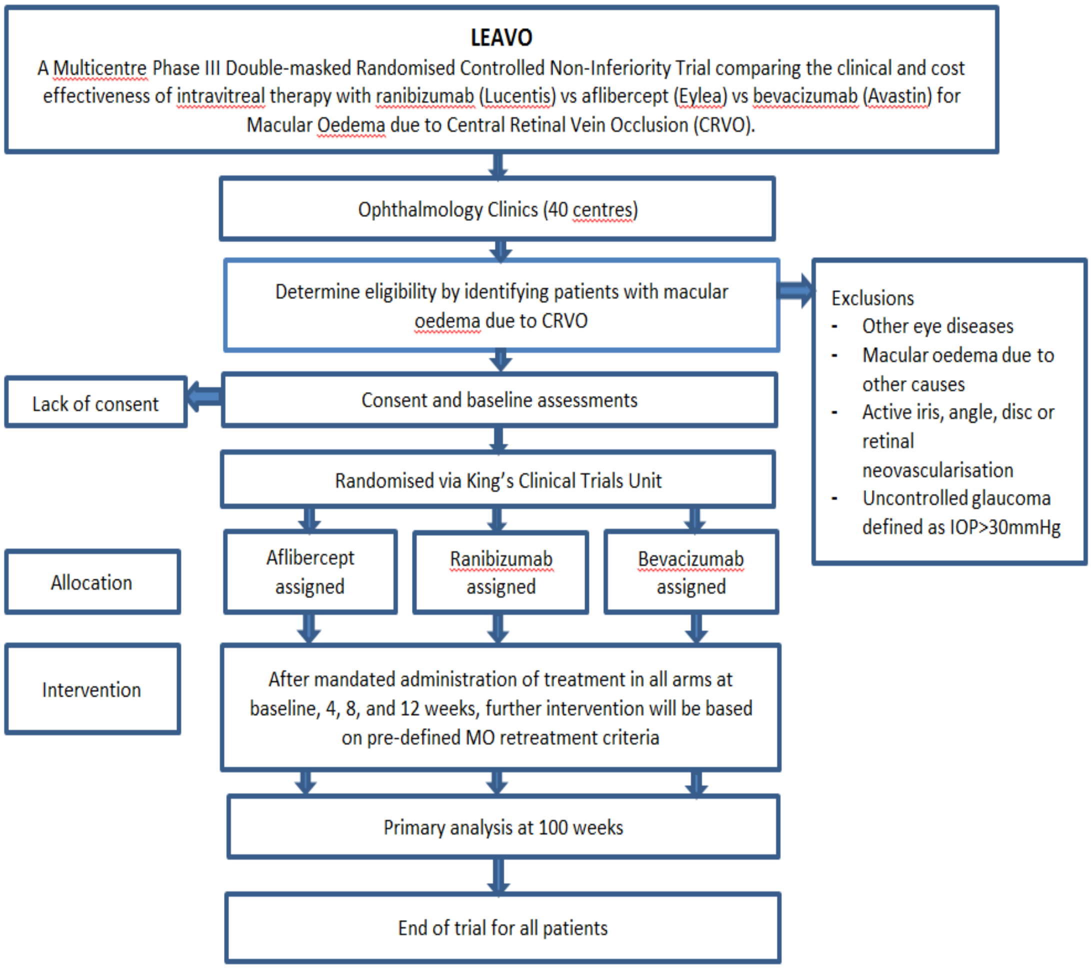

11.2 Baseline comparability of randomised groups

Baseline descriptions of participants by treatment and overall will be summarised (into Table 1 of the report). No significance testing will be carried out as any differences found may be chance-generated and not for hypothesised reasons.

Continuous variables such as OCT central subfield thickness and VFQ-25 will be summarised using means and standard deviations (SD) and/or medians and interquartile range (IQR) for variables presenting a skewed distribution. Categorical variables such as proportion of patients gaining ≥15 BCVA or participants with OCT CST < 320µm will be described using numbers and percentages.

11.3 Comparison of rates of adherence and follow-up

High compliance and low attrition rates are anticipated for this study according to previous clinical trial experience (91.6% of subjects completed the active treatment arms in the 12 month CRUISE (CRVO) study and withdrawals were mainly due to physician and patients decisions (see Protocol). A cumulative drop-out of approximately 15% by year 2 was predicted and reflected in the sample size calculations. Nevertheless, compliance rates and attrition rates will be compared and reported by arm using Fisher’s exact test.

12.0 Analysis covariates

12.1 Stratifiers

It is important to consider which, if any, covariates are to be adjusted for in the analyses. The ICH E9 guideline (1) recommends that consideration be given to accounting for randomisation stratifiers by adjusting for them as covariates in linear model. This tends to improve the precision of estimated treatment effects. Therefore, for continuous outcomes, the analysis will include adjustment for the randomisation stratifiers of screening BCVA letter score (3 levels) and disease duration (2 levels).

12.2 Baseline

The corresponding baseline measure for a continuous outcome is also often predictive of the outcome at follow-up. Therefore “baseline”, if collected, will be an additional covariate when modelling continuous outcomes. This will be the case for visual acuity and macular volume. The continuous baseline will have precedence for inclusion in the model over the corresponding categorical randomisation stratifier, where applicable.

13.0 Primary outcome analysis

13.1 Statistical Model

The following description of the statistical analysis applies to each of the two investigational treatments, bevacizumab and afilbercept and the standard treatment, ranibizumab.
The primary efficacy measure is the change from baseline in refracted best corrected visual acuity (BCVA) in the study eye, using the ETDRS letter score at 100 weeks. As the analysis approach for continuous outcomes below makes advantage of covariate-adjustment for the baseline of the outcome, the primary endpoint can equivalently be regarded to be each participant’s 100-week measurement. This is convenient because then those with a 100-week outcome, but whose baseline measurement is missing, are not regarded to be missing the endpoint. The primary outcome may therefore be referred to below as the 100-week visual acuity, rather than the change in this from baseline to 100 weeks.

The primary outcome will be analysed using a linear mixed effects (LME) model incorporating the 5 post-baseline measurements of the refracted BCVA outcome ” (12, 24, 52, 76 and 100 weeks). This mixed model will have, by definition a mix of random and fixed effect terms. The random effect in the model will be *participant*, represented as a random intercept at each follow-up timepoint, with allowance for within-participant correlation in the

adjusted post-baseline outcomes. The fixed effects in the model will be the main effect terms for arm, the two stratifiers: visual acuityand disease duration, "time", the baseline of the outcome and its missing indicator required for the missing indicator method (21). The other fixed effects to be included in the model will be the interactions between "*time*" and each of the other fixed effects in the model. This model allows the treatment effect to be formally tested at 52 weeks, at the primary timepoint of 100 weeks, and estimated at 24 and 76 weeks.

13.2 Intention to Treat Strategy

Outcome data will be valid and included if the BCVA measure is refracted. All randomised subjects who provide at least one post-baseline valid measurement will be included.

13.3 Per Protocol analysis

For the analysis of the primary outcome, the mixed effects model will be re-fitted in a reduced per protocol (PP) population already described in section 3.3.2.. Only valid (refracted) measurements will be included, and so the per protocol analysis will be a subset of the outcome measurements in the 52 and 100-week ITT analysis LME model.

13.4 Concluding non-inferiority

Non-inferiority will only be concluded if this is declared by both the ITT analysis and the PP analysis at 100 weeks. Non-inferiority will also be assessed secondarily in ITT and PP populations at 52 weeks from the same models. Non-inferiority will be declared if the estimated 95% confidence interval for the difference in means lies wholly above the margin of -5 letters in both ITT and PP analysis models primarily at 100 weeks and secondarily at 52 weeks.

13.5 Superiority

If non-inferiority is concluded, superiority will be assessed from the ITT LME model by reporting the *p*-value from the two-sided test of the hypothesis of a zero difference in population means using a 5% significance level without need for correction for multiple testing.

In addition, if both investigative treatments were considered non-inferior to the standard treatment at 100 weeks then superiority of the investigative treatments will be assessed to each other.

13.6 Subgroup analysis

The threesubgroup variables will be assessed by extending the primary outcome model to have an interaction between arm and each categorical subgroup variable. Subgroup variables with more than two categories that are ordinal will be entered as linear in the interaction. The treatment effects will also be presented within each subgroup category with a 95% confidence interval.

13.7 Sensitivity to missing data

An expert missing-data group concluded that rather than statisticians reacting to missing data at the end of a trial, there should be comprehensive, proactive planning for handling missing data at the stage of designing trials (22). The group recommended there should be consideration of missing data mechanisms (e.g Missing At Random), and, if the missing data may be informative that appropriate sensitivity analyses should be undertaken to investigate the robustness of the inferences to the different assumptions made by the main analysis. It has also been recommended that analyses allowing for non-response and low intervention uptake (or compliance) are best specified in advance and included in the analysis plan (23). As it is expected that compliance will be high from the fear of loss of sight, and as non-inferiority is concluded only when declared in both a compliant PP population and a less compliant ITT population, the focus is on handling of missing data.

A sensitivity analysis will be undertaken to assess the possibility of alternative plausible values of treatment effect arising from potential mishandling of missing data in the primary analysis model.

The LME model for the primary outcome analysis described above is the first of a two-part approach called the Intention to Treat Strategy (13) in which a second analysis examines the sensitivity of the results to missing data in the full randomised, Intention to Treat, population. This meets the ideal of ITT. The approach to missing data taken for Leavo follows the recently published implementation paper of the ITT strategy (24). This is then also applied again to the PP population so that the non-inferiority conclusion can be re-assessed under the sensitivity analysis.

For the sensitivity analysis, we pre-specify a range for best visual acuity from -20 letters to +20 letters over which the mean of the "unobserved outcome data" might *depart* (or be different) from the mean of the "observed outcome data" (24). In other words, this range can be thought of as how much a typical subject with missing data may on average have had a different estimated treatment effect compared to the corresponding subject with the outcome data observed (given the same baseline covariates and follow-up data in the LME model). The range (-20 to +20) is chosen to represent both negative and positive *departures* that could potentially arise as the "net effect" of alternative reasons which may be unknown; such as dropout due to no anticipated further improvement, or dropout due to no improvement so far together with no anticipated achievable improvement.

This range of 40 letters (from -20 to +20) is generously wide for exploring sensitivity of the main results to departures from the MAR assumption, because 20 letters (as the maximum *departure* in either direction) is larger than the detectable between-arm treatment effect of 3 lines (15 letters) seen in superiority trials (difference in means) which is a sizeable shift in the mean of the distribution for dropouts compared to completers.

At the end of the trial, the fractions of individuals with missing data for visual acuity at 100 weeks will be available in each arm f_i (for intervention) and f_c (for control). The parameter representing excess visual acuity in those missing compared to those observed, *δ, will take values by passing across the range* -20 to +20. Three scenarios will be undertaken within the sensitivity analysis (23, 24). These reflect whether departures from the MAR assumption apply within the intervention arms only (aflibercept and bevacizumab), within the control arm only (ranibizumab), or within both arms equally and in the same direction (thereby

potentially cancelling out across the sensitivity range, if the dropout rate were to be the same in both arms).

Scenario 1: the treatment effect from the LME model will be increased by $f_i\delta$
Scenario 2: the treatment effect from the LME model will be increased by $-f_c\delta$
Scenario 3: the treatment effect from the LME model will be increased by $(f_i-f_c)\delta$

13.8 Sensitivity analysis to use of concomitant treatments

The use of concomitant treatments will be monitored by the DMC. If necessary, a sensitivity analysis will be undertaken to examine the robustness of the 100-week per protocol analysis to the use of concomitant treatments.

13.9 Interim analysis

Formal interim analysis of the primary outcome for early stopping is not planned for this study. Regular interim reports will be prepared as needed for DMEC meetings.

14.0 Secondary outcome analysis

14.1 Analysis of continuous outcomes

As for the primary outcome, the analysis of continuous secondary outcomes will be compared between arms at 100-weeks using linear mixed effect model adjusting for all randomisation stratifiers and where collected, the baseline of the outcome with the associated missing indicator. Time will be represented as categorical contrasts in main effect form and in interaction with all other fixed effects.

14.2 Analysis of binary outcomes

For the binary outcomes, such as the proportion of participants with ≥15 ETDRS letter improvement, chi-squared tests will be used. Safety outcomes will be reported as unadjusted patient proportions and rates within and between arms with 95% confidence intervals using exact methods where appropriate.

14.3 Analysis methods for secondary outcomes

All study analyses will be based on tests that are two-sided, including the two-sided 95% confidence intervals.

For the secondary outcomes mentioned in section 6.2, the following analysis will be used:

Types of variables	Outcomes:	Methods:
Continuous	Best Corrected Visual Acuity at 52 weeks	Linear mixed effects model
	Mean OCT central subfield thickness (CST) at 52 and 100 weeks	Linear mixed effects model
	Macular volume at 52 and 100 weeks	Linear mixed effects model
	VFQ25 composite score, distance and near subscales at 52 and 100 weeks	Linear mixed effects model
	EQ-5D with and without vision bolt-on at 52 and 100 weeks	Linear mixed effects model
	Number of injections by 100 weeks	Difference in means
	Change in retinal non-perfusion at week 100 as assessed by two methods in different sites:<br>i)Disc area of non-perfusion (in approx. 27 sites)<br>ii)Ischaemic index (in approx. 13 sites)	ANCOVA (for each assessment method) and Fisher's method of combining p-values
Categorical	Participants with ≥15 and ≥ 10 ETDRS letter improvement, <15 letter loss and ≥ 30 ETDRS letter loss (severe visual loss) at 52 and 100 weeks	Chi-squared tests
	Participants with ≥73 ETDRS letters or better, ≤ 58 ETDRS letter and ≤19 letters at 52 and 100 weeks	Chi-squared tests
	Participants with OCT CST <320μm at 52 and 100 weeks	Chi-squared tests
	Persistent non-responders participants at 52 and 100 weeks	Chi-squared tests
	Participants that develop ocular neovascularisation at 52 and 100 weeks	Chi-squared tests
	Participants with OCT anatomical features: diffuse intraretinal oedema, intraretinal cystic change, subretinal fluid, vitreomacular interface abnormaility (either VMT or ERM) at 52 and 100	Chi-squared tests
	Prevalence of local and systemic side effects	Fisher's exact test

15.0 Handling multiple comparisons

Significance tests will be used sparingly and restricted where possible to addressing stated hypotheses. Secondary outcomes, as well as the primary outcome, will be summarised using an effect size with a 95% confidence interval. Interpretation for those secondary outcomes that do not directly address the stated study hypotheses will be more cautious.

16.0 Software

Data management:
An online data collection system for clinical trials (MACRO; InferMed Ltd) will be used. This is hosted on a dedicated server at KCL and managed by the MH&N CTU. The MH&N CTU Data Manager will extract data periodically as needed and provide these in comma sepa (.csv) format.

Statistical analysis:
The principal software package will be IBM SPSS Statistics 23 and R software will be available.

17.0 DMC monitoring

We expect the DMEC would want to monitor the non-inferiority of the investigational treatments in relation to the standard treatment and we would regularly provide information such as non-compliance and withdrawal and other information listed on appendix 1.

18.0 Acknowledgments

In translating the study protocol into this statistical analysis plan, we are grateful to explanations from the study team including Philip Hykin and Sobha Sivaprasad. Further versions of the plan will be commented on by members of the Data Monitoring and Trial Steering Committees.

19.0 Amendments to Versions

Version 1 was written by Joana Vasconcelos on 4th November 2013. Version 1.1 was re-written on 16th April 2014 to take into account the study protocol version 1.27.

Professor Toby Prevost verified the first version, leading to Version 2 on 21st April 2014.

Version 3 was produced on 22nd April 2014 after comments from the Chief Investigator, Phil Hykin and the co-lead investigator, Sobha Sivaprasad.

Version 4 was produced on 12th September 2014 which accounted for comments made by the DMC chair/statistician and protocol version 2.2.

Version 5 was produced on 30th September 2014 and updated on 26th November 2014 to take into account LEAVO protocol v3.0. This will be the final version approved by the independent TSC after their comments.

Amendments to versions will be listed here.

Version 5.1 was amended to Version 5.2 as a result of the DMC meeting held on 11th December, in open session, and the DMC recommendation to the TSC, discussed at the TSC meeting on 8th January 2016.

The DMC discussed the circumstances under which a BCVA score at 100 weeks, or other timepoints, would not reflect the underlying visual status of a participant. In particular, recent vitreous haemorrhage~~s~~ may cause low BCVA scores which would then return to normal for the patient, either spontaneously or through appropriate clinical management (vitrectomy). The challenge is that any such measurements could artificially induce very large negative changes in BCVA which would have enormous influence in statistical analysis – specifically by leading to very large inflations in the standard deviation for the change from baseline. This could have profound implications for the ability of this non-inferiority trial to achieve its objectives, which rely on the 95% confidence interval for the difference between randomised groups in the change from baseline falling within pre-specified bounds (the non-inferiority margin). As such values intrinsically do not reflect the underlying visual status of the patient, the DMC proposed that the TSC consider amending the primary analysis population measurements to exclude from analysis any refracted BCVA measurement which is both >3 SD below the mean at that timepoint (including all measurements) and taken within 3 months of occurrences of a vitreous haemorrhage. The TSC also considered a proposal from the PI that Visual Acuity loss due to other casues unrelated to maculopathy secondary to CRVO be included. The absolute number of measurements excluded across the timepoints of measurement of refracted visual acuity is expected to be small. The TSC requested confirmation that these occurrences (number and nature) will be transparently reported by arm, and this has been included in this SAP.

Version 5.2 was amended to Version 5.3 as a result of the DMC meeting held on 1st November 2016. The inclusion/exclusion criteria and the Per Protocol definition was updated to be in conformity with the Protocol version 4.0 as well as the wording of the secondary objectives. The randomisation stratifier 'previous treatment' was removed from the outcome analysis models as a covariate as well as a variable in subgroup analysis due to the very small number of patients having had previous treatment in the trial. Also the categories 3-6 months and >6months of the disease duration stratifier will be merged for the same reasons and will be analysed as such in the models and subgroup analysis. Finally, the method of randomisation had been mis-typed in on section 8.2 in the SAP as being stratified, whereas it has all along been minimisation. This wording has been corrected.

Reference list

1. International conference on harmonisation; guidance on statistical principles for clinical trials; availability--FDA. Notice. *Fed Regist*. 1998;63(179):49583-98.
2. Schulz KF, Altman DG, Moher D, Group C. CONSORT 2010 statement: updated guidelines for reporting parallel group randomised trials. *BMJ*. 2010;340:c332.
3. Committee for Proprietary Medicinal P. Committee for Proprietary Medicinal Products (CPMP): points to consider on adjustment for baseline covariates. *Stat Med*. 2004;23(5):701-9.
4. Guidance for Industry Non-Inferiority Clinical Trials.
5. Committee for Proprietary Medicinal P. Points to consider on switching between superiority and non-inferiority. *Br J Clin Pharmacol*. 2001;52(3):223-8.
6. Garrett AD. Therapeutic equivalence: fallacies and falsification. *Stat Med*. 2003;22(5):741-62.
7. Lasaffre E. Superiority, Equivalence and Non-Inferiority Trials. *Bull NYU Hosp Jt Dis*. 2008;66(2):150-42008.
8. Patient.co.uk: Egton Medical Information Systems Limited; [cited 2014]. Available from: http://www.patient.co.uk/.
9. MedlinePlus.
10. Investigators IS, Chakravarthy U, Harding SP, Rogers CA, Downes SM, Lotery AJ, et al. Ranibizumab versus bevacizumab to treat neovascular age-related macular degeneration: one-year findings from the IVAN randomized trial. *Ophthalmology*. 2012;119(7):1399-411.
11. Comparison of Age-related Macular Degeneration Treatments Trials Research G, Martin DF, Maguire MG, Fine SL, Ying GS, Jaffe GJ, et al. Ranibizumab and bevacizumab for treatment of neovascular age-related macular degeneration: two-year results. *Ophthalmology*. 2012;119(7):1388-98.
12. NICE FAD: Ranibizumab for treating visual impairment caused by macular oedema secondary to retinal vein occlusion 2013 [27-05-13].
13. White IR, Horton NJ, Carpenter J, Pocock SJ. Strategy for intention to treat analysis in randomised trials with missing outcome data. *BMJ*. 2011;342:d40.
14. D'Agostino RB, Sr., Massaro JM, Sullivan LM. Non-inferiority trials: design concepts and issues - the encounters of academic consultants in statistics. *Stat Med*. 2003;22(2):169-86.
15. Campochiaro PA, Brown DM, Awh CC, Lee SY, Gray S, Saroj N, et al. Sustained benefits from ranibizumab for macular edema following central retinal vein occlusion: twelve-month outcomes of a phase III study. *Ophthalmology*. 2011;118(10):2041-9.
16. Mangione CM, Lee PP, Gutierrez PR, Spritzer K, Berry S, Hays RD. Development of the 25-item National Eye Institute Visual Function Questionnaire. *Arch Ophthalmol*. 2001;119(7):1050-8.
17. Janssen MF, Birnie E, Bonsel GJ. Quantification of the level descriptors for the standard EQ-5D three-level system and a five-level version according to two methods. *Qual Life Res*. 2008;17(3):463-73.
18. van Hout B, Janssen MF, Feng YS, Kohlmann T, Busschbach J, Golicki D, et al. Interim scoring for the EQ-5D-5L: mapping the EQ-5D-5L to EQ-5D-3L value sets. *Value Health*. 2012;15(5):708-15.
19. Longworth L, Yang Y, Young T, Mulhern B, Hernandez Alava M, Mukuria C, et al. Use of generic and condition-specific measures of health-related quality of life in NICE decision-making: a systematic review, statistical modelling and survey. *Health Technol Assess*. 2014;18(9):1-224.

20. Carpenter J, Bithell J. Bootstrap confidence intervals: when, which, what? A practical guide for medical statisticians. *Stat Med*. 2000;19(9):1141-64.
21. White IR, Thompson SG. Adjusting for partially missing baseline measurements in randomized trials. *Stat Med*. 2005;24(7):993-1007.
22. Burzykowski T, Carpenter J, Coens C, Evans D, France L, Kenward M, et al. Missing data: discussion points from the PSI missing data expert group. *Pharm Stat*. 2010;9(4):288-97.
23. White IR, Kalaitzaki E, Thompson SG. Allowing for missing outcome data and incomplete uptake of randomised interventions, with application to an Internet-based alcohol trial. *Stat Med*. 2011;30(27):3192-207.
24. White IR, Carpenter J, Horton NJ. Including all individuals is not enough: lessons for intention-to-treat analysis. *Clin Trials*. 2012;9(4):396-407.

Appendix I – Lists of Tables for DMC and for main trial

These are draft tables for the DMC meetings.

1) **Recruitment by calendar month**

Months:	Site 1	Site 2	Site 3	(…)	Site 40
Dec 2014<br>Jan 2015<br>Feb 2015<br>…<br>Total					

2) **Randomisation -** Stratifiers

	Arm Ia/ Active N (% total)	Arm Ib/ Active N (% total)	Arm C/ Control N (% total)	Total
**BCVA letter score**<br>≤38<br>39-58<br>≥59				
**On onset of symptoms to presentation at hospital and commencement of therapy**<br><3 months<br>3-6 months<br>>6 months				
**Treatment:**<br>Naïve<br>Previous				
**Overall**				

3) **Compliance**

i) **Up to and including 12 weeks of treatment**

Weeks:	Arm Ia/ Active % (n/d)	Arm Ib/ Active % (n/d)	Arm C/ Control % (n/d)
4<br>8<br>12<br>**Total**			

n: Total number of patients compliant; d: Total no patients (not having withdrawn from the trial)

ii) Remaining weeks

Weeks:	Arm Ia/ Active % (n/d)	Arm Ib/ Active % (n/d)	Arm C/ Control % (n/d)
24<br>52<br>76<br>100<br>**Total**			

n: Total number of patients compliant; d: Total no patients (not having withdrawn from the trial)

4) Outcomes

Primary outcome - Visual acuity using the ETDRS letter scoring tool

Weeks:	Arm Ia/ Active Mean (SD)	Arm Ib/ Active Mean (SD)	Arm C/ Control Mean (SD)	Ia vs C z-value	Ia vs C 95% CI	Ib vs C z-value	Ib vs C 95% CI
24<br>52<br>76<br>100							

Secondary outcomes:

Outcomes:	Arm Ia/ Active	Arm Ib/ Active	Arm C/ Control	Ia vs C z-value	Ib vs C z-value
Change in mean OCT central subfield thickness (CST) at 52 and 100 weeks.	Mean (SD)	Mean (SD)	Mean (SD)		
Change in macular volume at 52 and 100 weeks	Mean (SD)	Mean (SD)	Mean (SD)		
Participants with ≥15 ETDRS letter improvement, ≥ 10 letter improvement, <15 letter loss and ≥ 30 ETDRS letter loss at 52 and 100 weeks.	% (n)	% (n)	% (n)		
Participants with ≥73 ETDRS letters or better, ≤ 58 ETDRS letter and ≤19 letters at 52 and 100 weeks.	% (n)	% (n)	% (n)		

Note: These will be extended and modified by the DMC.

The denominators will either be presented here or in a "Completeness of Data" Table where observed denominators are compared to expected denominators.

Appendix II – Record of data decisions during the blinded review.

21.1 Record of data decisions

During the blinded review data decisions will be recorded here.

21.2 Record of analysis decisions

During the blinded review data decisions will be recorded here.

Appendix III – Record of data decisions after the blind-break

Record of analysis decisions

i) There were some patients that were randomised using the wrong stratification category after looking at the actual continuous values at baseline. Therefore, the actual categorised baseline values of stratifying covariates, rather than those used in the randomisation which included errors, will be used in the outcome models, so that any baseline confounding by these is more fully adjusted for, and analyses are consistent with subgroup analyses using the same categorisations of these covariates. There was agreement for this approach as the trial employs minimisation. The DMC Chair approved this decision on 13th February 2019.

ii) There were three participants with BCVA scores missing at baseline due to not having completed the one meter test despite the four meter test being less than 20. These were:

Participant 11014 who had a score of 19 in the four meter test.
Participant 16171 who had a score of 17 in the four meter test.
Participant 10368 who had a score of 4 in the four meter test.

According to the eligibility criteria, which requires BCVA to be >=19, participant 11014 was in fact eligible but not necessarily the other two, who despite being randomised, have not met proof of eligibility beyond doubt as would be preferred for a per protocol population. Therefore, for the PP analysis P11014 will be included (using the missing indicator method as planned), P16171 and P10368 will be excluded, and a sensitivity analysis for the main Per Protocol analysis at 100 weeks will be carried out as follows:

a) Participant 16171 will be included in the PP population but not participant 10368.
b) Participant 10368 will be included in the PP population but not participant 16171
c) Both participants (16171 and 10368) will be included in the PP population.

This decision was agreed among statisticians on 27th February 2019 and approved by the Chief investigator on 1st March 2019.

Signatures

Trial Statistician 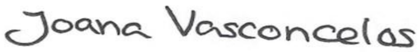

Joana Vasconcelos ______________________________ 1/3/2019____________
Signature Date

Signatures

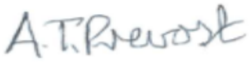

Senior Statistician
Prof. Toby Prevost ____________________________1/3/2019______
Signature Date

Signatures

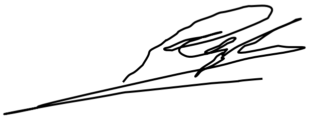

Chief Investigator

Mr Philip Hykin ________________________________1/3/2019___

Signature Date

Signatures

DMC Chair

Prof. Sarah Walker ______________________1/3/2019______
Signature Date

TSC Chair

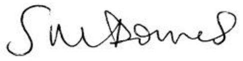

Prof Susan Downes

Signature Date 3/5/2019
