# Supplementary material for: Clinical Effectiveness of Intravitreal Therapy With Ranibizumab vs Aflibercept vs Bevacizumab for Macular Edema Secondary to Central Retinal Vein Occlusion: A Randomized Clinical Trial
Source: JAMA Ophthalmol. 2019 Aug 29;137(11):1256–64. doi: 10.1001/jamaophthalmol.2019.3305 (PMC6865295; doi:10.1001/jamaophthalmol.2019.3305)
Supplement: Supplement 2. — eTable 1. The LEAVO Study: Eligibility Criteria eTable 2. ITT and PP Analyses for the Primary Outcome at 100 Weeks eTable 3. Categorical Visual Acuity Outcomes by Group eTable 4. Ocular Adverse Events and APTC Events eFigure 1. Sensitivity Analyses for the Missing at Random Assumption in the Primary Outcome Analysis Assessing Noninferiority of Aflibercept and for the Missing at Random Assumption in the Primary Outcome Analysis Assessing Noninferiority of Bevacizumab eFigure 2. Percentage of Patients With ≥15 ETDRS Letter Improvement at 52 and 100 Weeks eFigure 3. Percentage of Patients With OCT<320 μm at 24, 52, and 100 Weeks [file jamaophthalmol-137-1256-s002.pdf]

# Supplementary Online Content

Hykin P, Prevost AT, Vasconcelos JC, et al; LEAVO Study Group. Clinical effectiveness of intravitreal therapy with ranibizumab vs aflibercept vs bevacizumab for macular edema secondary to central retinal vein occlusion: a randomized clinical trial. *JAMA Opthalmol*. Published online August 29, 2019. doi:10.1001/jamaophthalmol.2019.3305

**eTable 1.** The LEAVO Study: Eligibility Criteria

**eTable 2.** ITT and PP Analyses for the Primary Outcome at 100 Weeks

**eTable 3.** Categorical Visual Acuity Outcomes by Group

**eTable 4.** Ocular Adverse Events and APTC Events

**eFigure 1.** Sensitivity Analyses for the Missing at Random Assumption in the Primary Outcome Analysis Assessing Noninferiority of Aflibercept and for the Missing at Random Assumption in the Primary Outcome Analysis Assessing Noninferiority of Bevacizumab

**eFigure 2.** Percentage of Patients With ≥15 ETDRS Letter Improvement at 52 and 100 Weeks

**eFigure 3.** Percentage of Patients With OCT<320 µm at 24, 52, and 100 Weeks

This supplementary material has been provided by the authors to give readers additional information about their work.

© 2019 Hykin P et al. *JAMA Ophthalmology*.

**eTable 1.** The LEAVO Study: Eligibility Criteria

**Inclusion Criteria**

1. Subjects of either sex aged ≥ 18 years.
2. Clinical diagnosis of centre-involving macular oedema (MO) due to central retinal vein occlusion (CRVO)
3. CRVO of ≤ 12 months duration
4. . Best corrected visual acuity (BCVA) Early Treatment Diabetic Retinopathy Study (ETDRS) letter score (approximate Snellen equivalent) in the study eye between 78 (20/32) and 19 (20/400).
5. Optical Coherence Tomography (OCT) central subfield thickness > 320μm (Spectralis, Heidelberg) [or equivalent for alternative OCT device] predominantly due to MO secondary to CRVO in the study eye.
6. Media clarity, pupillary dilatation and subject cooperation sufficient for adequate fundus imaging of the study eye.
7. BCVA ETDRS letter score (approximate Snellen equivalent) in the non-study eye ≥ 14 (20/600).

**Exclusion Criteria**

1. Macular oedema considered to be due to a cause other than CRVO (e.g. Irvine-Gass syndrome).
2. An ocular condition is present that, in the opinion of the investigator, might affect macular oedema or alter visual acuity during the course of the study (e.g. vitreomacular traction).
3. Any diabetic retinopathy or diabetic macular oedema at baseline clinical examination of the study eye.
4. Moderate or severe non proliferative diabetic retinopathy (NPDR) or quiescent, treated or active proliferative diabetic retinopathy (PDR) or macular oedema in the non-study eye. Note: Mild NPDR only is permissible in the non-study eye.
5. History of treatment for MO due to CRVO in the past 90 days with intravitreal or peribulbar corticosteroids or in the last 60 days with anti- vascular endothelial growth factor (anti-VEGF) or more than 6 prior anti-VEGF treatments* in the previous 12 months.
6. Active iris or angle neovascularisation, neovascular glaucoma, untreated disc neovascularisation (NVD), retinal neovascularisation elsewhere (NVE) and vitreous haemorrhage or treatment for these conditions in the last 1 month*.
7. Uncontrolled glaucoma [>30mmHg], either untreated or on anti-glaucoma medication at screening.
8. Any active periocular or intraocular infection or inflammation (e.g. conjunctivitis, keratitis, scleritis, uveitis, endophthalmitis).
9. In cases of bilateral CRVO, if both eyes are potentially eligible, unless the patient prefers otherwise the worst-seeing eye will be recruited.

**Systemic exclusion criteria**

1. Uncontrolled blood pressure defined as a systolic value > 170mmHg and diastolic value > 110mmHg.
2. Myocardial infarction, stroke, transient ischaemic attack, acute congestive cardiac failure or any acute coronary event less than 3 months before randomisation
3. Women of child bearing potential unless using effective methods of contraception throughout the study and for 6 months after their last injection for the trial. Effective contraception is defined as one of the following:
    (a) Barrier method: condoms or occlusive cap with spermicides.
    (b) True abstinence: When it is in line with the preferred and usual lifestyle of the subject.
    (c) Have had tubal ligation or bilateral oophorectomy (with or without hysterectomy).
    (d) Male partner sterilisation: the vasectomised male partner should be the only partner for the female participant.
    (e) Use of established oral, injected or implanted hormonal methods of contraception and intrauterine device.
4. Pregnant or lactating women.
5. Males who do not agree to an effective form of contraception for the duration of the study and for 6 months after their last injection for the trial.
6. Hypersensitivity to the active ingredients aflibercept, bevacizumab or ranibizumab or any of the excipients of these drugs.
7. Hypersensitivity to Chinese Hamster Ovary (CHO) cell products or other recombinant human or humanised antibodies.
8. A condition that, in the opinion of the investigator, would preclude participation in the study.
9. Participation in an investigational trial involving an investigational medicinal product within 90 days of randomization.

*post Protocol Amendment

© 2019 Hykin P et al. *JAMA Ophthalmology*.

**eTable 2.** ITT and PP Analyses for the Primary Outcome at 100 Weeks

| **Mean (SE§) BCVA at screening** | | **Mean (SE) (N) BCVA at 100 weeks** | | **Adjusted difference between groups (95% CI) at 100 weeks** | **p-value for non-inferiority (p<0.025 is significant)** | **p-value for superiority (p<0.05 is significant)** |
|---|---|---|---|---|---|---|
| **Aflibercept versus Ranibizumab ITT** | | | | | | |
| Aflibercept | Ranibizumab | Aflibercept | Ranibizumab | | | |
| 54.1 (1.2) | 53.6 (1.2) | 68.4 (1.6) (133) | 65.6 (1.7) (135) | 2.23 (-2.17, 6.63)*‡ | 0.0006 | 0.32 |
| **Aflibercept versus Ranibizumab PP** | | | | | | |
| Aflibercept | Ranibizumab | Aflibercept | Ranibizumab | | | |
| 55.0 (1.2) | 53.6 (1.3) | 69.5 (1.5) (128) | 65.7 (1.7) (133) | 3.49 (-0.91, 7.88)*† | <0.0001 | 0.12 |
| **Bevacizumab versus Ranibizumab ITT** | | | | | | |
| Bevacizumab | Ranibizumab | Bevacizumab | Ranibizumab | | | |
| 54.4 (1.1) | 53.6 (1.2) | 64.6 (1.8) (139) | 65.6 (1.7) (135) | -1.73 (-6.12, 2.67)‡ | 0.071 | 0.44 |
| **Bevacizumab versus Ranibizumab PP** | | | | | | |
| Bevacizumab | Ranibizumab | Bevacizumab | Ranibizumab | | | |
| 54.4 (1.2) | 53.6 (1.3) | 64.6 (1.8) (139) | 65.7 (1.7) (133) | -1.67 (-6.02, 2.68)† | 0.066 | 0.45 |

* Non-inferior relative to Ranibizumab.
**§ =** SE (standard error)
‡ The linear mixed-effects model incorporates 454 participants (n=148 ranibizumab, n=153 aflibercept and n=153 bevacizumab) with best corrected visual acuity at 100 weeks.
†The linear mixed-effects model incorporates 443 participants (n=145 ranibizumab, n=146 aflibercept and n=152 bevacizumab) with best corrected visual acuity at 100 weeks.

© 2019 Hykin P et al. *JAMA Ophthalmology*.

**eTable 3.** Categorical Visual Acuity Outcomes by Group

| **Outcomes** | **Ranibizumab % (n/N)** | **Aflibercept % (n/N)** | **Bevacizumab % (n/N)** | **Difference in proportions (95% CI) Aflibercept vs Ranibizumab** | **Difference in proportions (95% CI) Bevacizumab vs Ranibizumab** |
|---|---|---|---|---|---|
| Patients with ≥10 ETDRS letter improvement at 100 weeks | 63% (84/133) | 68% (90/132) | 63% (87/138) | 5% (-6.4, 16.4) | -0.1% (-11.6, 11.4) |
| Patients with <15 ETDRS letter decrease at 100 weeks | 90% (120/133) | 93% (123/132) | 90% (124/138) | 3% (-3.7, 9.6) | -0.4% (-7.5, 6.8) |
| Patients with ≥30 ETDRS letter decrease at 100 weeks | 5% (7/133) | 2% (3/132) | 6% (8/138) | -3% (-7.6, 1.6) | 0.5% (-4.9, 6.0) |
| Patients with ≥10 ETDRS letter improvement at 52 weeks | 60% (83/138) | 62% (86/138) | 64% (86/134) | 2.2% (-9.3, 13.7) | 4.0% (-7.5, 15.6) |
| Patients with <15 ETDRS letter decrease at 52 weeks | 90% (124/138) | 93% (129/138) | 95% (127/134) | 3.6% (-2.9, 10.1) | 4.9% (-1.4, 11.2) |
| Patients with ≥ 30 ETDRS letter decrease at 52 weeks | 6% (8/138) | 1% (2/138) | 3% (4/134) | -4.3% (-8.7, 0.03) | -2.8% (-7.7, 2.0) |
| Patients with >73 ETDRS letter score (>20/40 Snellen equivalent) at 100 weeks | 47% (63/135) | 44% (59/133) | 41% (57/139) | -2.3% (-14.2, 9.6) | -5.7% (-17.4, 6.1) |
| Patients with ≤58 ETDRS letter score (20/80) Snellen equivalent) at 100 weeks | 29% (39/135) | 20% (26/133) | 30% (42/139) | -9.3% (-19.5, 0.9) | 1.3% (-9.5, 12.1) |
| Patients with <19 ETDRS letter score (20/400 Snellen equivalent) at 100 weeks | 3% (4/135) | 2% (2/133) | 4% (6/139) | -1.5% (-5.0, 2.1) | 1.4% (-3.1, 5.8) |
| Patients with >73 ETDRS letter score (>20/40- Snellen equivalent) at 52 weeks. | 42% (59/139) | 42% (59/139) | 39% (53/135) | 0% (-11.6, 11.6) | -3.2% (-14.8, 8.4) |
| Patients with ≤58 ETDRS letter (≤20/80 Snellen equivalent) at 52 weeks | 28% (39/139) | 25% (35/139) | 24% (32/135) | -2.9% (-13.3, 7.5) | -4.4% (-14.7, 6.0) |
| Patients with <19letters ETDRS letter (20/400 Snellen equivalent) at 52 weeks | 4% (5/139) | 1% (2/139) | 4% (5/135) | -2.2% (-5.8, 1.5) | 0.1% (-4.3, 4.5) |

© 2019 Hykin P et al. *JAMA Ophthalmology*.

**eTable 4.** Ocular Adverse Events and APTC Events

| | Total (n=463) | Ranibizumab (n=155) | Aflibercept (n=154) | Bevacizumab (n=154) | Difference (95% CI) Aflibercept vs Ranibizumab | Difference (95% CI) Bevacizumab vs Ranibizumab |
|---|---|---|---|---|---|---|
| **Ocular adverse events** | | | | | | |
| Infectious endophthalmitis | 1 (0.2%) | 0 (0%) | 0 (0%) | 1 (0.6%) | 0.0% (-2.4% to 2.4%) | -0.6% (-3.6% to 1.8%) |
| Traumatic cataract | 0 (0%) | 0 (0%) | 0 (0%) | 0 (0%) | 0.0% (-2.4% to 2.4%) | 0.0% (-2.4% to 2.4%) |
| Retinal tear | 1 (0.2%) | 1 (0.6%) | 0 (0%) | 0 (0%) | -0.6% (-3.6% to 1.9%) | -0.6% (-3.6% to 1.9%) |
| Retinal detachment | 3 (0.6%) | 0 (0%) | 1 (0.6%) | 2 (1.3%) | 0.6% (-1.8% to 3.6%) | 1.3% (-1.3% to 4.6%) |
| Conversion to ischaemic CRVO | 25 (5.4%) | 8 (5.2%) | 10 (6.5%) | 7 (4.5%) | 1.3% (-4.2% to 7.0%) | -0.6% (-5.9% to 4.6%) |
| Anterior segment neovascularisation | 13 (2.8%) | 5 (3.2%) | 5 (3.2%) | 3 (1.9%) | 0.0% (-4.5% to 4.5%) | -1.3% (-5.6% to 2.8%) |
| Retinal Neovascularization | 6 (1.3%) | 1 (0.6%) | 4 (2.6%) | 1 (0.6%) | 2.0% (-1.4% to 5.9%) | 0.0% (-3.0% to 3.0%) |
| Vitreous haemorrhage | 6 (1.3%) | 0 (0%) | 2 (1.3%) | 4 (2.6%) | 1.3% (-1.3% to 4.6%) | 2.6% (-0.2% to 6.5%) |
| IOP elevation | 27 (5.8%) | 13 (8.4%) | 9 (5.8%) | 5 (3.2%) | -2.5% (-8.6% to 3.4%) | -5.1% (-10.9% to 0.2%) |
| **Systemic APTC events** | | | | | | |
| Cardiovascular – vascular deaths | 5 (1.1%) | 2 (1.3%) | 2 (1.3%) | 1 (0.6%) | 0.0% (-3.4% to 3.4%) | -0.6% (-4.0% to 2.4%) |
| Cardiovascular – non fatal MI | 2 (0.4%) | 0 (0%) | 0 (0%) | 2 (1.3%) | 0.0% (-2.4% to 2.4%) | 1.3% (-1.3% to 4.6%) |
| Cardiovascular – non fatal stroke | 6 (1.3%) | 2 (1.3%) | 4 (2.6%) | 0 (0%) | 1.3% (-2.4% to 5.3%) | -1.3% (-4.6% to 1.3%) |

© 2019 Hykin P et al. *JAMA Ophthalmology*.

**eFigure 1A.** Sensitivity Analysis for the Missing at Random Assumption in the Primary Outcome Analysis Assessing Noninferiority of Aflibercept

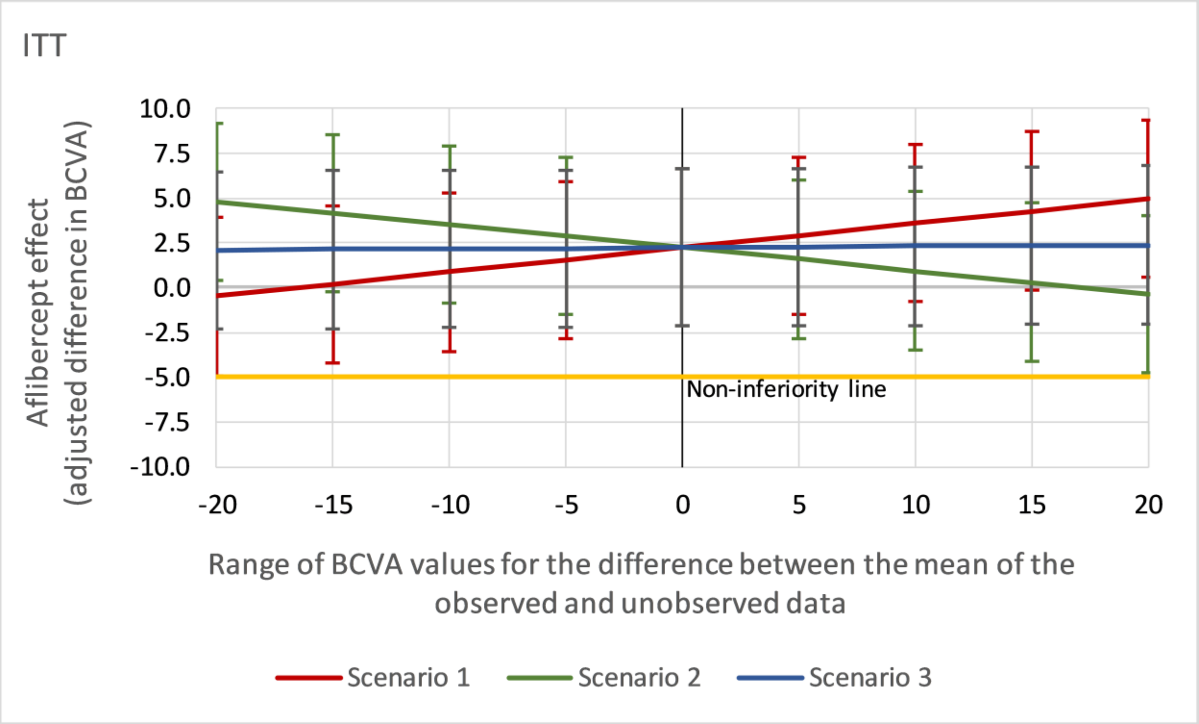


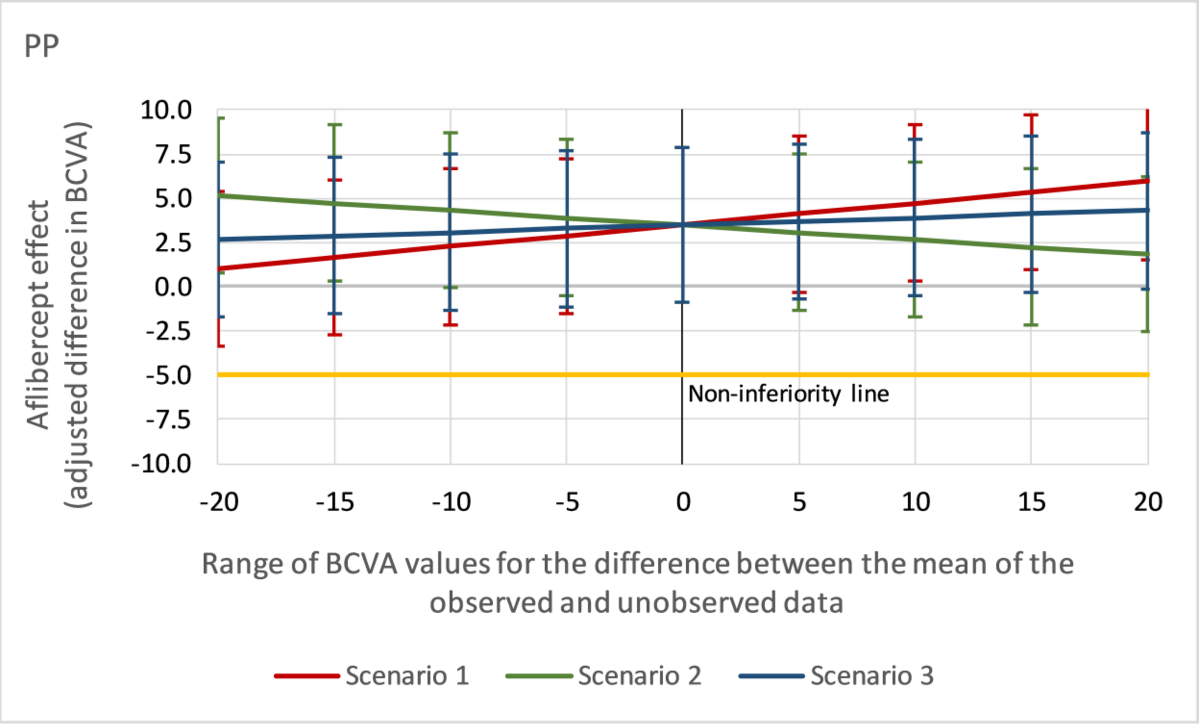


The sensitivity analysis assessed the impact on the treatment effect considering the mean outcome in those with unobserved data could range from minus 20 to plus 20 best corrected visual acuity (BCVA) letter score from patients with observed data (horizontal axis), in aflibercept patients only (Scenario 1), or in ranibizumab only (Scenario 2), or in both patient groups equally (Scenario 3). The treatment effect in the main analysis is shown at zero. Vertical bars are 95% CIs for the treatment effect. The 95% CI bars all lay above the non-inferiority margin of -5 confirming the non-inferiority of aflibercept in both intention-to -treat (ITT) and per protocol (PP) populations.

© 2019 Hykin P et al. *JAMA Ophthalmology*.

**eFigure 1B.** Sensitivity Analysis for the Missing at Random Assumption in the Primary Outcome Analysis Assessing Noninferiority of Bevacizumab

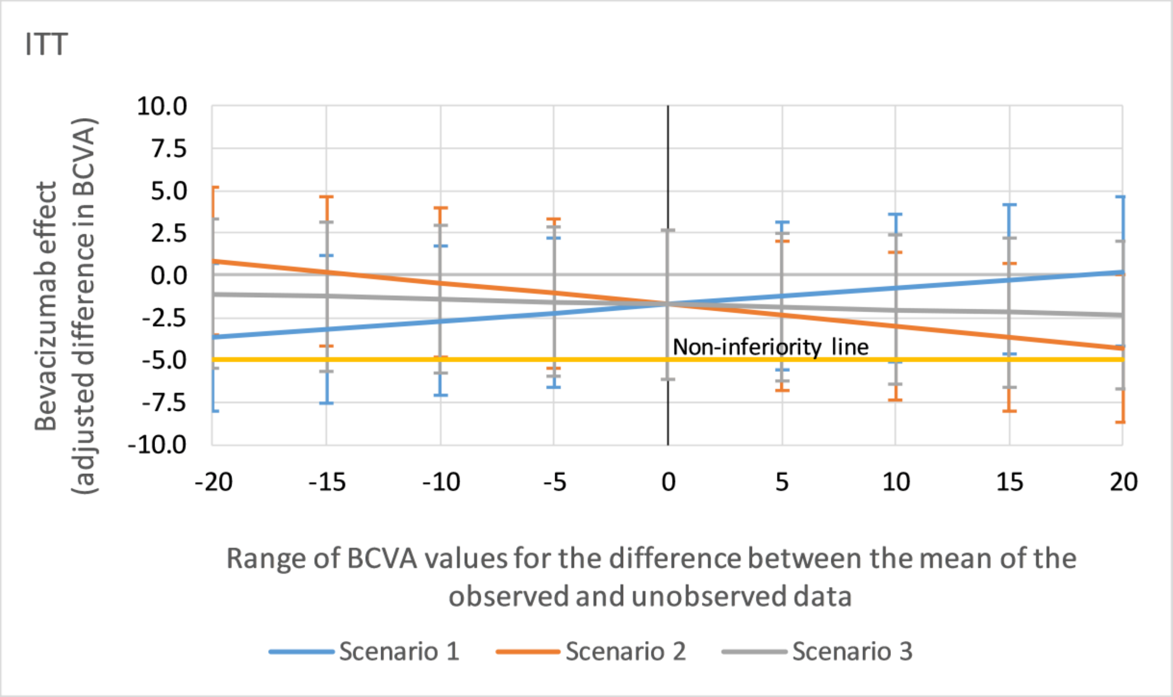


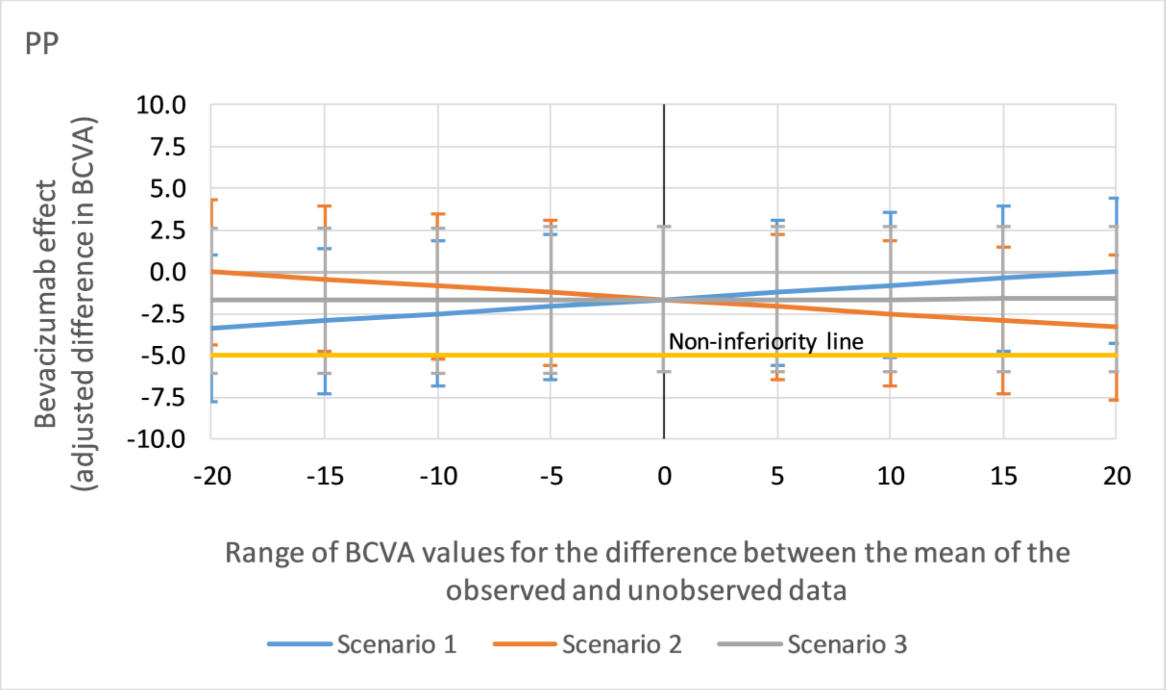


For scenario 3, and within most of the ranges of Scenarios 1 and 2, the lower confidence interval limit lay below the non-inferiority margin of -5, confirming the main analysis conclusion of a lack of non-inferiority of bevacizumab. The difference in mean between those with unobserved BCVA data and those with observed BCVA data would need to be assumed to be 12 letters higher for bevacizumab compared to ranibizumab in scenario 1 (or 12.4 letters higher in scenario 2), in order to change the main analysis conclusion of a lack of non-inferiority in both ITT and PP populations.

© 2019 Hykin P et al. *JAMA Ophthalmology*.

**eFigure 2.** Percentage of Patients With ≥ 15 ETDRS Letters Improvement at 52 and 100 Weeks

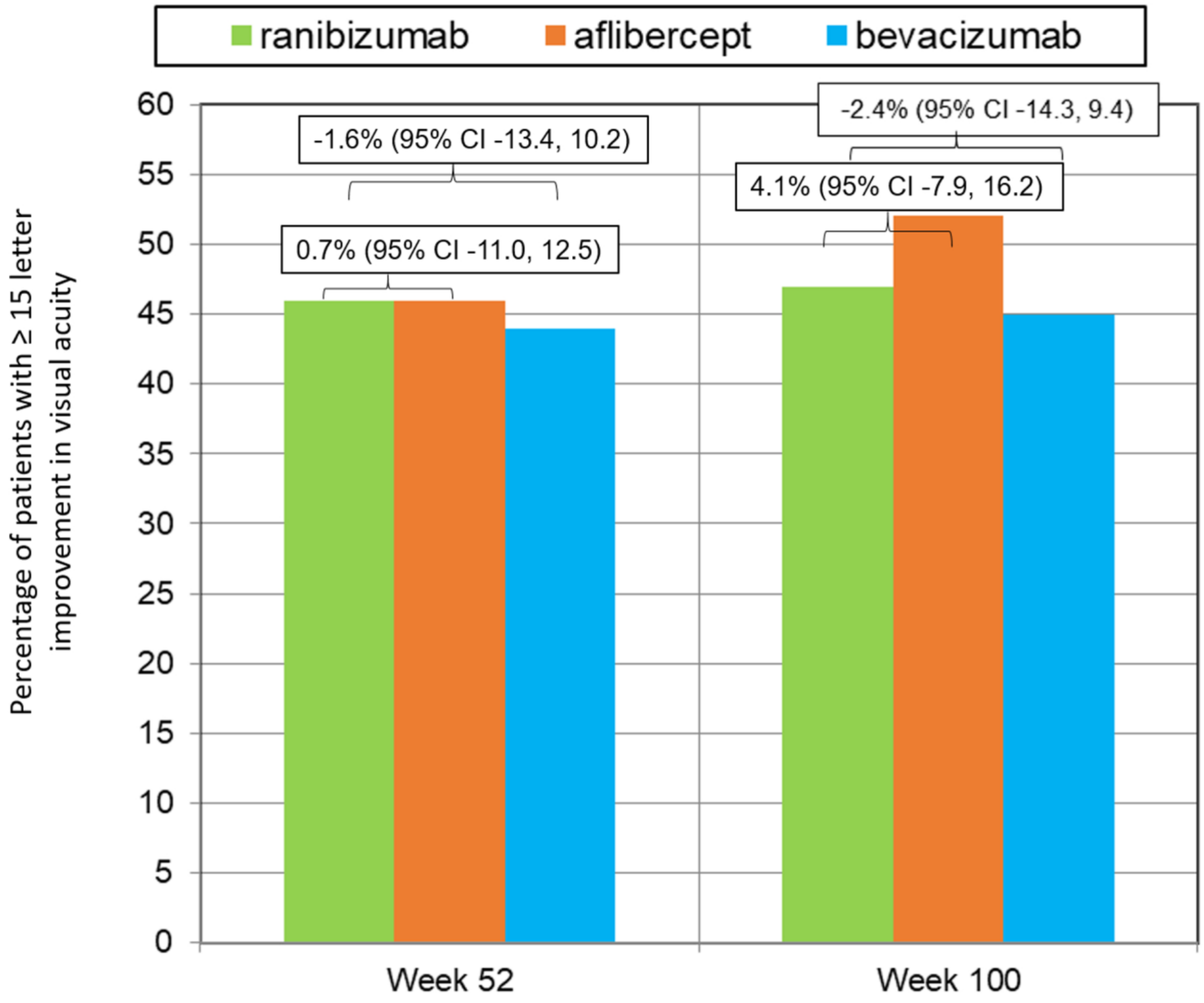


© 2019 Hykin P et al. *JAMA Ophthalmology*.

**eFigure 3.** Percentage of Patients With OCT < 320 µm at 24, 52, and 100 Weeks

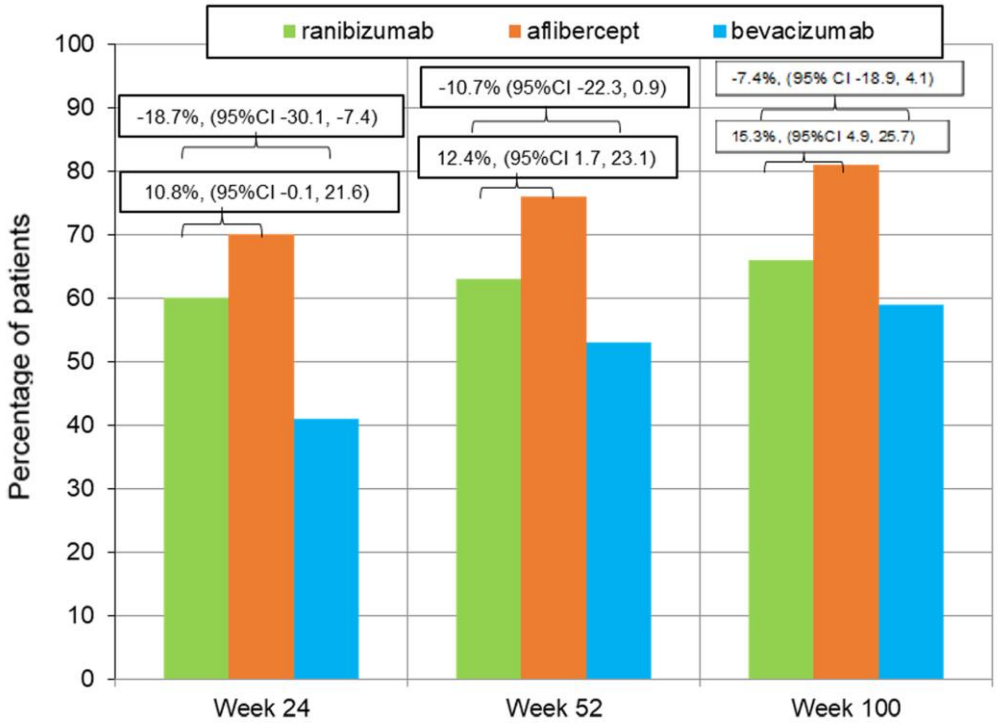


© 2019 Hykin P et al. *JAMA Ophthalmology*.
