# Supplementary material for: Clinical Effectiveness of Intravitreal Therapy With Ranibizumab vs Aflibercept vs Bevacizumab for Macular Edema Secondary to Central Retinal Vein Occlusion: A Randomized Clinical Trial
Source: JAMA Ophthalmol. 2019 Aug 29;137(11):1256–64. doi: 10.1001/jamaophthalmol.2019.3305 (PMC6865295; doi:10.1001/jamaophthalmol.2019.3305)
Supplement: Supplement 3. — Data Sharing Statement [file jamaophthalmol-137-1256-s003.pdf]

Data Sharing Statement

Hykin. Clinical Effectiveness of Intravitreal Therapy With Ranibizumab vs Aflibercept vs Bevacizumab for Macular Edema Secondary to Central Retinal Vein Occlusion. *JAMA Ophthalmol*. Published August 29, 2019. 10.1001/jamaophthalmol.2019.3305

Data
Data available: No

Additional Information
Explanation for why data not available: Data Sharing Agreement A Multicentre Phase III Double-masked Randomised Controlled Non-Inferiority Trial comparing the clinical and cost effectiveness of intravitreal therapy with ranibizumab (Lucentis) vs aflibercept (Eylea) vs bevacizumab (Avastin) for Macular Oedema secondary to Central Retinal Vein Occlusion (CRVO): The LEAVO study. 1. Will individual participant data be available including data dictionaries ? No 2. What data in particular will be shared? All available data published in JAMA Ophthalmology and the NIHR HTA Journal will be available for researchers. In addition the latest version of the protocol and the latest version of the statistical analysis plan will be shared. 3. When will the data be available (start and end dates): Immediately after publication of the NIHR HTA final report and ending 36 months following NIHR report publication. 4. With whom will the data be shared? Any researcher who provides an ethics approved study protocol 5. For what analysis will the data be shared? To achieve the aims of the approved study protocol 6. By what mechanisms will the data be shared? Proposals should be directed to the Chief Investigator, Mr Philip Hykin and if approved, a data sharing agreement will need to be signed with Moorfields Eye Hospital NHS Foundation Trust. After 3 years, the data will be deposited with R&D department, Moorfields Eye Hospital. Mr Philip Hykin Chief Investigator LEAVO May 3rd 2019
