# Supplementary material for: Clinical Effectiveness of Intravitreal Therapy With Ranibizumab vs Aflibercept vs Bevacizumab for Macular Edema Secondary to Central Retinal Vein Occlusion: A Randomized Clinical Trial
Source: JAMA Ophthalmol. 2019 Aug 29;137(11):1256–64. doi: 10.1001/jamaophthalmol.2019.3305 (PMC6865295; doi:10.1001/jamaophthalmol.2019.3305)
Supplement: Journal Club Slides [file jamaophthalmol-137-1256-slides.pptx]

## Slide 1
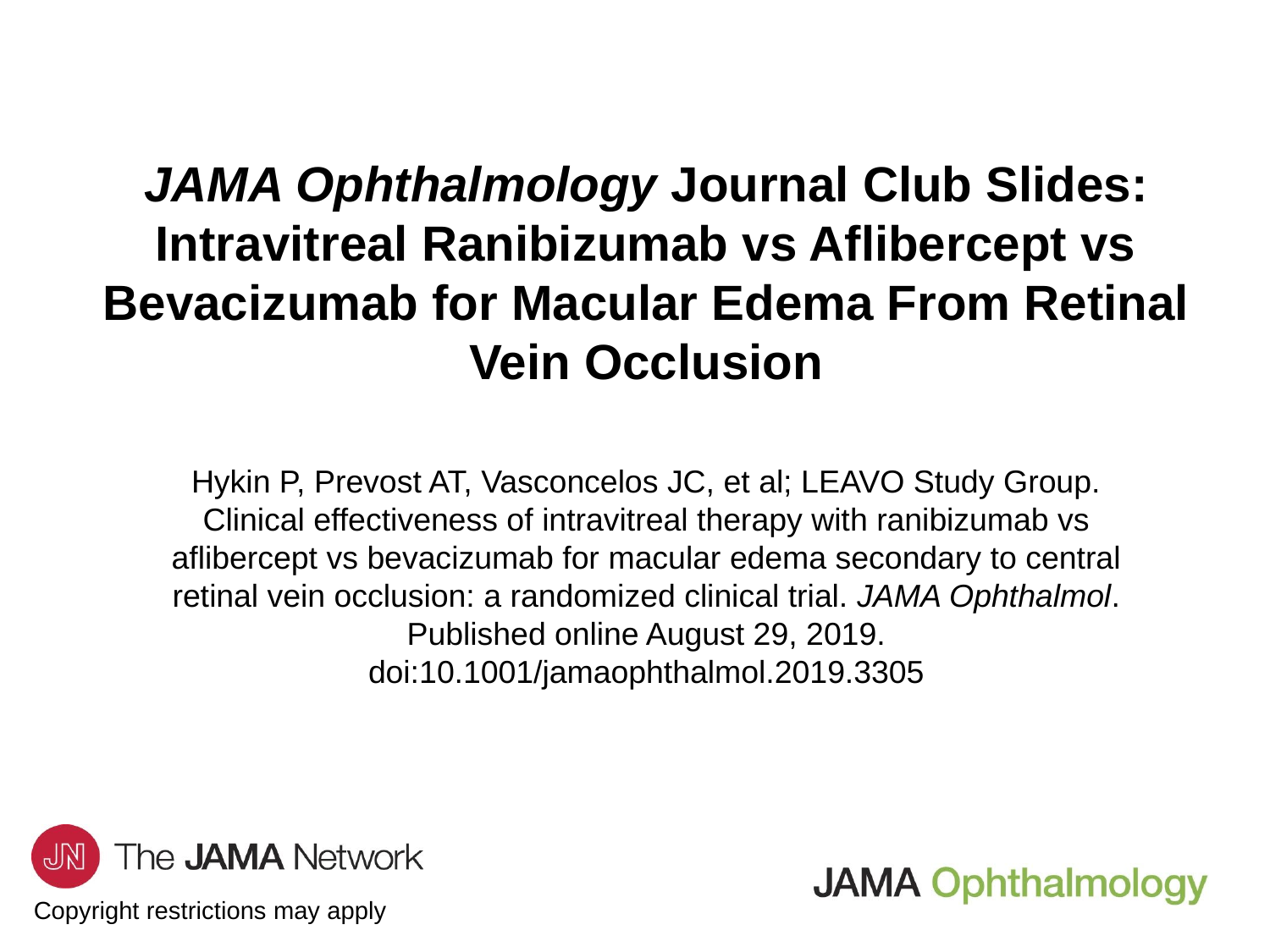

JAMA Ophthalmology Journal Club Slides:
Intravitreal Ranibizumab vs Aflibercept vs Bevacizumab for Macular Edema From Retinal Vein Occlusion
Hykin P, Prevost AT, Vasconcelos JC, et al; LEAVO Study Group. Clinical effectiveness of intravitreal therapy with ranibizumab vs aflibercept vs bevacizumab for macular edema secondary to central retinal vein occlusion: a randomized clinical trial. JAMA Ophthalmol. Published online August 29, 2019. doi:10.1001/jamaophthalmol.2019.3305

## Slide 2
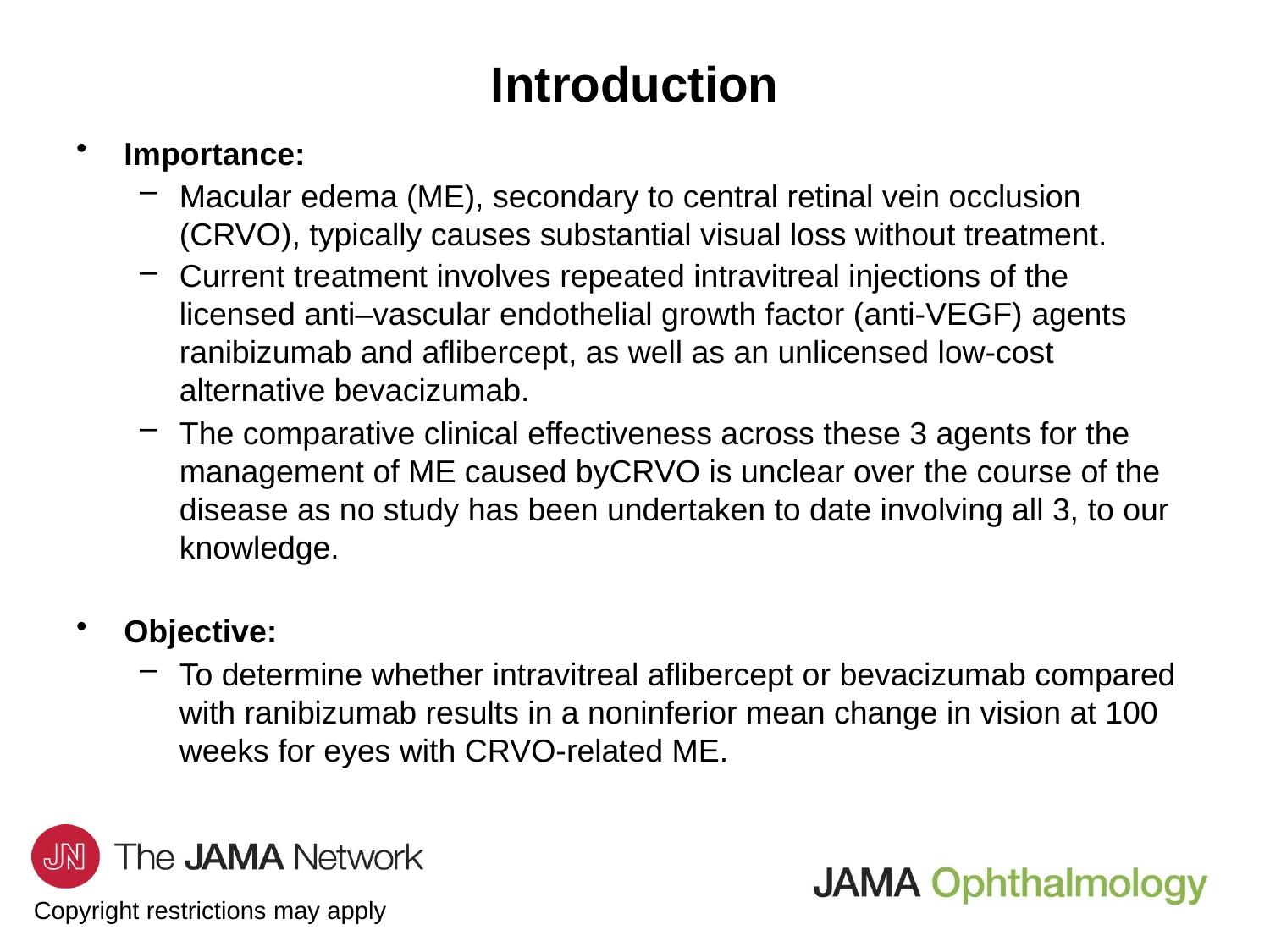

# Introduction
Importance:
Macular edema (ME), secondary to central retinal vein occlusion (CRVO), typically causes substantial visual loss without treatment.
Current treatment involves repeated intravitreal injections of the licensed anti–vascular endothelial growth factor (anti-VEGF) agents ranibizumab and aflibercept, as well as an unlicensed low-cost alternative bevacizumab.
The comparative clinical effectiveness across these 3 agents for the management of ME caused byCRVO is unclear over the course of the disease as no study has been undertaken to date involving all 3, to our knowledge.
Objective:
To determine whether intravitreal aflibercept or bevacizumab compared with ranibizumab results in a noninferior mean change in vision at 100 weeks for eyes with CRVO-related ME.

## Slide 3
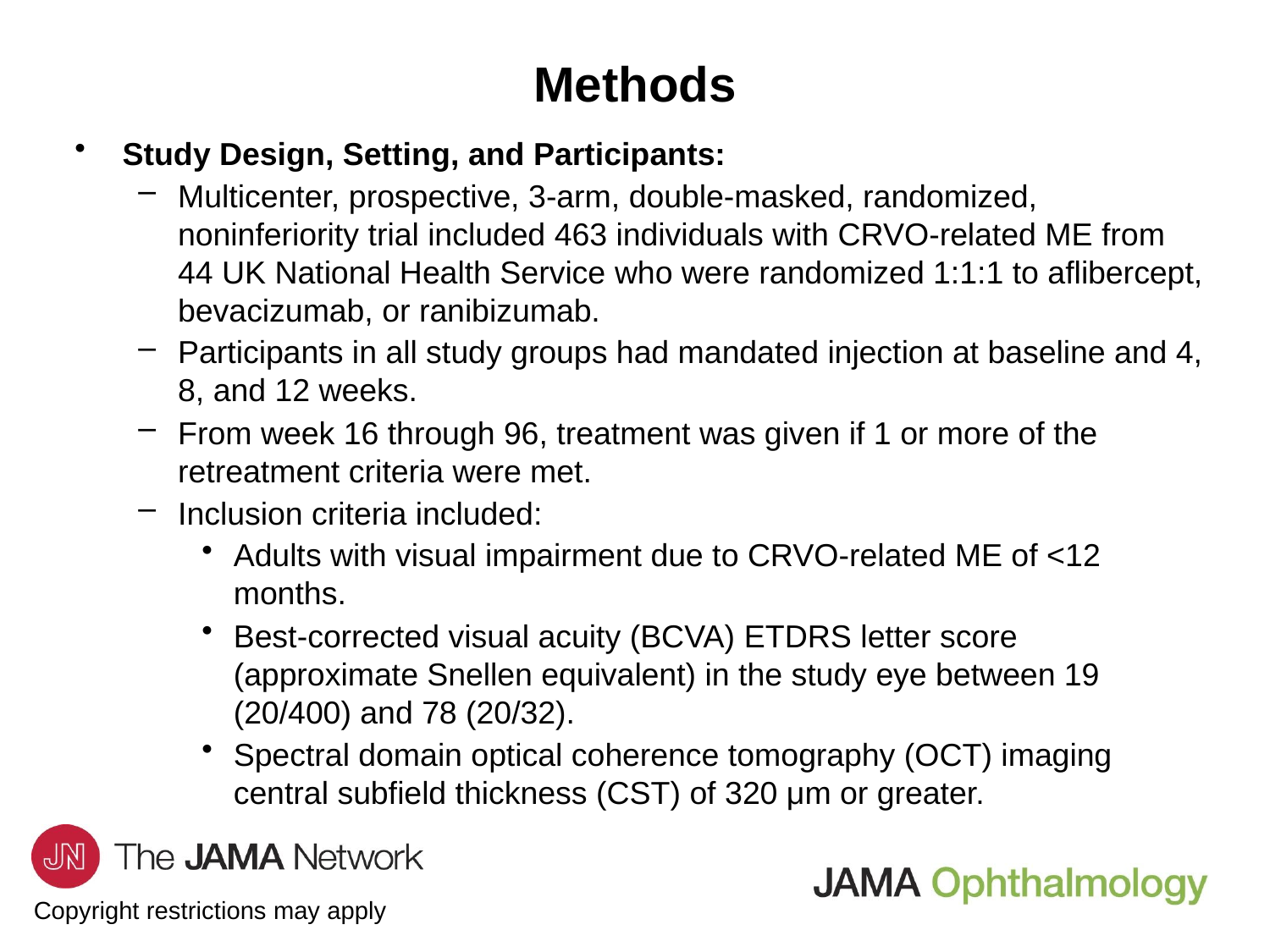

# Methods
Study Design, Setting, and Participants:
Multicenter, prospective, 3-arm, double-masked, randomized, noninferiority trial included 463 individuals with CRVO-related ME from 44 UK National Health Service who were randomized 1:1:1 to aflibercept, bevacizumab, or ranibizumab.
Participants in all study groups had mandated injection at baseline and 4, 8, and 12 weeks.
From week 16 through 96, treatment was given if 1 or more of the retreatment criteria were met.
Inclusion criteria included:
Adults with visual impairment due to CRVO-related ME of <12 months.
Best-corrected visual acuity (BCVA) ETDRS letter score (approximate Snellen equivalent) in the study eye between 19 (20/400) and 78 (20/32).
Spectral domain optical coherence tomography (OCT) imaging central subfield thickness (CST) of 320 μm or greater.

## Slide 4
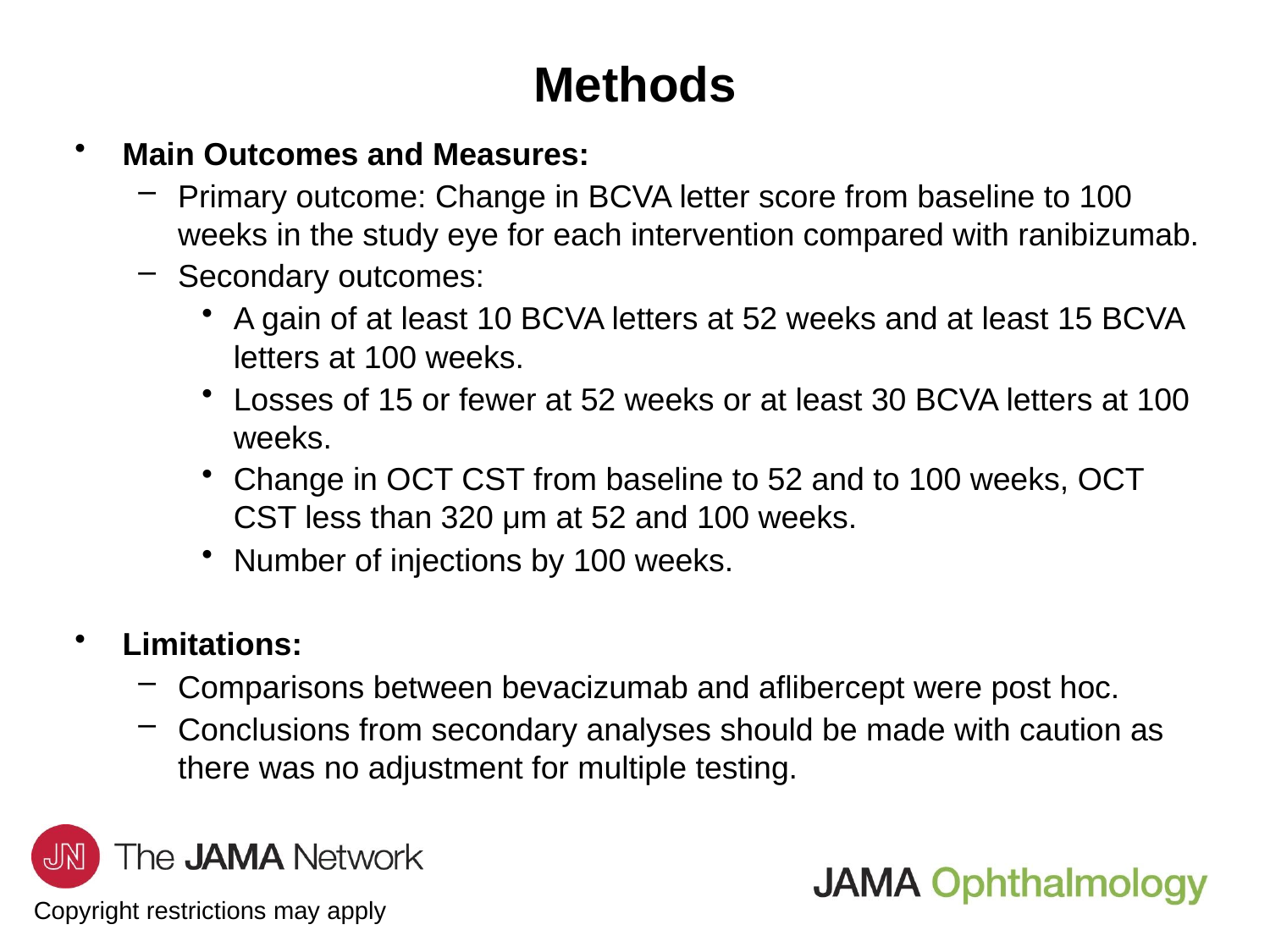

# Methods
Main Outcomes and Measures:
Primary outcome: Change in BCVA letter score from baseline to 100 weeks in the study eye for each intervention compared with ranibizumab.
Secondary outcomes:
A gain of at least 10 BCVA letters at 52 weeks and at least 15 BCVA letters at 100 weeks.
Losses of 15 or fewer at 52 weeks or at least 30 BCVA letters at 100 weeks.
Change in OCT CST from baseline to 52 and to 100 weeks, OCT CST less than 320 μm at 52 and 100 weeks.
Number of injections by 100 weeks.
Limitations:
Comparisons between bevacizumab and aflibercept were post hoc.
Conclusions from secondary analyses should be made with caution as there was no adjustment for multiple testing.

## Slide 5
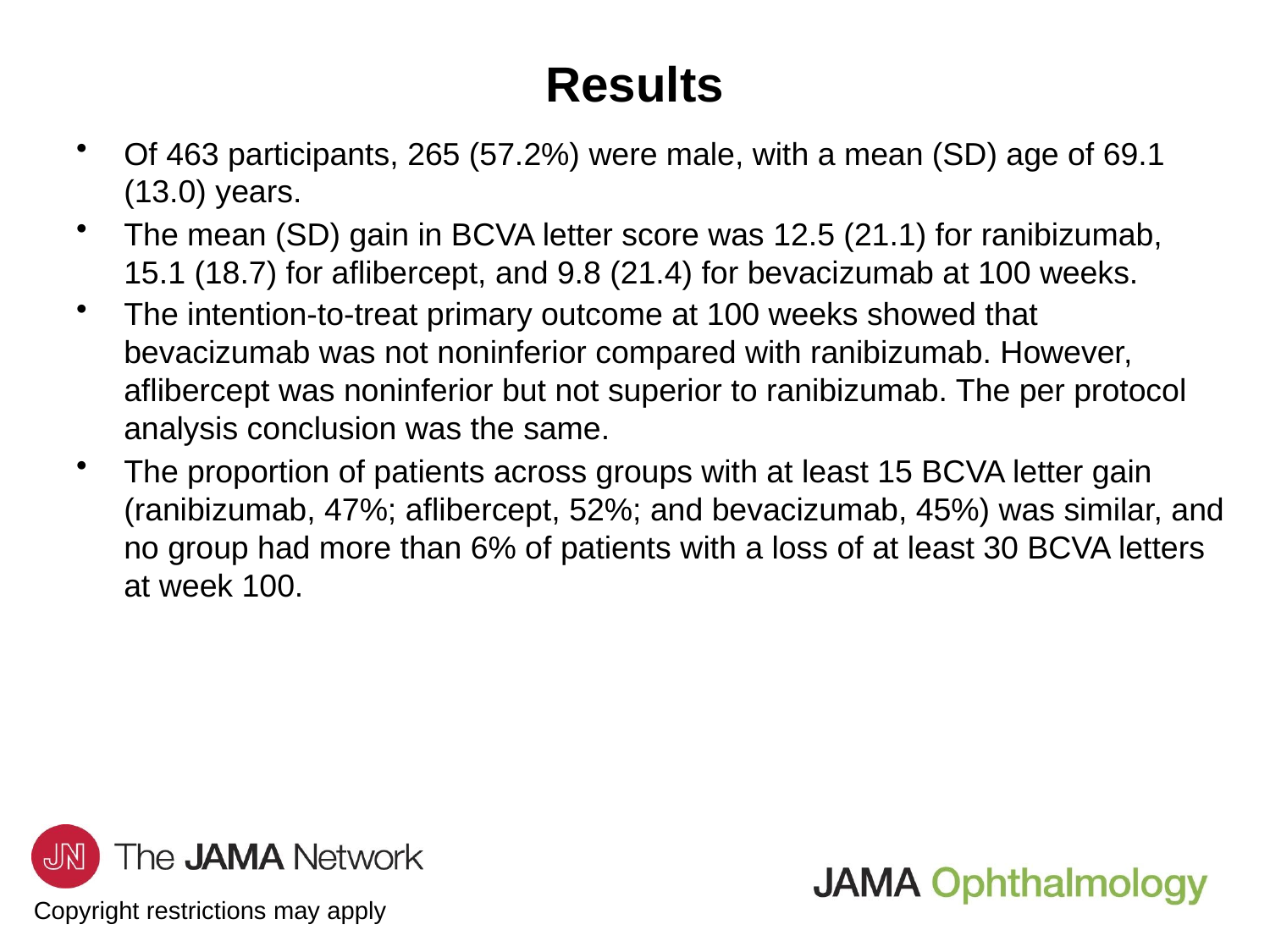

# Results
Of 463 participants, 265 (57.2%) were male, with a mean (SD) age of 69.1 (13.0) years.
The mean (SD) gain in BCVA letter score was 12.5 (21.1) for ranibizumab, 15.1 (18.7) for aflibercept, and 9.8 (21.4) for bevacizumab at 100 weeks.
The intention-to-treat primary outcome at 100 weeks showed that bevacizumab was not noninferior compared with ranibizumab. However, aflibercept was noninferior but not superior to ranibizumab. The per protocol analysis conclusion was the same.
The proportion of patients across groups with at least 15 BCVA letter gain (ranibizumab, 47%; aflibercept, 52%; and bevacizumab, 45%) was similar, and no group had more than 6% of patients with a loss of at least 30 BCVA letters at week 100.

## Slide 6
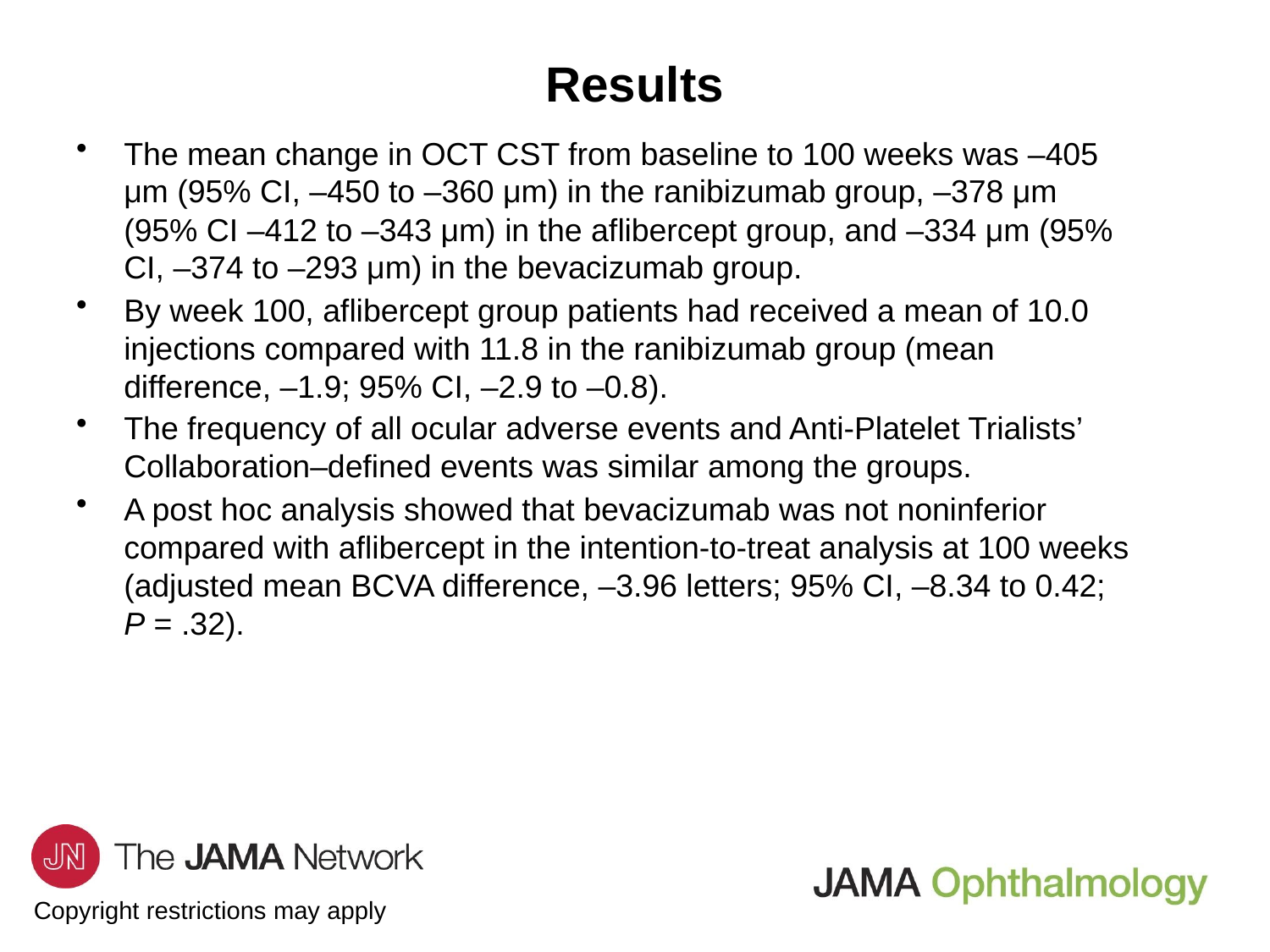

# Results
The mean change in OCT CST from baseline to 100 weeks was –405 μm (95% CI, –450 to –360 μm) in the ranibizumab group, –378 μm (95% CI –412 to –343 μm) in the aflibercept group, and –334 μm (95% CI, –374 to –293 μm) in the bevacizumab group.
By week 100, aflibercept group patients had received a mean of 10.0 injections compared with 11.8 in the ranibizumab group (mean difference, –1.9; 95% CI, –2.9 to –0.8).
The frequency of all ocular adverse events and Anti-Platelet Trialists’ Collaboration–defined events was similar among the groups.
A post hoc analysis showed that bevacizumab was not noninferior compared with aflibercept in the intention-to-treat analysis at 100 weeks (adjusted mean BCVA difference, –3.96 letters; 95% CI, –8.34 to 0.42; P = .32).

## Slide 7
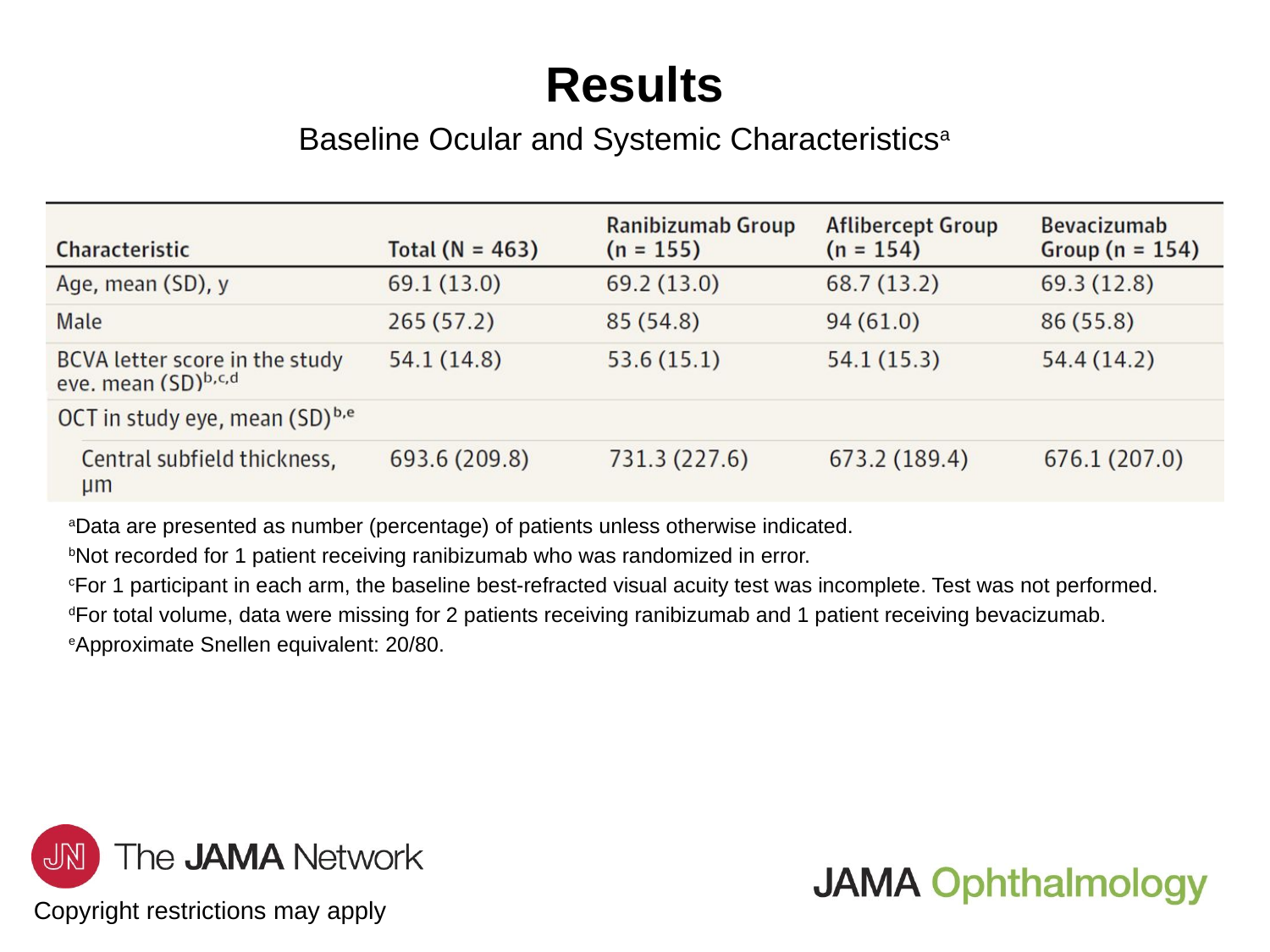

# Results
Baseline Ocular and Systemic Characteristicsa
aData are presented as number (percentage) of patients unless otherwise indicated.
bNot recorded for 1 patient receiving ranibizumab who was randomized in error.
cFor 1 participant in each arm, the baseline best-refracted visual acuity test was incomplete. Test was not performed.
dFor total volume, data were missing for 2 patients receiving ranibizumab and 1 patient receiving bevacizumab.
eApproximate Snellen equivalent: 20/80.

## Slide 8
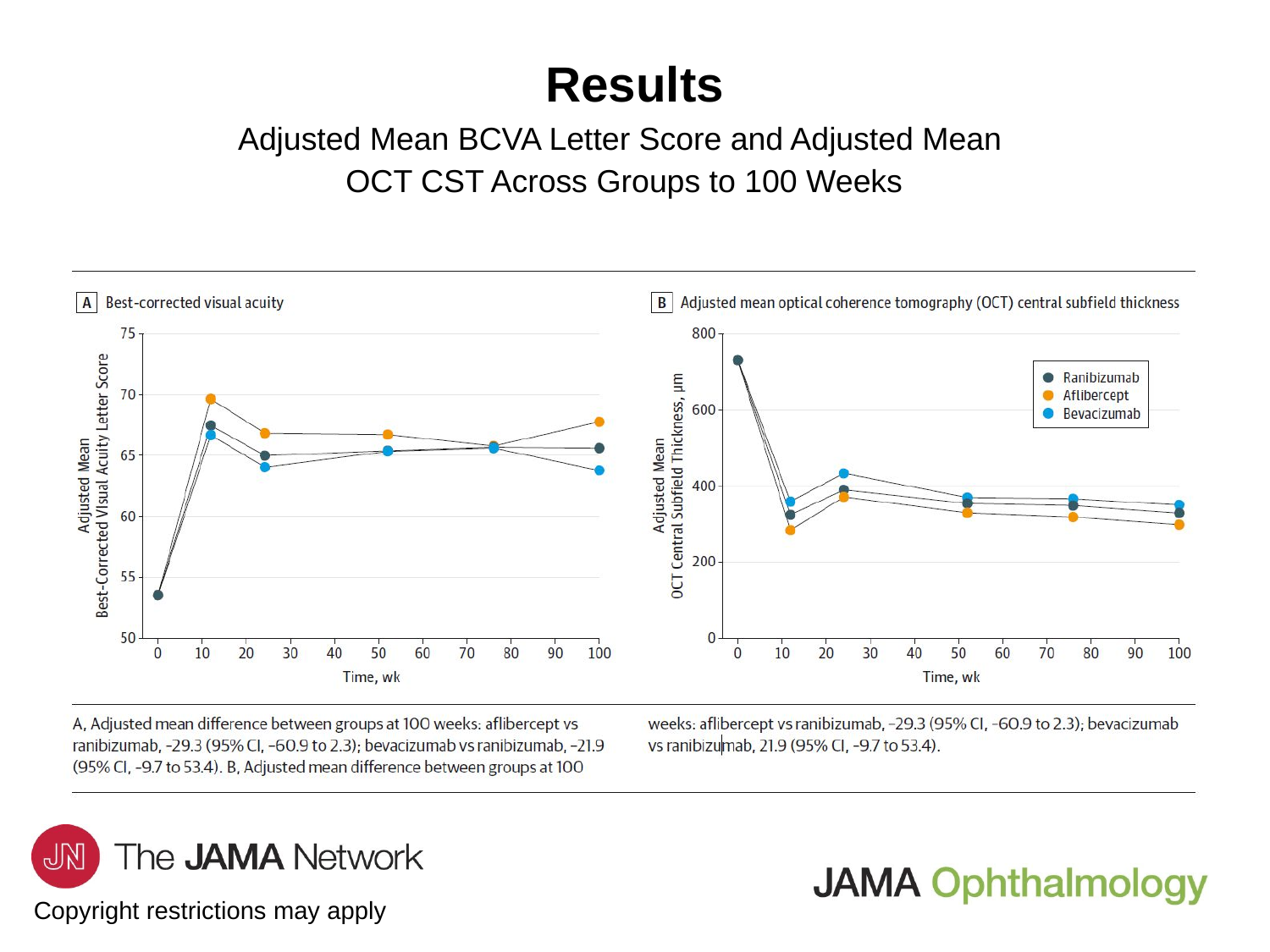

# Results
Adjusted Mean BCVA Letter Score and Adjusted Mean
OCT CST Across Groups to 100 Weeks

## Slide 9
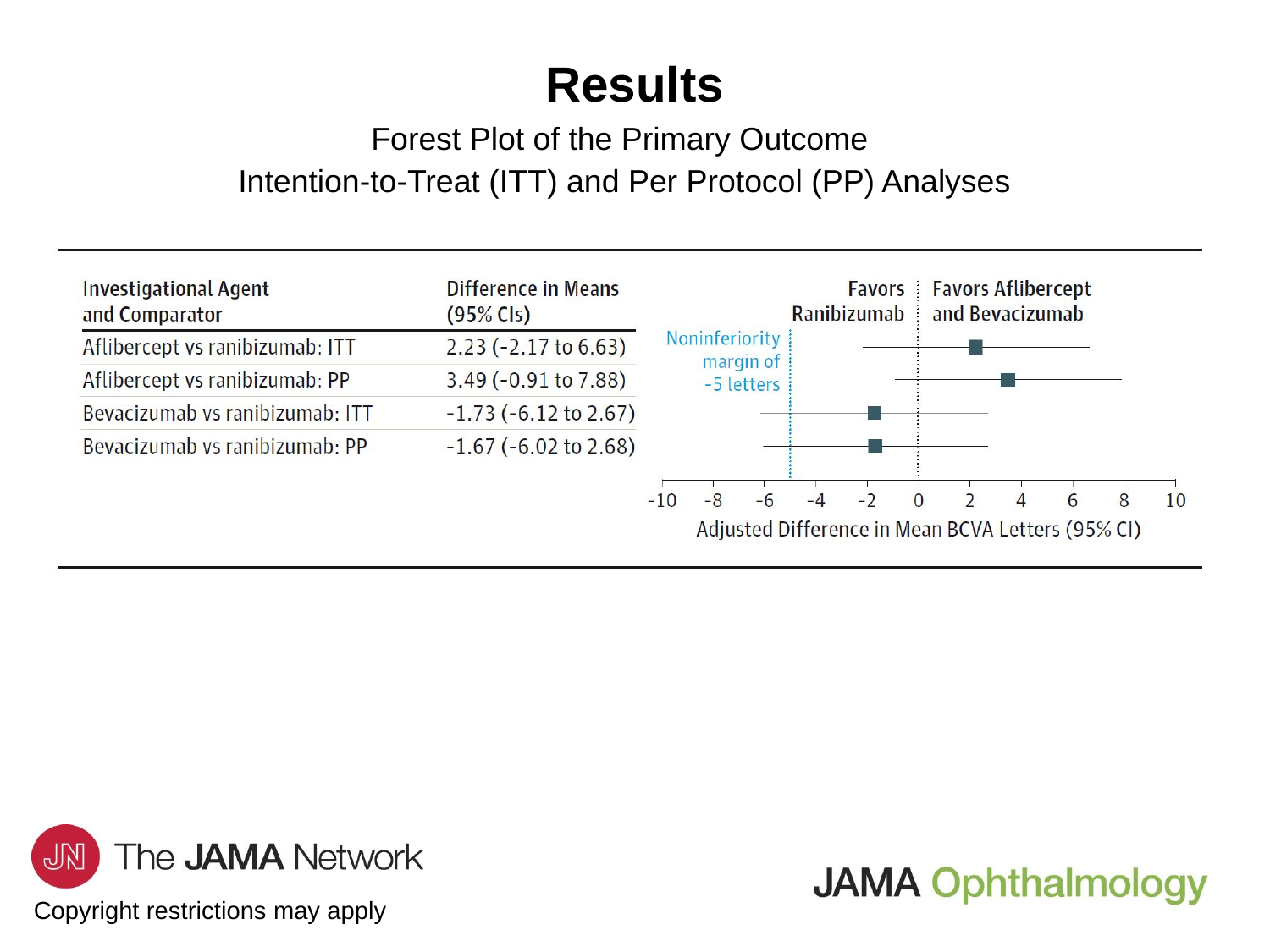

# Results
Forest Plot of the Primary Outcome
Intention-to-Treat (ITT) and Per Protocol (PP) Analyses

## Slide 10
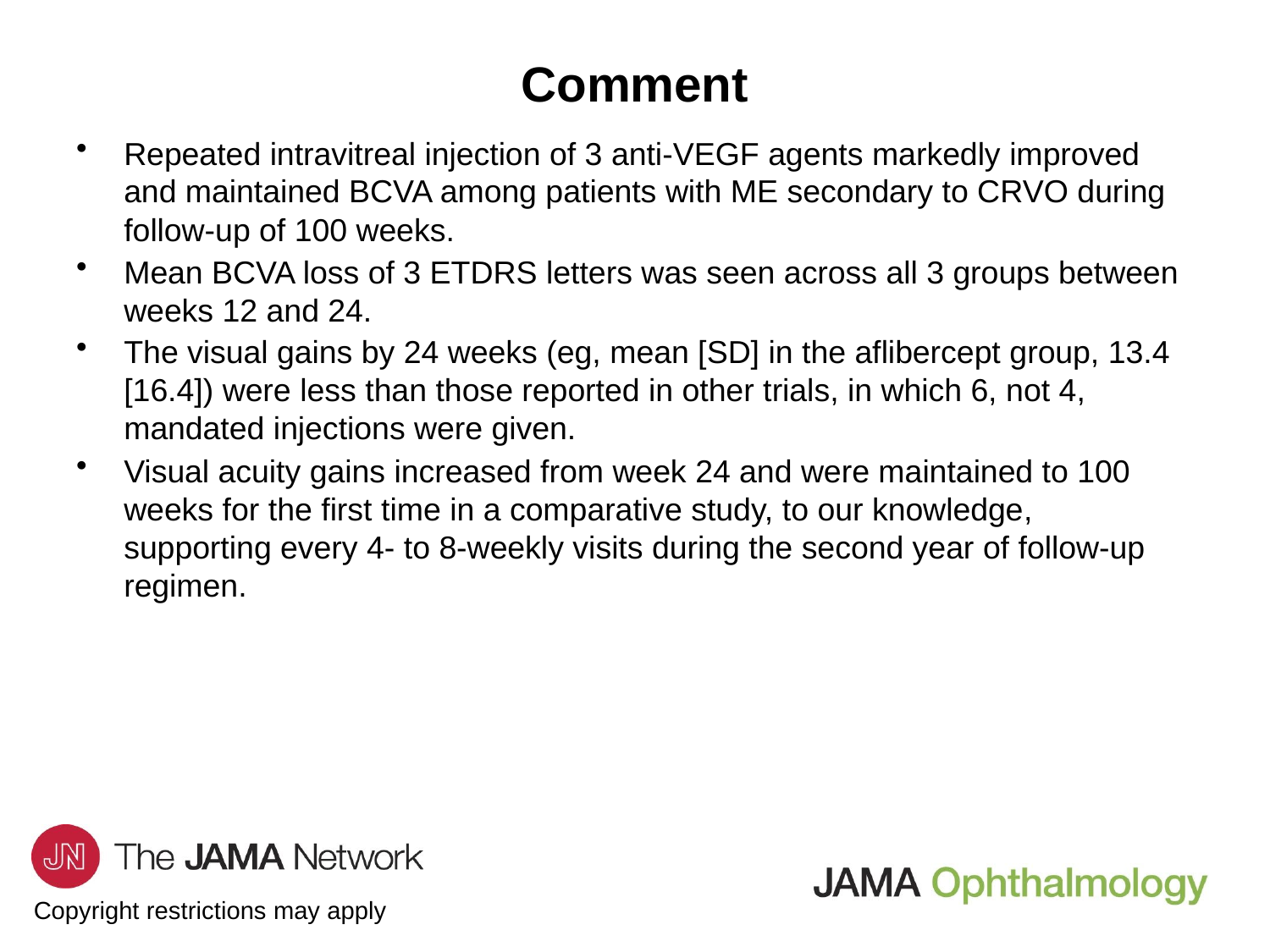

# Comment
Repeated intravitreal injection of 3 anti-VEGF agents markedly improved and maintained BCVA among patients with ME secondary to CRVO during follow-up of 100 weeks.
Mean BCVA loss of 3 ETDRS letters was seen across all 3 groups between weeks 12 and 24.
The visual gains by 24 weeks (eg, mean [SD] in the aflibercept group, 13.4 [16.4]) were less than those reported in other trials, in which 6, not 4, mandated injections were given.
Visual acuity gains increased from week 24 and were maintained to 100 weeks for the first time in a comparative study, to our knowledge, supporting every 4- to 8-weekly visits during the second year of follow-up regimen.

## Slide 11
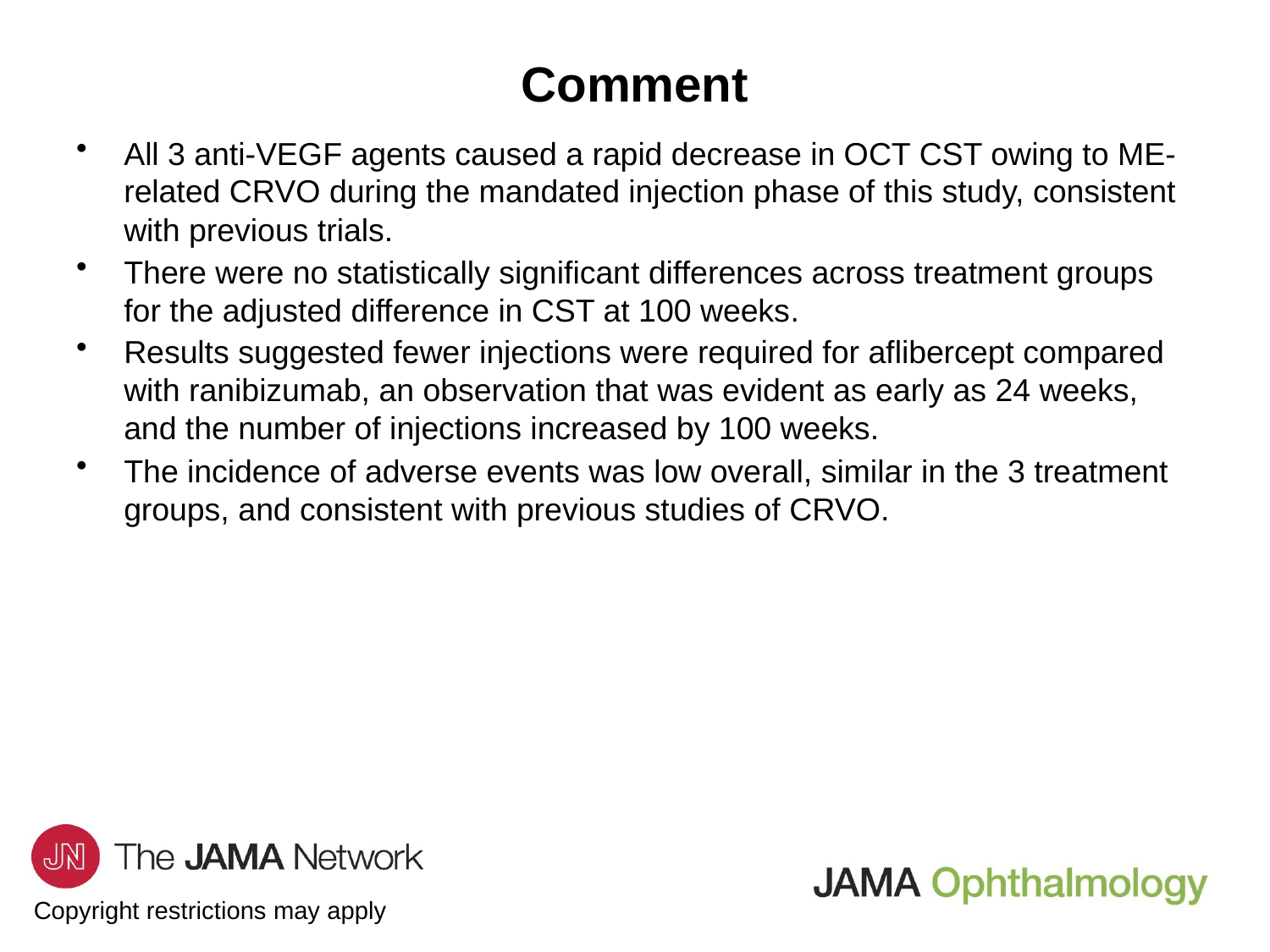

# Comment
All 3 anti-VEGF agents caused a rapid decrease in OCT CST owing to ME-related CRVO during the mandated injection phase of this study, consistent with previous trials.
There were no statistically significant differences across treatment groups for the adjusted difference in CST at 100 weeks.
Results suggested fewer injections were required for aflibercept compared with ranibizumab, an observation that was evident as early as 24 weeks, and the number of injections increased by 100 weeks.
The incidence of adverse events was low overall, similar in the 3 treatment groups, and consistent with previous studies of CRVO.

## Slide 12
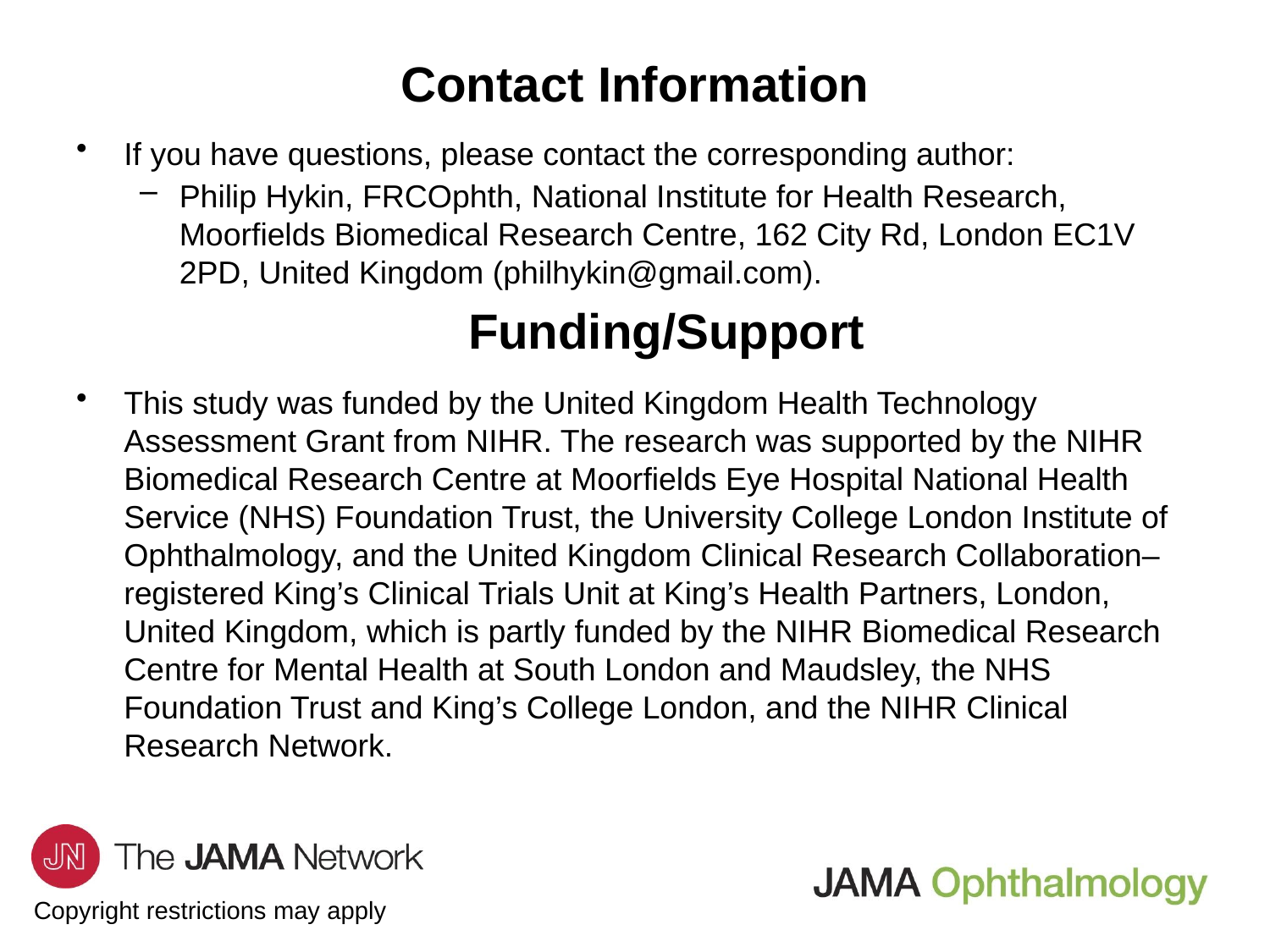

# Contact Information
If you have questions, please contact the corresponding author:
Philip Hykin, FRCOphth, National Institute for Health Research, Moorfields Biomedical Research Centre, 162 City Rd, London EC1V 2PD, United Kingdom (philhykin@gmail.com).
Funding/Support
This study was funded by the United Kingdom Health Technology Assessment Grant from NIHR. The research was supported by the NIHR Biomedical Research Centre at Moorfields Eye Hospital National Health Service (NHS) Foundation Trust, the University College London Institute of Ophthalmology, and the United Kingdom Clinical Research Collaboration–registered King’s Clinical Trials Unit at King’s Health Partners, London, United Kingdom, which is partly funded by the NIHR Biomedical Research Centre for Mental Health at South London and Maudsley, the NHS Foundation Trust and King’s College London, and the NIHR Clinical Research Network.

## Slide 13
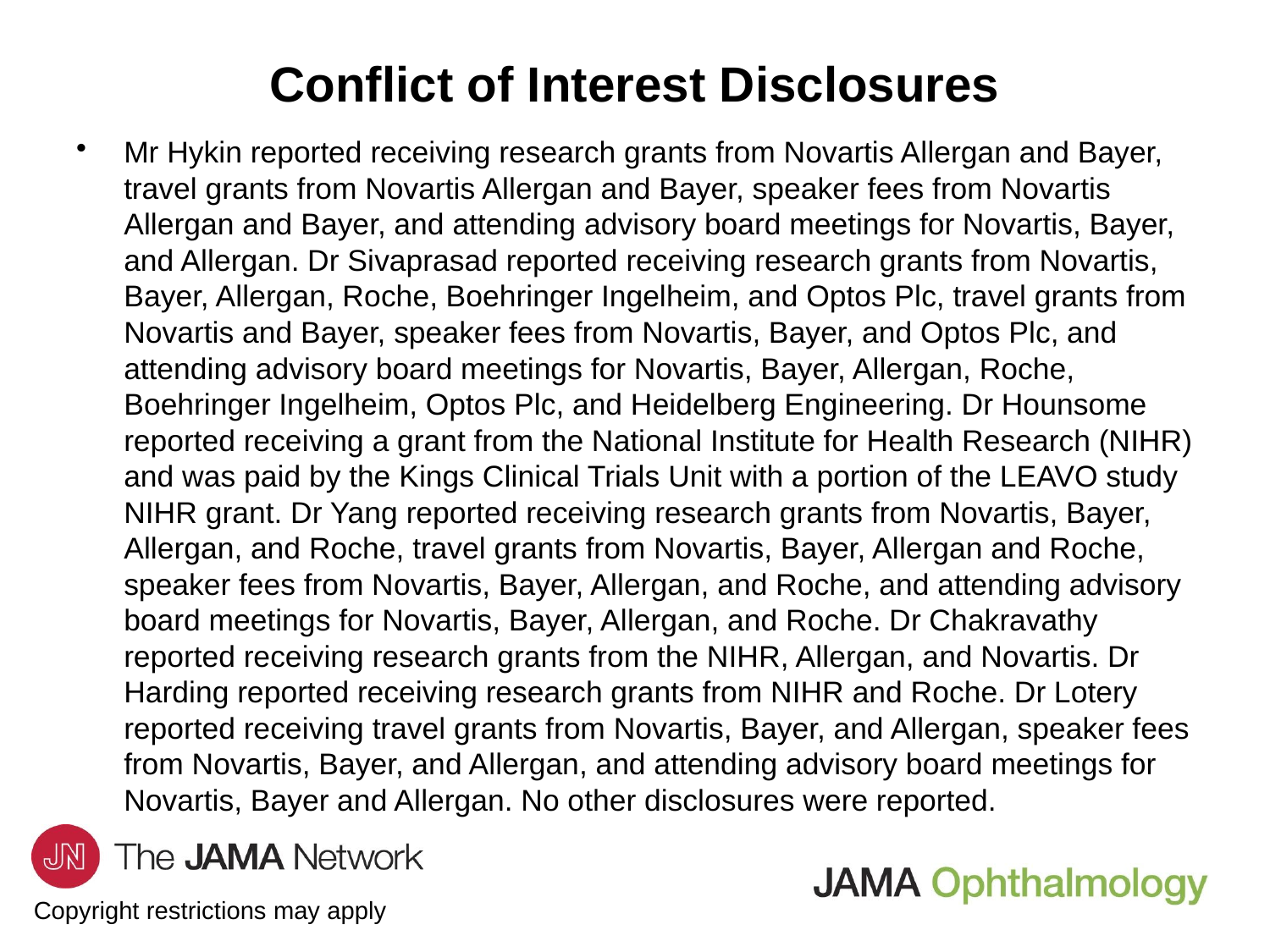

# Conflict of Interest Disclosures
Mr Hykin reported receiving research grants from Novartis Allergan and Bayer, travel grants from Novartis Allergan and Bayer, speaker fees from Novartis Allergan and Bayer, and attending advisory board meetings for Novartis, Bayer, and Allergan. Dr Sivaprasad reported receiving research grants from Novartis, Bayer, Allergan, Roche, Boehringer Ingelheim, and Optos Plc, travel grants from Novartis and Bayer, speaker fees from Novartis, Bayer, and Optos Plc, and attending advisory board meetings for Novartis, Bayer, Allergan, Roche, Boehringer Ingelheim, Optos Plc, and Heidelberg Engineering. Dr Hounsome reported receiving a grant from the National Institute for Health Research (NIHR) and was paid by the Kings Clinical Trials Unit with a portion of the LEAVO study NIHR grant. Dr Yang reported receiving research grants from Novartis, Bayer, Allergan, and Roche, travel grants from Novartis, Bayer, Allergan and Roche, speaker fees from Novartis, Bayer, Allergan, and Roche, and attending advisory board meetings for Novartis, Bayer, Allergan, and Roche. Dr Chakravathy reported receiving research grants from the NIHR, Allergan, and Novartis. Dr Harding reported receiving research grants from NIHR and Roche. Dr Lotery reported receiving travel grants from Novartis, Bayer, and Allergan, speaker fees from Novartis, Bayer, and Allergan, and attending advisory board meetings for Novartis, Bayer and Allergan. No other disclosures were reported.
